# Quantitative analysis of the human T cell palmitome

**DOI:** 10.1038/srep11598

**Published:** 2015-06-26

**Authors:** Eliot Morrison, Benno Kuropka, Stefanie Kliche, Britta Brügger, Eberhard Krause, Christian Freund

**Affiliations:** 1 Freie Universität Berlin, Institut für Chemie und Biochemie, Thielallee 63, 14195 Berlin, Germany; 2 Leibniz-Institut für Molekulare Pharmakologie, Robert-Rössle-Strasse 10, 13125 Berlin, Germany; 3 Otto-von-Guericke University, Institute of Molecular and Clinical Immunology, Leipziger Strasse 44, 39120 Magdeburg, Germany; 4 Heidelberg University Biochemistry Center (BZH)Im Neuenheimer Feld 328 69120 Heidelberg

## Abstract

Palmitoylation is a reversible post-translational modification used to inducibly compartmentalize proteins in cellular membranes, affecting the function of receptors and intracellular signaling proteins. The identification of protein “palmitomes” in several cell lines raises the question to what extent this modification is conserved in primary cells. Here we use primary T cells with acyl-biotin exchange and quantitative mass spectrometry to identify a pool of proteins previously unreported as palmitoylated *in vivo*.

Palmitoylation, the post-translational addition of palmitate to cysteines via a reversible thioester bond, has recently emerged as a powerful mechanism by which eukaryotic cells modulate protein recruitment to, and partitioning within, cellular membranes. Pioneering studies of Ras palmitoylation first highlighted the importance of this modification in cellular signaling, and it has since become clear that a palmitoylation cycle between DHHC palmitoyl acyltransferases (PATs) at the ER and Golgi and palmitoyl protein thioesterases at the plasma membrane dynamically regulates the distribution of proteins in membrane microdomains and between intracellular membranes^1^,^2^,^3^,^4^. In T cells, signaling molecules such as Ras, LAT and PAG/Cbp have been reported as palmitoylated, which has been shown to affect T cell receptor-proximal signaling^5^. Dysfunctional regulation of palmitoylation/depalmitoylation cycling has been linked to a number of human diseases; genes encoding many of the DHHC PAT family members, for example, have been linked to schizophrenia, Huntington’s disease, X-linked mental retardation and various cancers, while a dysfunctional thioesterase was shown to manifest a neurodegenerative phenotype in children^6^,^7^,^8^,^9^,^10^.

Despite these recent insights, many fundamental questions about palmitoylation’s role in cellular signaling remain unanswered. Central to this problem is the identification of palmitoylation targets, which, since early studies in yeast, has been approached using proteomic methods to establish the cellular “palmitome”^1^,^4^. Direct detection of cysteine palmitoylation by mass spectrometry-based approaches, however, is difficult, due to the instability of the thioester bond during sample preparation and MS/MS analysis. Instead, several techniques have been described to enrich palmitoylated proteins for indirect proteomic analysis, most notably acyl-biotin exchange (ABE) and 17-octadecynoic acid (17-ODYA) enrichment. While the former method relies on the selective biotinylation of free thiols following cleavage of the thioester bond by hydroxylamine, the latter technique operates by metabolic labeling and subsequent click chemistry to tag palmitoylated targets^11^,^12^,^13^,^14^. In both cases, an affinity purification step follows, allowing the specific enrichment of palmitoylated proteins. Subsequent mass spectrometric quantification has traditionally relied on spectral counting, but recently has been used with SILAC-labeled cell lines to improve the confidence of quantification^15^. While each method reproducibly enriches a generally overlapping pool of palmitoylated candidates, a significant number of high-confidence candidates are enriched in only one of the two methods, highlighting the limitations and the complementarity of the two approaches^16^.

A fundamental restriction of SILAC-based methods is the requirement of cell lines that are stable in culture, limiting its use in primary cell and tissue studies. This limitation could lead to incomplete or misrepresented views of palmitoylated protein populations, especially in a dynamic, cell-signaling context. For example, one might expect a non-adherent, fast-growing cell culture line such as Jurkat T cells to have different requirements for receptor signaling – conceivably regulated to some degree by palmitoylation – compared to a primary T cell that constantly scans for environmental cues to direct its proliferative, migratory and adhesive properties.

Here we report the coupling of ABE enrichment of palmitoylated proteins with ^16^O/^18^O-labeling for relative quantification of the palmitome of primary human T cells by mass spectrometry^17^. T cells isolated from three healthy donors were pooled for enrichment by ABE (Fig. 1). In brief, after reducing endogenous disulfides by tris(2-carboxyethyl)phosphine (TCEP), free thiols are blocked by N-ethylmaleimide (NEM). Palmitoyl thioester bonds are then cleaved via hydroxylamine (HA), making them available for biotinylation with a sulfhydryl-reactive biotin reagent; biotinylated proteins are subsequently enriched via streptavidin beads. A control sample omits thioester cleavage by HA, preventing biotinylation. Following elution, the HA-treated and -untreated samples are run in separate lanes of an SDS-PAGE gel (Fig. 1). As a proof of principle, we used the detection of LAT, a well-known palmitoylated protein, from primary T cell lysates via Western blot analysis as a positive control (Supplementary Fig. 1). For quantification by LC-MS/MS, tryptic in-gel digestion in the presence of H_2_^18^O (“heavy” water) and H_2_^16^O (“light” water) for HA-treated and -untreated samples, respectively, leads to the enzymatic incorporation of either one (via hydrolysis of the peptide bond by trypsin) or two (via hydrolysis of the acyl-enzyme complex) ^18^O isotopes at the C-terminus of peptides from the HA-treated sample; quantification of the “heavy” intensity by the Mascot software then accounts for both singly and doubly labeled peptides^18^,^19^. After mixing heavy and light samples, detection by mass spectrometry allows for the determination of the ratio of heavy-to-light peptide intensities. This ensures ABE-enriched proteins are distinguished from nonspecific binders during affinity purification, leading to a pool of enriched palmitoylated protein candidates.

While two previous studies have quantified palmitoylated proteins in Jurkat T cells using a 17-ODYA-based proteomics approach, due to the complementarity of the two methods, we chose the ABE enrichment method for quantifying the Jurkat palmitome, allowing a stringent comparison with our primary-cell data^13^,^20^. The SILAC method was used to label Jurkat proteins with heavy (^13^C/^15^N) arginine and (^13^C) lysine in one of the two (+/− HA) samples. This allowed for the mixing of samples prior to affinity purification and SDS-PAGE (Supplementary Fig. 2).

For primary T cells, we defined the criteria for enrichment as a heavy/light isotopic ratio greater than 3.0, as long as at least two unique peptides were used for quantification, in at least two of four replicate experiments. An exception from this stringent criterion was made for proteins that were quantified with only a single peptide, as long as that same unique peptide was also quantified in our Jurkat experiments. Furthermore, since our primary T cell purification protocol may be expected to copurify a minor percentage of contaminating B cells and other lymphocytes, we excluded proteins whose gene expression was not previously detected in mouse primary CD4 + T lymphocytes^21^. Finally, we screened for known proteins containing thioester bonds unrelated to palmitoylation, such as ubiquitin ligases, which would be enriched by the ABE protocol as false positives^11^. Using these criteria, we could define a set of 280 proteins as robustly palmitoylated in primary human T cells (Fig. 2a and Tables S1 and S2).

We evaluated this set of proteins according to the following criteria: (i) presence of at least one predicted palmitoylation site, as predicted by the CSS-Palm algorithm, (ii) identification of these proteins in any of eight previous palmitome studies across various mammalian cell lines, and (iii) the presence of at least one transmembrane helix, as predicted by the TMHMM algorithm (Fig. 2c)^11^,^13^,^20^,^22^,^23^,^24^,^25^,^26^,^27^,^28^. As expected, there is a strong enrichment of transmembrane proteins (64% of the enriched pool), and of proteins involved in intracellular protein transport and, to a lesser degree, vesicle-mediated transport (Figs. 2c and 2d). Moreover, 55% of our enriched proteins were reported in previous palmitome studies across several cell lines, representing a pool of ubiquitously palmitoylated mammalian proteins. Additionally, we found an overlap of 91 enriched proteins that were also identified as palmitoylated in Jurkats previously using the complementary 17-ODYA method^13^. These data are summarized in Supplementary Table S3. Additionally, it must be noted that 46 of the primary-cell proteins we identified as unenriched (and therefore unpalmitoylated) have been reported as palmitoylated in two or more other palmitome studies (across various different cell types) (Fig. 2a, Tables S2 and S4). There could be several reasons for these contradictory results: these proteins might be palmitoylated in other cell types but not primary T cells, or our quantification or scoring algorithms may be stricter than other published studies; in some cases this means we’ve likely rejected true palmitoylated candidates as “false negatives”.

For a quantitative comparison we used the Jurkat palmitome derived from the SILAC-labeled ABE protocol (Table S5 and Supplementary Fig. 2). We found a large common set of 120 palmitoylated proteins in both primary and Jurkat T cells, including well-known palmitoylated proteins such as Lck, H-Ras, N-Ras and LAT, indicating a set of constitutively lipidated molecules important for various aspects of cellular signaling. However, a larger set of proteins is enriched in primary cells than in Jurkats (280 vs. 231), and the primary-cell pool contains a number of proteins (92) not previously reported as palmitoylated; the majority of these (55) have at least one palmitoylation site predicted by the CSS-Palm algorithm. Several others, including CD53, CD37, LIME1 and LMTK2, were previously identified in studies utilizing radioactive ^3^H-palmitic acid, thus independently confirming our findings^29^,^30^,^31^. Not surprisingly, several DHHC palmitoyl transferases, including ZDHHC5 and ZDHHC17, were identified as palmitoylated across our studies. Interestingly, however, ZDHHC18—not previously reported as palmitoylated in earlier studies – was only found palmitoylated in primary cells.

To validate the specific palmitoylation of our enriched pool, we chose several candidates to confirm by Western blotting (Fig. 2b). Among the confirmed palmitoylated proteins was LAT, whose constitutive palmitoylation in T cells is well established^32^. We also confirmed the previously unreported palmitoylation of the T cell surface antigen Ly9 (CD229) and the proteasome inhibitor PI31 subunit PSMF1. Interestingly, when validating the palmitoylation of the PAT ZDHHC18 by Western blot, two bands were seen in the lysate, likely representing the two human isoforms of this enzyme. In the enriched sample, however, only the truncated Isoform 2 was significantly enriched. Given that autopalmitoylation is considered the first catalytic step in the DHHC PAT enzymatic mechanism, this primary cell-specific differential palmitoylation in T cells could suggest cellular mechanistic differences, as has been established in the palmitoylation of the brain-specific isoform of Cdc42^33^,^34^.

The ability to bring sensitive and robust quantitative methods to proteomic palmitoylation studies opens the door to tracking dynamic, global changes for a wide variety of primary cells and organotypic tissue cultures. In this way, the regulatory role palmitoylation cycles play in cellular signaling events may, in time, be elucidated with the same degree of sophistication as phosphorylation has in recent decades.

## Methods

### Cells

Primary CD4 + T cells were isolated from three healthy human donors using a Pan T-cell Isolation Kit and AutoMacs magnetic separation system (Miltenyi Biotec). Approval for these studies was obtained from the Ethics Committee of the Medical Faculty at the Otto-von-Guericke University, Magdeburg, Germany. Informed consent was obtained in accordance with the Declaration of Helsinki. Following isolation, primary T cells were kept overnight at a density of 2 × 10^6^ cells/ml at 37 °C with 5% CO_2_, then pelleted at 300 × g, 4 °C, 10 min and frozen at −80 °C until lysis. Jurkat T cells (clone E6-1) were cultured in RPMI 1640 SILAC medium with 10% dialyzed FBS (SILAC Quantification Kit, Pierce) and either light arginine and lysine or heavy-labeled ^13^C_6_,^15^N_4_-arginine and ^13^C_6_-lysine (Silantes) for 9 days at 37 °C with an atmosphere of 5% CO_2_. Jurkat cells were harvested at a density of 1 × 10^6^ cells/ml by spinning at 300 × g, 4 °C, 10 min, then frozen at −80 °C before lysis.

### Acyl-Biotin Exchange (ABE) Enrichment

The ABE enrichment method used closely follows that originally outlined in Wan *et al.*, 2007, with minor modifications^12^. Cell pellets were lysed for 30 minutes on ice in ABE lysis buffer (50 mM Tris (pH 7.4) (Roth), 150 mM NaCl (Roth), 10 mM MgCl_2_ (AppliChem), 10 mM KCl (Sigma-Aldrich), 500 μM EDTA (Roth), 100 μM Na_3_VO_4_ (Sigma-Aldrich), 20 mM N-ethylmaleimide (NEM) (Thermo), 1 mM PMSF, 1.7% Triton X-100 (Roth), 1 mM tris(2-carboxyethyl)phosphine (TCEP) (Sigma-Aldrich), 100 μM methyl arachidonyl fluorophosphonate (MAFP) (Sigma-Aldrich), cOmplete EDTA-free protease inhibitor cocktail (PI-cocktail) (Roche) at a protein concentration of 3 mg/ml, then spun at 16,100 × g, 4 °C, 10 min to remove cell debris. The addition of TCEP allows for the reduction of all disulfide linkages, while NEM blocks the free thiols from later biotinylation. The inclusion of MAFP, a lipase inhibitor, ensures the palmitoylation state is arrested by inhibiting all palmitoyl protein thioesterase activity. Chloroform-methanol (CM) precipitation steps were performed between each chemical labeling step to ensure complete removal of previous reagents. Lysates were incubated overnight at 4 °C with rotational motion in NEM buffer (50 mM Tris (pH 7.4), 5 mM EDTA, 1% SDS (Roth), 125 mM NaCl, 1 mM PMSF, 0.2% Triton X-100, 20 mM NEM and PI-cocktail). Following removal of NEM by three sequential CM precipitation steps, heavy SILAC samples were incubated for 1 hr at room temperature with rotational motion in +HA buffer (50 mM Tris (pH 7.4), 5 mM EDTA, 125 mM NaCl, 1% SDS, 574 mM hydroxylamine (HA) (Sigma-Aldrich), 820 μM EZ-Link HPDP-biotin (Thermo), 0.2% Triton X-100, 1 mM PMSF and PI-cocktail), while light SILAC samples were incubated in –HA buffer (same as +HA buffer but omitting HA); in a parallel enrichment, the heavy/light labels were also reversed. HA was removed with a single CM precipitation step, then samples were incubated in Biotin Buffer (50 mM Tris (pH 7.4), 5 mM EDTA, 125 mM NaCl, 164 μM EZ-Link HPDP-biotin, 0.2% Triton X-100, 1 mM PMSF and PI-cocktail) for 1 hr at room temperature with rotational motion. Following biotinylation, samples were washed via three sequential CM precipitation steps and resuspended in 50 mM Tris (pH 7.4), 5 mM EDTA, 125 mM NaCl, 0.1% SDS, 0.2% Triton X-100, 1 mM PMSF and PI-cocktail. For SILAC-labeled Jurkat samples, heavy (+HA) and light (−HA) samples were mixed, while primary cell samples were left unmixed. Samples were incubated with streptavidin-agarose beads (Novagen) for 90 min at room temperature with rotational motion. After four sequential washing steps, biotinylated proteins were eluted in buffer containing 1% beta-mercaptoethanol (2-ME) (Roth) at 37 °C for 15 min. Eluted samples were precipitated using trichloroacetic acid (TCA) precipitation, resuspended in SDS-PAGE sample buffer containing 0.2% Triton X-100, boiled for 5 min at 95 °C, then run on a 4–12% Bis-Tris Protein Gel (NuPAGE Novex). Primary cell samples were run in separate lanes (+HA and –HA), side by side. The above enrichment was repeated six times using SILAC-labeled Jurkat cell material and four times using primary T cell material.

### Quantitative LC-MS/MS

For SILAC-labeled samples, gel lanes were cut into 30 equal-sized bands and in-gel tryptic digestion was performed as described^18^. For primary cell samples, neighboring gel lanes were cut into 30 equal-sized bands in a parallel fashion, and protein digestion and in-gel ^16^O/^18^O-labeling was performed as previously described. Briefly, gel bands were incubated with 50 ng trypsin (Promega) in 15 μl 50 mM ammonium bicarbonate buffer in the presence of heavy (H_2_^18^O) and light (H_2_^16^O) water overnight at 37 °C. To limit back-exchange by residual trypsin activity, 10 μl of 0.5% TFA in acetonitrile was added. Matching heavy and light samples were then mixed before drying the samples under vacuum. Samples were resuspended in 6 μl 0.1% (v/v) TFA and 5% (v/v) acetonitrile. Peptides were analyzed by a reversed-phase capillary liquid chromatography system (Ultimate 3000 nanoLC system (Thermo Scientific) connected to an Orbitrap Elite mass spectrometer (Thermo Scientific). LC separations were performed on a capillary column (Acclaim PepMap100, C18, 3 μm, 100 Å, 75 μm i.d. × 25 cm, Thermo Scientific) at an eluent flow rate of 300 nL/min using a linear gradient of 3–25% B in 53 min with further increase to 80% B in 80 min. Mobile phase A contained 0.1% formic acid in water, and mobile phase B contained 0.1% formic acid in acetonitrile. Mass spectra were acquired in a data-dependent mode with one MS survey scan with a resolution of 60,000 (Orbitrap Elite) and MS/MS scans of the 15 most intense precursor ions in the linear trap quadrupole. Mass spectra of ^16^O/^18^O-labeled tryptic peptides for LAT and ZDHHC18 are shown in Fig. S3, showing resolution of singly and doubly labeled peptides.

### Proteomic Data Analysis

#### Human primary T cells

Identification and quantification of ^16^O/^18^O-labeled samples was performed using Mascot Distiller (version 2.4.3.3) software. Data were searched against the Uniprot human protein database (July 2013). The mass tolerance of precursor and sequence ions was set to 10 ppm and 0.35 Da, respectively. For quantification, only unique peptides identified better than the homology were used. Additionally, a minimum of two peptide ratios was required for quantification. As mentioned earlier, this criterion was extended to include proteins quantified with only a single peptide if the same peptide was previously used for quantification in our SILAC measurements.

#### Jurkat T cells

Identification and quantification of SILAC samples was performed using MaxQuant (version 1.4.1.1) software. Data were searched against the Uniprot human protein database (July 2013). The criteria for identification were set to at least 1 unique peptide and at least 2 razor + unique peptides, while the criterion for quantification was a ratio count of at least 2. Based on the distribution of quantified proteins, the criteria for enrichment was established as a heavy/light ratio of at least 1.75 in at least two of the four replicate experiments.

#### Data Evaluation

Protein quantification data from Mascot Distiller (primary T cells, 4 replicate experiments) and MaxQuant (Jurkat T cells, 6 replicate experiments) were scored using a custom Python script. As mentioned above, a protein was considered enriched in a single experiment if it was quantified with a heavy:light intensity ratio of at least 3.0 (primary T cells) or 1.75 (Jurkat T cells). Across the replicate experiments, a protein was considered enriched if it had an enriched score in at least two the replicate experiments; proteins with contradictory scores (equal number of enriched and unenriched) were not included in the final enriched pool. Identified proteins were analyzed for predicated palmitoylation sites using CSS-Palm (version 3.0), with a “High” threshold and a cutoff score of 1.0. Prediction of transmembrane domains was performed using TMHMM Server (version 2.0).

### Western Blot

Western blot samples were eluted and separated by SDS-PAGE as described above, then blotted to nitrocellulose membranes using standard procedures. After blocking the membrane in 5% (w/v) fat-free milk powder in TBS-T (TBS with 0.5% Tween-20), membranes were incubated with primary antibodies overnight at 4 °C. Membranes were then incubated with secondary HRP-coupled antibodies for 1 hr at room temperature, and visualized using an Intas Advanced Imager. Primary antibodies used were rabbit polyclonal anti-LAT (Upstate (Millipore)), mouse monoclonal anti-CD229/SLAMF3 (R&D Systems), rabbit polyclonal anti-PSMF1 (gift from Prof. Kreuger), and rabbit monoclonal anti-ZDHHC18 (Abcam).

## Additional Information

**How to cite this article**: Morrison, E. *et al.* Quantitative analysis of the human T cell palmitome. *Sci. Rep.* **5**, 11598; doi: 10.1038/srep11598 (2015).

## Supplementary Material

Supplementary Information

## Figures and Tables

**Figure 1:**
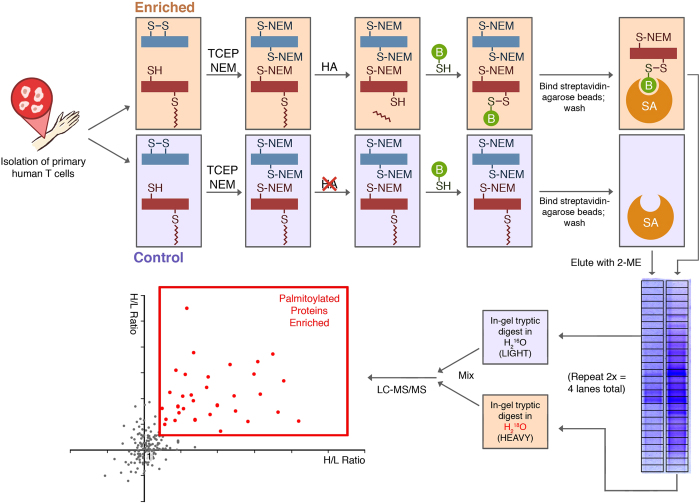
Experimental workflow for the detection of palmitoylated proteins from primary human T cells. Pooled lysates from healthy human donors are initially divided into “Enriched” and “Control” samples. Disulfide bonds are reduced by tris(2-carboxyethyl)phosphine (TCEP), and free thiols are blocked by N-ethylmaleimide (NEM). Following cleavage of the palmitoyl thioester bond by hydroxylamine (HA) in the enriched sample, previously palmitoylated thiols are biotinylated using the sulfhydryl-reactive EZ-Link HPDP-biotin. Control samples omit HA cleavage, and remain unbiotinylated. Following enrichment via streptavidin-agarose beads and elution by beta-mercaptoethanol (2-ME), enriched and control samples are run in parallel lanes of an SDS-PAGE gel. These lanes are cut into equal-sized bands and subjected to tryptic digest in either heavy (H_2_^18^O) or light (H_2_^16^O) water, providing the isotopic label. After digestion, samples are mixed and measured by LC-MS/MS; evaluating the isotopic heavy/light intensity ratio gives rise to an enriched pool representing the population of palmitoylated proteins in the cell.

**Figure 2:**
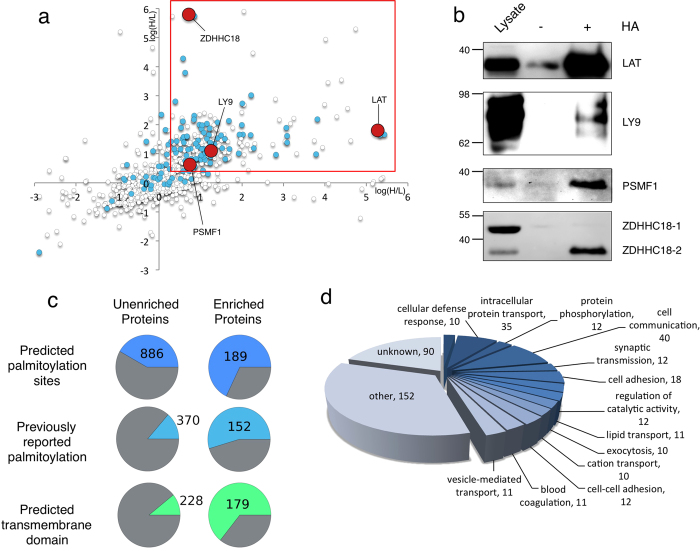
A distinct set of proteins are palmitoylated in primary cells. (**a**) Representative scatter plot of two of four enriched pools of palmitoylated proteins from primary T cells; x- and y-axes represent heavy-to-light isotopic ratios (log10). In total, 280 proteins were found to be palmitoylated. Highlighted are palmitoylation candidates selected for confirmation by Western blot (red). Also shown: “canonical” palmitoylated proteins, reported in at least two previous palmitome studies (blue). (**b**) Confirmation of selected palmitoylation candidates by Western blot using the indicated antibodies. Interestingly, only the truncated Isoform 2 of ZDHHC18, a palmitoylacyl transferase previously unreported as palmitoylated, is palmitoylated, suggesting the possibility of isoform-dependent, differential activation states. (**c**) Summary and evaluation of palmitoylated proteins in primary T cells. Enriched and unenriched pools were evaluated by: (i) prediction of palmitoylation motifs by CSS-Palm algorithm, (ii) previous reports in earlier palmitome studies across various mammalian cell lines, and (iii) prediction of transmembrane domains by the TMHMM algorithm. A clear enrichment is seen in all three criteria. (**d**) Biological functions of primary T cell palmitoylation candidates identified in this study (Gene Ontology annotations). For clarity, groups with fewer than 10 members were pooled under the “Other” category.
