# Supplementary material for: Quantitative analysis of the human T cell palmitome
Source: Sci Rep. 2015 Jun 26;5:11598. doi: 10.1038/srep11598 (PMC4650600; doi:10.1038/srep11598)
Supplement: Supplementary Information [file srep11598-s1.pdf]

Supplementary Information

Quantitative analysis of the human T cell palmitome

Eliot Morrison[1], Benno Kuropka[1,2], Stefanie Kliche[3], Britta Brügger[4], Eberhard Krause[2*] & Christian Freund[1*]

[1]Freie Universität Berlin, Institut für Chemie und Biochemie, Thielallee 63, 14195 Berlin, Germany

Correspondence: christian.freund@fu-berlin.de, ekrause@fmp-berlin.de

[2]Leibniz-Institut für Molekulare -harmakologie, Robert-Rössle-Strasse 10, 13125 Berlin, Germany

[3]Otto-von-Guericke University, Institute of Molecular and Clinical Immunology, Leipziger Strasse 44, 39120 Magdeburg, Germany

[4]Heidelberg University Biochemistry Center (BZH)Im Neuenheimer Feld 328 69120 Heidelberg.

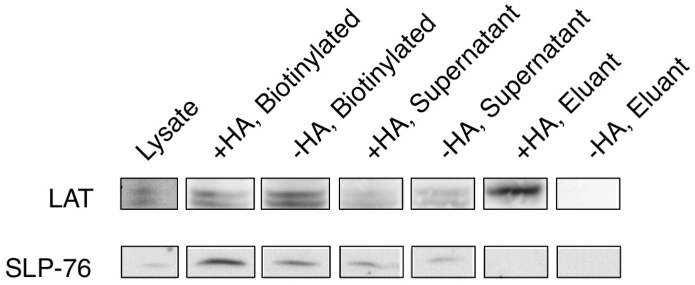

Figure S1. Confirmation of ABE enrichment method by Western blotting. LAT, a canonical palmitoylated protein, was used as a positive control. SLP-76, a protein not reported as palmitoylated, and lacking predicted palmitoylation sites, was used as a negative control. Antibodies used: rabbit polyclonal anti-LAT (Upstate (Millipore)) and Santa Cruz SLP-76 Antibody (F-7). As expected, LAT is enriched in the +HA eluant (but not –HA), while SLP-76 is not detected in either sample.

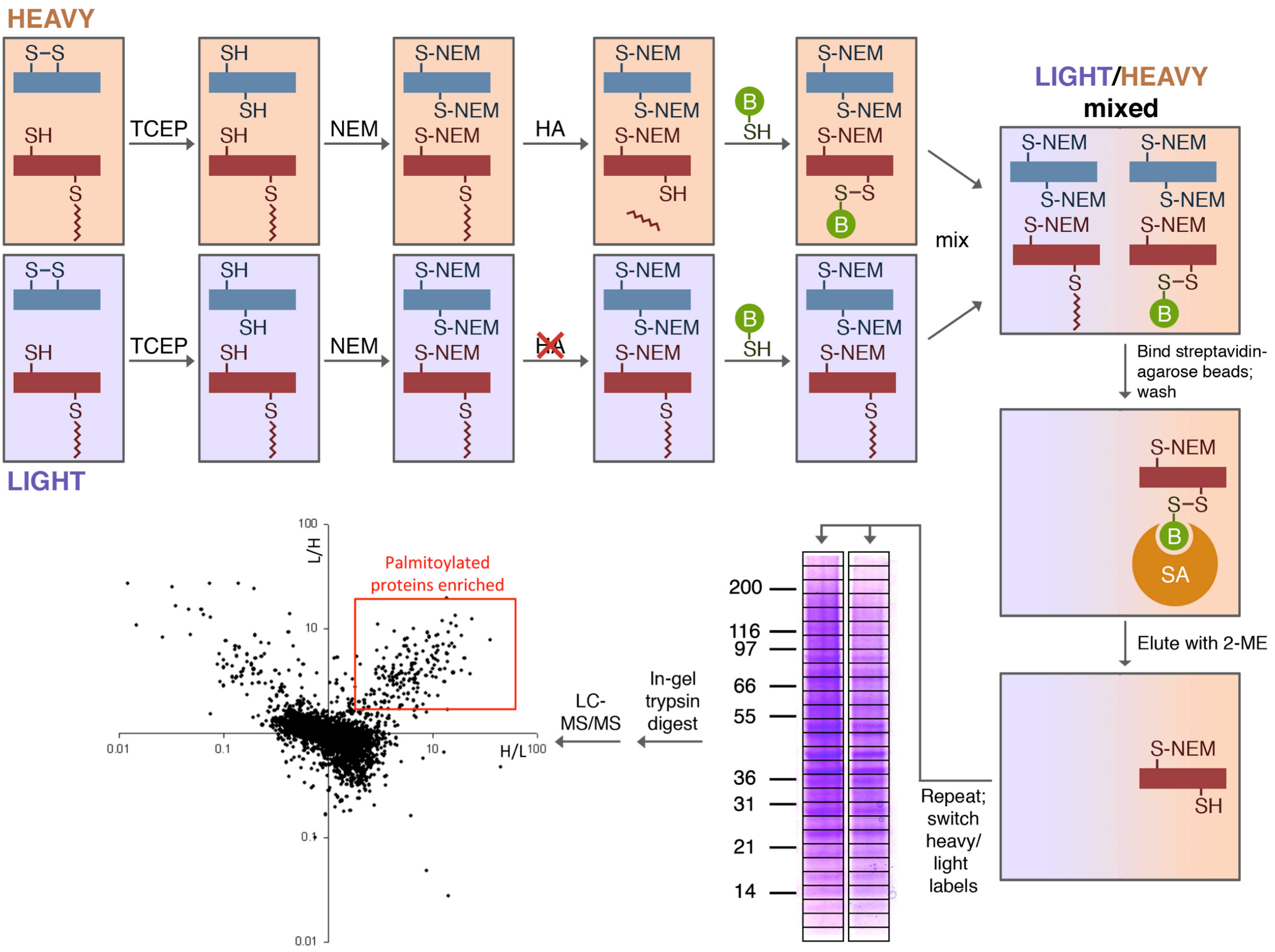

Figure S2. Use of ABE enrichment with SILAC-labeled Jurkat T cells for quantitative analysis of palmitoylated proteins. The use of SILAC labeling allows for the samples to be mixed prior to affinity purification.

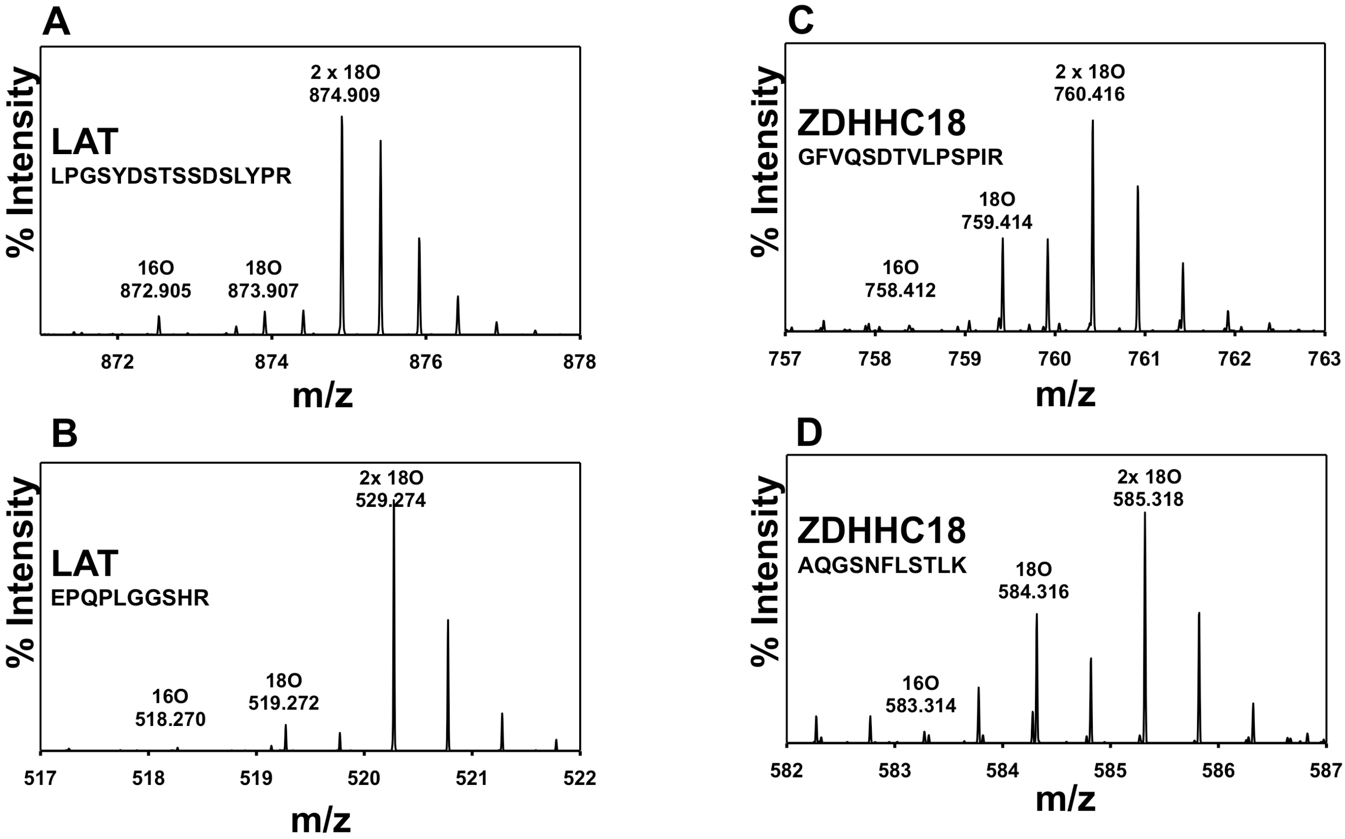

Figure S3. Representative mass spectra of 16O/18O labeled tryptic peptides of LAT and ZDHHC18. Isotope pattern of LAT (A, B) and ZDHHC18 (C, D) peptides showing the doubly charged precursor ions with heavy/light ratios greater than 20, indicating enriched palmitoylated protein candidates.

Supplementary Table 1

Palmitoylated proteins enriched in human primary T cells. Using quantification by 16O/18O-labeling, Isotopic ratios were measured (n=4). Ratios greater than 3.0 are considered "enriched" (red).

UniProt Accession Number	Gene Name	Protein	Isotopic Ratio (n = 4)			
			Sample 1	Sample 2	Sample 3	Sample 4
O15439	ABCC4	Multidrug resistance-associated protein 4	161.601	13.985	21.020	25.074
O95870	ABHD16A	Abhydrolase domain-containing protein 16A	8.587	8.046	18.340	1.607
Q96GS6	ABHD17A	Alpha/beta hydrolase domain-containing protein 17A	113.725	491.195	16.662	9.878
Q5VST6	ABHD17B	Alpha/beta hydrolase domain-containing protein 17B	50.605	7.445	7.335	11.517
P09110	ACAA1	3-ketoacyl-CoA thiolase, peroxisomal	21.969	6.987	3.502	0.291
P24752	ACAT1	Acetyl-CoA acetyltransferase, mitochondrial	10.891	23.318	9.186	6.836
Q9BWD1	ACAT2	Acetyl-CoA acetyltransferase, cytosolic	7.030	7.467	8.061	9.250
O14734	ACOT8	Acyl-coenzyme A thioesterase 8	267156.863	-	520795.660	3.601
P33121	ACSL1	Long-chain-fatty-acid--CoA ligase 1	-	-	6.423	4.915
O43707	ACTN4	Alpha-actinin-4	0.722	-	695.841	7.364
O14672	ADAM10	Disintegrin and metalloproteinase domain-containing protein 10	19.330	45.067	7.675	8.736
P78536	ADAM17	Disintegrin and metalloproteinase domain-containing protein 17	7.873	6.438	-	17.550
O43306	ADCY6	Adenylate cyclase type 6	-	-	918625.678	5.109
P51828	ADCY7	Adenylate cyclase type 7	8.497	21.918	13.450	39.616
Q9NUQ2	AGPAT5	1-acyl-sn-glycerol-3-phosphate acyltransferase epsilon	13.995	4.810	0.953	3.992
P43353	ALDH3B1	Aldehyde dehydrogenase family 3 member B1	135.049	20.770	21.222	-
Q02252	ALDH6A1	Methylmalonate-semialdehyde dehydrogenase [acylating], mitochondrial	1.029	3.921	4.843	1028.771
Q86YT9	AMICA1	Junctional adhesion molecule-like	55228.758	200084.872	0.024	4.160
P58335	ANTXR2	Anthrax toxin receptor 2	6.763	139.465	1721.157	-
Q63HQ0	AP1AR	AP-1 complex-associated regulatory protein	5.014	-	-	12.825
Q9NXU5	ARL15	ADP-ribosylation factor-like protein 15	2.655	26.756	13.700	15.763
Q8N6S5	ARL6IP6	ADP-ribosylation factor-like protein 6-interacting protein 6	31.765	28.962	6.340	1.346
P98196	ATP11A	Probable phospholipid-transporting ATPase IH	22.328	160.429	4.723	0.673
Q9Y2G3	ATP11B	Probable phospholipid-transporting ATPase IF	15.980	44.918	15.277	5.276
Q8NB49	ATP11C	Probable phospholipid-transporting ATPase IG	10.915	4.415	-	0.911
Q9Y487	ATP6V0A2	V-type proton ATPase 116 kDa subunit a isoform 2	6.203	3.416	2.390	4.235
Q9Y679	AUP1	Ancient ubiquitous protein 1	15.351	30.405	22.655	2.479
P15291	B4GALT1	Beta-1,4-galactosyltransferase 1	40.997	12.565	4.336	-
Q9UBV7	B4GALT7	Beta-1,4-galactosyltransferase 7	36.160	35.561	15.635	387096.774
O15155	BET1	BET1 homolog	7.460	40.951	-	-
Q10589	BST2	Bone marrow stromal antigen 2	99.837	268.194	-	-
Q8N357	C2orf18	Solute carrier family 35 member F6	8.709	0.449	2000000.000	3897.123
Q9HA72	CALHM2	Calcium homeostasis modulator protein 2	801.961	148.462	7.555	55.920
P27824	CANX	Calnexin	10.025	7.950	4.662	5.214
O15484	CAPN5	Calpain-5	-	3.787	5.298	4.105
A6NI79	CCDC69	Coiled-coil domain-containing protein 69	0.635	162.296	18.705	72.424
Q8ND76	CCNY	Cyclin-Y	25.842	119.436	6.886	17.105
P51681	CCR5	C-C chemokine receptor type 5	9.575	222.151	-	0.040
P48509	CD151	CD151 antigen	105.392	46.022	198.156	155.013
Q8TD46	CD200R1	Cell surface glycoprotein CD200 receptor 1	5.264	9.735	-	-
P26842	CD27	CD27 antigen	6.728	4.197	-	-
P16671	CD36	Platelet glycoprotein 4 (Fragment)	17.500	8.112	23.251	9.651
P11049	CD37	Leukocyte antigen CD37	9803.922	498.833	39.385	24.821
P28907	CD38	ADP-ribosyl cyclase 1	16.748	75.875	8.561	4.714
P04234	CD3D	T-cell surface glycoprotein CD3 delta chain	3.584	169.913	3.497	1.555
P01730	CD4	T-cell surface glycoprotein CD4	557.516	21.303	6.579	3.562
P16070	CD44	CD44 antigen (Fragment)	10.229	6.270	13.787	2.688
P19397	CD53	Leukocyte surface antigen CD53	10375.817	87417.781	29.382	527.201
P08962	CD63	CD63 antigen	1176.471	78.909	7.769	101.482
P09564	CD7	T-cell antigen CD7	38.497	11.269	44.810	5.105
P21854	CD72	B-cell differentiation antigen CD72	111.928	797793.338	-	-
P27701	CD82	CD82 antigen	1233.660	24.337	9.447	2.144
Q01151	CD83	CD83 antigen	38.145	17.399	-	-
P21926	CD9	CD9 antigen	2.807	-	9.750	7.352
P40200	CD96	T-cell surface protein tactile	14.436	-	11.812	3.660
P48960	CD97	CD97 antigen	19444.444	187.609	5.920	152.572
P14209	CD99	CD99 antigen	9.355	99.279	-	1.767
Q8TCZ2	CD99L2	CD99 antigen-like protein 2	87.582	26.034	-	0.408
Q5VV42	CDKAL1	Threonylcarbamoyladenosine tRNA methylthiotransferase	-	-	10.311	24.621
Q07065	CKAP4	Cytoskeleton-associated protein 4	6.022	6.650	18.882	6.864
Q9NY35	CLDND1	Claudin domain-containing protein 1	8.685	39.593	5.776	4.809
Q6PJW8	CNST	Consortin	9.379	10.632	14.181	2.660
Q86VU5	COMTD1	Catechol O-methyltransferase domain-containing protein 1 (Fragment)	-	28.559	-	18.195
Q9HCP0	CSNK1G1	Casein kinase I isoform gamma-1	272875.817	46.361	34.250	109.154
P78368	CSNK1G2	Casein kinase I isoform gamma-2	26.993	22.342	13.345	23.435
Q9Y6M4	CSNK1G3	Casein kinase I isoform gamma-3	4.739	38.808	51.139	364.778
Q9GZU7-2	CTDSP1	Carboxy-terminal domain RNA polymerase II polypeptide A small phosphatase 1	29.020	20.161	11.143	21.325
Q9GZU7	CTDSP1	Carboxy-terminal domain RNA polymerase II polypeptide A small phosphatase 1	76.160	-	-	35.998
O14595	CTDSP2	Carboxy-terminal domain RNA polymerase II polypeptide A small phosphatase 2	9.845	7.208	4.872	7.286
P04839	CYBB	Cytochrome b-245 heavy chain	6.739	12.540	4.727	6.962
Q9Y4D1	DAAM1	Disheveled-associated activator of morphogenesis 1	-	12.536	64.376	5.983
Q8NCG7	DAGLB	Sn1-specific diacylglycerol lipase beta	5.753	14.802	12.311	14.054
Q8IWE4	DCUN1D3	DCN1-like protein 3	-	3.285	63.472	7.806
Q9P202	DFNB31	Whirlin	-	435.179	7811.935	-
P52429	DGKE	Diacylglycerol kinase epsilon	60.065	78.061	-	1.657
O75190-3	DNAJB6	DnaJ homolog subfamily B member 6	4.409	-	-	11.186
Q9H3Z4	DNAJC5	DnaJ homolog subfamily C member 5	3.697	32.251	-	0.090
P42892	ECE1	Endothelin-converting enzyme 1	4.242	5.648	7.374	1.469
Q14156	EFR3A	Protein EFR3 homolog A	7.650	6.675	13.081	42.781
Q7L2H7	EIF3M	Eukaryotic translation initiation factor 3 subunit M	-	-	70.127	23.592
Q9NYP7	ELOVL5	Elongation of very long chain fatty acids protein 5	20.907	97.857	-	0.435
P54852	EMP3	Epithelial membrane protein 3 (Fragment)	10.850	21.409	-	6.284
Q9Y227	ENTPD4	Ectonucleoside triphosphate diphosphohydrolase 4	8.366	7.749	-	308.195
Q96RT1	ERBB2IP	Protein LAP2	162.418	44.388	10.300	31.037
Q969X5	ERGIC1	Endoplasmic reticulum-Golgi intermediate compartment protein 1	8.742	7.513	5.118	2.874
Q96RQ1	ERGIC2	Endoplasmic reticulum-Golgi intermediate compartment protein 2	5.110	-	-	4.467
Q9Y282	ERGIC3	Endoplasmic reticulum-Golgi intermediate compartment protein 3	82.925	10.278	14.828	70.732
P34910	EVI2B	Protein EVI2B	5.288	12.238	25.931	2.371
Q92817	EVPL	Envoplakin	-	41.417	22.304	-
Q8N128	FAM177A1	Protein FAM177A1	3.268	9.419	-	-

UniProt Accession Number	Gene Name	Protein	Isotopic Ratio (n = 4)			
			Sample 1	Sample 2	Sample 3	Sample 4
Q9NUQ9	FAM49B	Protein FAM49B	25.833	34.585	2.233	6.500
P25445	FAS	Tumor necrosis factor receptor superfamily member 6	8.050	21.918	-	2.241
Q9Y3I1	FBXO7	F-box only protein 7	-	19.111	5.291	1.188
P09769	FGR	Tyrosine-protein kinase Fgr	106.863	4.772	-	17.655
O75955	FLOT1	Flotillin-1	33.252	15.374	6.807	13.888
Q14254	FLOT2	Flotillin-2	8.636	17.976	9.910	318.919
Q9Y2H6	FNDC3A	Fibronectin type-III domain-containing protein 3A	41748.366	28.071	969258.590	5.001
P06241	FYN	Tyrosine-protein kinase Fyn	11.552	2.667	5.526	5666.957
Q10472	GALNT1	Polypeptide N-acetylgalactosaminyltransferase 1	8.521	7.106	1.391	-
Q9UG22	GIMAP2	GTPase IMAP family member 2	33.791	-	3.482	3.012
P29992	GNA11	Guanine nucleotide-binding protein subunit alpha-11	4.256	6216.847	-	31.194
Q03113	GNA12	Guanine nucleotide-binding protein subunit alpha-12	-	101.613	15.555	11682.650
Q14344	GNA13	Guanine nucleotide-binding protein subunit alpha-13	87.337	46.255	13.132	5.983
P09471-2	GNAO1	Isoform Alpha-2 of Guanine nucleotide-binding protein G(o) subunit alpha	23.725	72.926	4.727	1.216
P50148	GNAQ	Guanine nucleotide-binding protein G(q) subunit alpha	6601.307	153.469	150.633	5675.676
P63092	GNAS	Guanine nucleotide-binding protein G(s) subunit alpha isoforms short	8.431	9.092	6.011	-
P19086	GNAZ	Guanine nucleotide-binding protein G(z) subunit alpha	1781.046	168.152	4.582	195.031
Q7Z5G4	GOLGA7	Golgin subfamily A member 7	6.518	2.858	5.993	-
Q2TAP0	GOLGA7B	Golgin subfamily A member 7B	0.016	29.090	5.436	-
Q14789	GOLGB1	Golgin subfamily B member 1	-	3.253	7.291	0.253
Q99795	GPA33	Cell surface A33 antigen	19.763	26.247	23.555	5.605
Q9NPR9	GPR108	Protein GPR108	99673.203	-	-	22.616
P32249	GPR183	G-protein coupled receptor 183	5.566	-	4.745	3452.485
Q15743	GPR68	Ovarian cancer G-protein coupled receptor 1	88.480	23.955	19.107	1979.076
Q6ZVF9	GPRIN3	G protein-regulated inducer of neurite outgrowth 3	30.556	8.500	7.790	3.553
Q9BSH5	HDHD3	Haloacid dehalogenase-like hydrolase domain-containing protein 3	-	3.367	0.678	3.142
O75146	HIP1R	Huntingtin-interacting protein 1-related protein	-	-	547.197	54.333
P30504	HLA-C	HLA class I histocompatibility antigen, Cw-4 alpha chain	24.069	4.078	-	-
P30501	HLA-C	HLA class I histocompatibility antigen, Cw-2 alpha chain	-	-	8.347	30.166
P13598	ICAM2	Intercellular adhesion molecule 2	1094.771	26.926	-	-
Q01628	IFITM3	Interferon-induced transmembrane protein 3	5.023	14.021	-	-
Q969P0	IGSF8	Immunoglobulin superfamily member 8	643.382	760.025	6.647	9.102
Q9H0X4	ITFG3	Protein ITFG3	108.742	-	9.353	-
P23229	ITGA6	Integrin alpha-6	101.144	18.542	5.110	-
O43736	ITM2A	Integral membrane protein 2A	-	100.191	55.552	59.895
P23458	JAK1	Tyrosine-protein kinase JAK1	40.302	6.964	5.566	0.354
Q9BX67	JAM3	Junctional adhesion molecule C	8.595	10.401	142.025	7.636
Q9Y4C1	KDM3A	Lysine-specific demethylase 3A	-	-	3.558	158674.804
Q8IZA0	KIAA0319L	Dyslexia-associated protein KIAA0319-like protein	46.127	6.843	-	-
Q9BY89	KIAA1671	Uncharacterized protein KIAA1671	11.855	-	-	3.720
Q8IYS2	KIAA2013	Uncharacterized protein KIAA2013	15.123	12.476	5.251	1.776
Q8NBE8	KLHL23	Kelch-like protein 23	-	6.208	71.826	3.902
O43561	LAT	Linker for activation of T-cells family member 1	187908.497	63.993	3.797	1.762
P06239	LCK	Tyrosine-protein kinase Lck	12.933	6.172	5.139	7.344
Q9H400	LIME1	Lck-interacting transmembrane adapter 1	7.288	46.213	3.164	1.774
Q9BU23	LMF2	Lipase maturation factor 2	3.910	6.622	3.242	1.954
Q8IWU2	LMTK2	Serine/threonine-protein kinase LMTK2	-	-	41229.656	71665.214
Q9UIQ6	LNPEP	Leucyl-cystinyl aminopeptidase	14.444	12.773	36.087	34.403
A6NHZ5	LRRC14B	Leucine-rich repeat-containing protein 14B	125816.993	-	596745.027	-
Q8N386	LRRC25	Leucine-rich repeat-containing protein 25	2083.333	10.011	-	0.006
Q86X29	LSR	Lipolysis-stimulated lipoprotein receptor	8823.529	16.367	6.268	246730.602
Q9HBG7	LY9	T-lymphocyte surface antigen Ly-9 (Fragment)	17.835	12.661	1112.116	263.819
P20645	M6PR	Cation-dependent mannose-6-phosphate receptor	191.912	15.398	42.278	6.193
Q68D91	MBLAC2	Metallo-beta-lactamase domain-containing protein 2	3.306	14.241	6.148	4.158
Q9P1T7	MDFIC	MyoD family inhibitor domain-containing protein	5.533	12.627	4.676	1.605
Q9NU22	MDN1	Midasin	25.065	67.282	9.693	3.263
Q6ZSS7	MFSD6	Major facilitator superfamily domain-containing protein 6	11.667	7.250	-	3.064
Q14165	MLEC	Malectin	11.773	8.006	2.933	6.223
Q9BRT2	MNF1	Mitochondrial nucleoid factor 1	17.190	4.789	7.866	-
Q9NZW5	MPP6	MAGUK p55 subfamily member 6	8.709	28.581	-	-
Q8N565	MREG	Melanoregulin	-	8.523	-	100261.552
P11836	MS4A1	B-lymphocyte antigen CD20	25163.399	3522.173	8.018	50.366
P03915	MT-ND5	NADH-ubiquinone oxidoreductase chain 5	7.233	15.824	-	265911.072
Q86UE4	MTDH	Protein LYRIC	12.010	14.804	30.090	7.524
Q13614	MTMR2	Myotubularin-related protein 2	-	-	4.141	46.800
Q96S97	MYADM	Myeloid-associated differentiation marker	22.263	19.697	6.033	2.386
Q9NZM1	MYOF	Myoferlin	-	3.862	882459.313	-
P18440	NAT1	Arylamine N-acetyltransferase 1	34.436	13.664	3.826	3.514
Q6PIU2	NCEH1	Neutral cholesterol ester hydrolase 1	47.680	11.400	2.938	3.385
Q92542	NCSTN	Nicastrin	11.119	5.394	-	-
Q00653	NFKB2	Nuclear factor NF-kappa-B p100 subunit	3.531	6.372	-	0.481
O15118	NPC1	Niemann-Pick C1 protein	28.056	18.593	12.593	1.762
P01111	NRAS	GTPase NRas	1.141	225.122	9.262	9.329
Q96D31	ORAI1	Calcium release-activated calcium channel protein 1	5.653	7.887	4.778	1.339
Q9NWU1	OXSM	3-oxoacyl-[acyl-carrier-protein] synthase, mitochondrial	10.482	8.820	4.231	2.806
Q99572	P2RX7	P2X purinoceptor 7	270.915	8.496	12.676	27.384
Q9NWQ8	PAG1	Phosphoprotein associated with glycosphingolipid-enriched microdomains 1	29.436	4.952	-	2.444
Q9H6A9	PCNXL3	Pecanex-like protein 3	-	-	15.078	8.928
O00330	PDHX	Pyruvate dehydrogenase protein X component, mitochondrial	40.106	32.782	15.497	59.669
P16284	PECAM1	Platelet endothelial cell adhesion molecule	18.342	9.533	3.452	3.448
Q9UPP1	PHF8	Histone lysine demethylase PHF8	-	-	3.306	26.652
Q9BTU6	PI4K2A	Phosphatidylinositol 4-kinase type 2-alpha	3.908	38.320	18.459	-
Q8TCG2	PI4K2B	Phosphatidylinositol 4-kinase type 2-beta	20.302	14.882	-	-
O15162	PLSCR1	Phospholipid scramblase 1	1.440	66.264	12.716	3.167
Q9NRY6	PLSCR3	Phospholipid scramblase 3	1225.490	41.608	9.342	16.931
O15031	PLXNB2	Plexin-B2	15.556	72.990	6.629	601.656
A8CG34	POM121C	Nuclear envelope pore membrane protein POM 121C	-	131.827	11.703	4.520
Q96T49	PPP1R16B	Protein phosphatase 1 regulatory inhibitor subunit 16B	184.232	29492.892	12.430	13.566
P55345	PRMT2	Protein arginine N-methyltransferase 2	4.007	4.857	1.934	61.081
Q9UNM6	PSMD13	26S proteasome non-ATPase regulatory subunit 13	0.000	-	22.297	2310.375
Q92530	PSMF1	Proteasome inhibitor PI31 subunit	-	-	5.559	4.234
O43586	PSTPIP1	Proline-serine-threonine phosphatase-interacting protein 1	-	3.762	0.197	4.761
Q9P035	PTPLAD1	Very-long-chain (3R)-3-hydroxyacyl-[acyl-carrier protein] dehydratase 3	10.735	9.410	1.129	3.520

UniProt Accession Number	Gene Name	Protein	Isotopic Ratio (n = 4)			
			Sample 1	Sample 2	Sample 3	Sample 4
P10114	RAP2A	Ras-related protein Rap-2a	3.727	130.236	14.792	6.700
P61225	RAP2B	Ras-related protein Rap-2b	3.833	19308.296	14.937	10.410
Q9Y3L5	RAP2C	Ras-related protein Rap-2c	2.479	639.720	7.125	6.103
Q00765	REEP5	Receptor expression-enhancing protein 5	4.159	12.816	6.463	1.794
Q8IUW5	RELL1	RELT-like protein 1	-	-	31.204	2266.783
Q8WZ73	RFFL	E3 ubiquitin-protein ligase rififylin	3.056	1.607	-	70.139
Q14699	RFTN1	Raftlin	3.792	2.561	9.400	5.896
P22670	RFX1	MHC class II regulatory factor RFX1	38.538	32.145	23.309	18.126
Q9Y3C5	RNF11	RING finger protein 11	4.858	86.569	-	3.240
P10301	RRAS	Ras-related protein R-Ras	10.564	72.650	15.186	6.791
P62070	RRAS2	Ras-related protein R-Ras2	5.304	21.033	9.208	4.193
Q9NQC3	RTN4	Reticulon-4	0.985	3.652	29.443	-
O95977	S1PR4	Sphingosine 1-phosphate receptor 4	17.565	16.749	11.729	10.976
O15126	SCAMP1	Secretory carrier-associated membrane protein 1	6168.301	9.754	8.955	2.104
O15127	SCAMP2	Secretory carrier-associated membrane protein 2	7.123	553787.397	34.072	16.556
O14828	SCAMP3	Secretory carrier-associated membrane protein 3	17.198	24.507	9.436	10.253
Q969E2	SCAMP4	Secretory carrier-associated membrane protein 4	-	6.431	4.282	5.013
Q14108	SCARB2	Lysosome membrane protein 2	17.279	5.001	15.429	4.101
Q14160	SCRIB	Protein scribble homolog	99.755	97.878	313.996	4.225
Q9H190	SDCBP2	Syntenin-2	-	13.696	4.043	-
Q92854	SEMA4D	Semaphorin-4D	7.291	3.690	3.156	1.812
Q9NVA2	SEPT11	Septin-11	4.394	-	0.436	3.135
Q9NRX5	SERINC1	Serine incorporator 1	11928.105	2821.982	9.251	6.757
Q8NBJ9	SIDT2	SID1 transmembrane family member 2	-	2312.752	2512.116	193548.387
Q6IA17	SIGIRR	Single Ig IL-1-related receptor	17.067	28.687	108.391	11.404
Q8N697	SLC15A4	Solute carrier family 15 member 4	15.245	8.513	-	0.519
O15427	SLC16A3	Monocarboxylate transporter 4 (Fragment)	17.206	207.150	1.115	74803.836
Q15758	SLC1A5	Neutral amino acid transporter B(0)	21.650	21.918	-	0.579
Q6NXT4	SLC30A6	Zinc transporter 6	4.221	-	-	3.037
Q8TB61	SLC35B2	Adenosine 3'-phospho 5'-phosphosulfate transporter 1	665.278	9.304	6.029	1.265
Q96K37	SLC35E1	Solute carrier family 35 member E1	-	8.517	4.188	1.384
P0CK96	SLC35E2B	Solute carrier family 35 member E2B	2.208	23.806	10.210	9.747
Q96GZ6	SLC41A3	Solute carrier family 41 member 3	35130.719	365.372	14.998	-
Q8WWI5	SLC44A1	Choline transporter-like protein 1	48.611	135.901	3.703	4.678
Q8IWA5	SLC44A2	Choline transporter-like protein 2	7.936	13.081	8.130	3.221
Q92581-2	SLC9A6	Isoform 2 of Sodium/hydrogen exchanger 6	94.853	33.609	-	-
Q8IVB4	SLC9A9	Sodium/hydrogen exchanger 9	3.144	6.238	-	74.045
Q9UIG8	SLCO3A1	Solute carrier organic anion transporter family member 3A1	112.337	9.221	8.065	8.401
B2RUZ4	SMIM1	Small integral membrane protein 1	5.625	24.655	-	-
Q9NY59	SMPD3	Sphingomyelin phosphodiesterase 3	-	-	206.257	8.690
O00161	SNAP23	Synaptosomal-associated protein 23	3.945	24.507	3.678	1.500
Q99523	SORT1	Sortilin	12.312	-	-	5.745
Q8TCT8	SPPL2A	Signal peptide peptidase-like 2A	16.160	16.979	-	47.193
Q9NUV7	SPTLC3	Serine palmitoyltransferase 3 (Fragment)	-	-	6.618	201.918
O95772	STARD3NL	MLN64 N-terminal domain homolog	12.361	250.371	-	10.113
O75716	STK16	Serine/threonine-protein kinase 16	1478.758	108.254	7.595	11.526
P27105	STOM	Erythrocyte band 7 integral membrane protein	165.278	1044.133	8.759	22.816
O75558	STX11	Syntaxin-11	12.075	53.236	8.065	5.073
Q86Y82	STX12	Syntaxin-12	14.322	109.209	-	-
O43752	STX6	Syntaxin-6	214869.281	44.367	-	3.331
O15400	STX7	Syntaxin-7	49.747	14.613	-	1.539
Q9UNK0	STX8	Syntaxin-8	7.025	205.389	3.001	0.004
O15260	SURF4	Surfeit locus protein 4	20.278	10.004	-	4.028
Q96L08	SUSD3	Sushi domain-containing protein 3	16.585	69.361	-	-
Q6ZMZ3	SYNE3	Nesprin-3	8.374	8.491	2.970	-
Q6NXT6	TAPT1	Transmembrane anterior posterior transformation protein 1 homolog	3.248	24.273	-	1.681
Q96BS2	TESC	Calcineurin B homologous protein 3	-	14.348	7.617	4.621
P02786	TFRC	Transferrin receptor protein 1	39.444	6.556	3.054	3.011
Q8WUY1	THEM6	Protein THEM6	-	3.624	-	4.339
Q99805	TM9SF2	Transmembrane 9 superfamily member 2	8.391	8.856	-	0.105
Q7Z403	TMC6	Transmembrane channel-like protein 6	12.516	25.992	8.995	0.244
Q8IU68	TMC8	Transmembrane channel-like protein 8	45.931	26.374	6.391	3.324
Q13445	TMED1	Transmembrane emp24 domain-containing protein 1	18.096	73.923	5.367	4.681
Q9H6X4	TMEM134	Transmembrane protein 134	1.064	16.389	8.705	4.991
Q7Z7N9	TMEM179B	Transmembrane protein 179B	14.967	11.301	-	-
Q8IY95	TMEM192	Transmembrane protein 192	-	3.178	3.732	10.846
Q9H0R3	TMEM222	Transmembrane protein 222	4.748	39.784	23.656	15.057
Q9H330	TMEM245	Transmembrane protein 245	13.750	19.669	2.680	15.327
Q86T03	TMEM55B	Transmembrane protein 55B	3.379	77.742	8.817	5.319
O94886	TMEM63A	Transmembrane protein 63A	10.637	14.564	11.653	12.912
Q8NBN3	TMEM87A	Transmembrane protein 87A	8.116	22.597	-	-
Q96BF3	TMIGD2	Transmembrane and immunoglobulin domain-containing protein 2	7.117	-	-	15.580
Q6ZT21	TMPPE	Transmembrane protein with metallophosphoesterase domain	36.765	46.552	6.459	3.385
Q9H3N1	TMX1	Thioredoxin-related transmembrane protein 1	31.585	43.178	1.646	13.112
Q96JJ7	TMX3	Protein disulfide-isomerase TMX3	11.095	95.353	-	8.797
Q9H1E5	TMX4	Thioredoxin-related transmembrane protein 4	16.716	18.027	19.533	9.547
O43557	TNFSF14	Tumor necrosis factor ligand superfamily member 14	3.246	21.727	6.058	5.359
Q9Y228	TRAF3IP3	TRAF3-interacting JNK-activating modulator	10.376	40.632	3.675	1.888
Q86YW5	TREML1	Trem-like transcript 1 protein	10.539	4.572	-	-
P49815	TSC2	Tuberin	-	-	9.013	13.662
Q96SJ8	TSPAN18	Tetraspanin-18 (Fragment)	-	-	7.443	64.455
Q86UF1	TSPAN33	Tetraspanin-33	4.571	23.149	22.278	12.214
O75954	TSPAN9	Tetraspanin-9	-	20.059	-	3.799
Q9C0H2	TTYH3	Protein tweety homolog 3	10.621	24.867	-	0.653
O75385	ULK1	Serine/threonine-protein kinase ULK1	-	925100.785	6.691	1482.127
P63027	VAMP2	Vesicle-associated membrane protein 2	15.139	2.028	-	4.365
Q15836	VAMP3	Vesicle-associated membrane protein 3	6.937	151.538	-	-
O75379	VAMP4	Vesicle-associated membrane protein 4	13.039	10.586	-	1.520
Q8IUH5	ZDHHC17	Palmitoyltransferase ZDHHC17	-	486.314	-	5.846
Q9NUE0	ZDHHC18	Palmitoyltransferase ZDHHC18	5.221	630171.865	273.743	13.662
Q5W0Z9	ZDHHC20	Probable palmitoyltransferase ZDHHC20	13.603	5.752	4.116	9.468
Q9C0B5	ZDHHC5	Palmitoyltransferase ZDHHC5	7.673	22.321	5.454	0.930

Supplementary Table 2

The T-cell palmitome, proteins identified as palmitoylated in our study and/or two previous studies in Jurkat T cells. Proteins identified as palmitoylated only in our study are highlighted in blue. Proteins identified as palmitoylated in both previous studies, but not our study, are highlighted in yellow. [a]Palmitoylation sites predicted using CSS-Palm (version 3.0), with a “High” threshold and a cutoff score of 1.0. [b]Prediction of transmembrane domains was performed using TMHMM Server (version 2.0). [c]References: [1] Martin and Cravatt, Nat. Meth., 2009, [2] Dowal et al., Blood, 2011, [3] Merrick et al., Mol. & Cell. Proteom., 2011, [4] Yang et al., Mol. & Cell. Proteom., 2010, [5] Marin et al., Circ. Res., 2012, [6] Forrester et al., J. Lip. Res., 2011, [7] Wilson et al., Mol. & Cell. Proteom., 2011, [8] Kang et al., Nature, 2008.

UniProt Accession Number	Gene Name	Protein	Enriched in Jurkat T cells	Enriched in Primary T cells	Enriched in Martin et al. 2009	Enriched in Wilson et al. 2011	Predicted Palmitoylation Sites[a]	Predicted Transmembrane Helices[b]	Literature References[c]
Q9NP58	ABCB6	ATP-binding cassette sub-family B member 6, mitochondrial	+	-	-	-	0	9	[2]
O15439	ABCC4	Multidrug resistance-associated protein 4	-	+	-	-	2	11	[2]
P28288	ABCD3	ATP-binding cassette sub-family D member 3	-	-	+	-	0	3	[1]
O95870	ABHD16A	Abhydrolase domain-containing protein 16A	-	+	+	-	1	2	[1], [8]
Q96GS6	ABHD17A	Alpha/beta hydrolase domain-containing protein 17A	+	+	+	-	4	0	[1]
Q5VST6	ABHD17B	Alpha/beta hydrolase domain-containing protein 17B	+	+	+	+	4	0	[1], [3], [7]
Q6PCB6	ABHD17C	Alpha/beta hydrolase domain-containing protein 17C	-	-	+	-	1	0	[1]
P09110	ACAA1	3-ketoacyl-CoA thiolase, peroxisomal	+	+	-	-	0	0	-
Q9H845	ACAD9	Acyl-CoA dehydrogenase family member 9, mitochondrial	-	-	+	-	2	0	[1]
P24752	ACAT1	Acetyl-CoA acetyltransferase, mitochondrial	+	+	-	-	0	0	-
Q9BWD1	ACAT2	Acetyl-CoA acetyltransferase, cytosolic	+	+	-	-	2	0	-
O14734	ACOT8	Acyl-coenzyme A thioesterase 8	-	+	-	-	0	0	-
P33121	ACSL1	Long-chain-fatty-acid--CoA ligase 1	-	+	-	-	0	1	-
O95573	ACSL3	Long-chain-fatty-acid--CoA ligase 3	-	-	+	-	0	1	[1]
P68133	ACTA1	Actin, alpha skeletal muscle	-	-	+	+	1	0	[1], [7]
P62736	ACTA2	Actin, aortic smooth muscle	-	-	+	-	2	0	[5]
P60709	ACTB	Actin, cytoplasmic 1	-	-	-	+	1	0	[4], [5], [7]
P68032	ACTC1	Actin, alpha cardiac muscle 1	-	-	+	-	1	0	[1]
P63267	ACTG2	Actin, gamma-enteric smooth muscle	-	-	+	-	2	0	[5]
O43707	ACTN4	Alpha-actinin-4	-	+	-	-	0	0	[4], [5]
O14672	ADAM10	Disintegrin and metalloproteinase domain-containing protein 10	-	+	+	-	2	1	[1], [8]
P78536	ADAM17	Disintegrin and metalloproteinase domain-containing protein 17	+	+	-	-	4	1	[3]
O43306	ADCY6	Adenylate cyclase type 6	-	+	-	-	0	12	[2], [8]
P51828	ADCY7	Adenylate cyclase type 7	+	+	-	-	4	12	-
Q99943	AGPAT1	1-acyl-sn-glycerol-3-phosphate acyltransferase alpha	+	-	+	-	0	3	[1], [3], [8]
Q9NUQ2	AGPAT5	1-acyl-sn-glycerol-3-phosphate acyltransferase epsilon	-	+	-	-	1	3	-
P43353	ALDH3B1	Aldehyde dehydrogenase family 3 member B1	-	+	-	-	2	0	[3]
Q02252	ALDH6A1	Methylmalonate-semialdehyde dehydrogenase [acylating], mitochondrial	+	+	-	-	1	1	-
P04075	ALDOA	Fructose-bisphosphate aldolase A	-	-	+	-	0	0	[1], [6]
Q86YT9	AMICA1	Junctional adhesion molecule-like	-	+	-	-	2	1	-
Q4KMQ2	ANO6	Anoctamin-6	-	-	+	-	1	8	[1]
Q9H6X2	ANTXR1	Anthrax toxin receptor 1	+	-	+	-	4	1	[1]
P58335	ANTXR2	Anthrax toxin receptor 2	-	+	-	-	5	1	[5]
P08133	ANXA6	Annexin A6	-	-	-	+	1	0	[7]
Q63HQ0	AP1AR	AP-1 complex-associated regulatory protein	+	+	+	-	3	0	[1]
O43747	AP1G1	AP-1 complex subunit gamma-1	-	-	+	-	0	0	[1]
Q9BZZ5	API5	Apoptosis inhibitor 5	-	-	+	-	0	0	[5]
P84077	ARF1	ADP-ribosylation factor 1	-	-	+	+	0	0	[1], [7]
P61204	ARF3	ADP-ribosylation factor 3	-	-	+	-	0	0	[1]
P18085	ARF4	ADP-ribosylation factor 4	-	-	-	+	0	0	[5], [7], [8]
P84085	ARF5	ADP-ribosylation factor 5	-	-	-	+	0	0	[2], [7], [8]
Q3SXY8	ARL13B	ADP-ribosylation factor-like protein 13B	-	-	+	-	2	0	[1]
Q9NXU5	ARL15	ADP-ribosylation factor-like protein 15	+	+	+	-	0	0	[1], [2], [3], [8]
Q8N6S5	ARL6IP6	ADP-ribosylation factor-like protein 6-interacting protein 6	+	+	+	-	1	3	[1]
P98196	ATP11A	Probable phospholipid-transporting ATPase IH	-	+	-	-	3	7	[4]
Q9Y2G3	ATP11B	Probable phospholipid-transporting ATPase IF	+	+	+	-	4	8	[1], [2]
Q8NB49	ATP11C	Probable phospholipid-transporting ATPase IG	-	+	-	-	0	7	-
Q9H7F0	ATP13A3	Probable cation-transporting ATPase 13A3	-	-	+	-	2	10	[1]
P05023	ATP1A1	Sodium/potassium-transporting ATPase subunit alpha-1	-	-	+	-	0	10	[1], [2], [4], [6]
P13637	ATP1A3	Sodium/potassium-transporting ATPase subunit alpha-3	-	-	+	-	0	8	[1]
Q93084	ATP2A3	Sarcoplasmic/endoplasmic reticulum calcium ATPase 3	-	-	+	-	1	7	[1]
P23634	ATP2B4	Plasma membrane calcium-transporting ATPase 4	-	-	+	-	0	8	[1]
P25705	ATP5A1	ATP synthase subunit alpha, mitochondrial	-	-	-	+	0	0	[5], [7]
P36542	ATP5C1	ATP synthase subunit gamma, mitochondrial	-	-	+	-	0	0	[1], [6]
P48047	ATP5O	ATP synthase subunit O, mitochondrial	-	-	+	-	0	0	[1], [8]
Q9Y487	ATP6V0A2	V-type proton ATPase 116 kDa subunit a isoform 2	+	+	+	-	1	6	[1], [2]
O43861	ATP9B	Probable phospholipid-transporting ATPase IIB	+	-	+	-	1	0	[1]
O75882	ATRN	Attractin	+	-	-	-	4	1	[8]
Q9Y679	AUP1	Ancient ubiquitous protein 1	+	+	+	-	1	2	[1]
Q9Y679-3	AUP1	Ancient ubiquitous protein 1 (Isoform 3)	+	-	-	-	1	0	-
O43505	B3GNT1	N-acetyllactosaminide beta-1,3-N-acetylglucosaminyltransferase	+	-	+	-	1	1	[1]
P15291	B4GALT1	Beta-1,4-galactosyltransferase 1	+	+	+	-	1	1	[1]
Q9UBV7	B4GALT7	Beta-1,4-galactosyltransferase 7	+	+	-	-	1	1	-
Q9HB09	BCL2L12	Bcl-2-like protein 12	+	-	-	-	0	0	-
O15155	BET1	BET1 homolog	-	+	-	-	0	1	[2]
Q9NYM9	BET1L	BET1-like protein	+	-	-	-	0	1	[2]
Q10589	BST2	Bone marrow stromal antigen 2	+	+	-	-	2	1	-
Q9Y6E2	BZW2	Basic leucine zipper and W2 domain-containing protein 2	-	-	+	-	0	0	[1]
Q07021	C1QBP	Complement component 1 Q subcomponent-binding protein, mitochondrial	-	-	-	+	0	0	[2], [5], [7]
Q8N357	C2orf18	Solute carrier family 35 member F6	-	+	-	-	0	10	-
Q6P1X6	C8orf82	UPF0598 protein C8orf82	+	-	-	-	1	0	[2]
Q9HA72	CALHM2	Calcium homeostasis modulator protein 2	-	+	-	-	1	4	-
P27824	CANX	Calnexin	+	+	+	+	2	1	[1], [3], [5], [7], [8]
O15484	CAPN5	Calpain-5	-	+	-	-	1	0	[4], [5], [8]
Q14444	CAPRIN1	Caprin-1	-	-	+	-	0	0	[1]
P83916	CBX1	Chromobox protein homolog 1	-	-	+	-	0	0	[1]
Q13185	CBX3	Chromobox protein homolog 3	-	-	+	-	1	0	[1]
A6NI79	CCDC69	Coiled-coil domain-containing protein 69	-	+	-	-	1	0	-
Q8ND76	CCNY	Cyclin-Y	+	+	+	-	2	0	[1], [2], [3]
Q8ND76-3	CCNY	Cyclin-Y (Isoform 3)	+	-	-	-	0	0	-
Q8N7R7	CCNYL1	Cyclin-Y-like protein 1	+	-	+	-	2	0	[1]
P51681	CCR5	C-C chemokine receptor type 5	-	+	-	-	4	6	-
P78371	CCT2	T-complex protein 1 subunit beta	-	-	+	+	1	0	[1], [7]
P49368	CCT3	T-complex protein 1 subunit gamma	-	-	-	+	0	0	[7]
P50991	CCT4	T-complex protein 1 subunit delta	-	-	-	+	0	0	[7]
P48643	CCT5	T-complex protein 1 subunit epsilon	-	-	+	+	0	0	[1], [5], [7]
P40227	CCT6A	T-complex protein 1 subunit zeta	-	-	-	+	0	0	[5], [7]
P50990	CCT8	T-complex protein 1 subunit theta	-	-	-	+	1	0	[5], [7]
P48509	CD151	CD151 antigen	-	+	-	-	7	4	[2], [6]
P06126	CD1A	T-cell surface glycoprotein CD1a	-	-	+	-	2	0	[1]
P29017	CD1C	T-cell surface glycoprotein CD1c	+	-	-	-	1	1	-
Q8TD46	CD200R1	Cell surface glycoprotein CD200 receptor 1	-	+	-	-	1	2	-
P26842	CD27	CD27 antigen	-	+	-	-	1	1	-
P16671	CD36	Platelet glycoprotein 4 (Fragment)	-	+	-	-	4	2	[2], [3]
P11049	CD37	Leukocyte antigen CD37	-	+	-	-	4	4	-
P28907	CD38	ADP-ribosyl cyclase 1	+	+	+	-	2	1	[1], [2], [4], [8]
P04234	CD3D	T-cell surface glycoprotein CD3 delta chain	+	+	+	-	0	0	[1]
P01730	CD4	T-cell surface glycoprotein CD4	-	+	+	-	3	1	[1]
P16070	CD44	CD44 antigen (Fragment)	-	+	-	-	2	1	[3], [5]
P06127	CD5	T-cell surface glycoprotein CD5	+	-	+	-	2	1	[1]
P19397	CD53	Leukocyte surface antigen CD53	+	+	-	-	4	4	-
P08962	CD63	CD63 antigen	+	+	-	-	7	4	[2], [5]
P09564	CD7	T-cell antigen CD7	+	+	-	-	2	1	-
P21854	CD72	B-cell differentiation antigen CD72	-	+	-	-	2	1	-
P27701	CD82	CD82 antigen	+	+	+	+	5	4	[1], [2], [5], [7]
Q01151	CD83	CD83 antigen	-	+	-	-	0	1	-
P21926	CD9	CD9 antigen	-	+	-	-	6	4	[5]
P40200	CD96	T-cell surface protein tactile	-	+	-	-	1	1	-
P48960-2	CD97	CD97 antigen (Isoform 2)	+	-	-	-	0	7	-
P48960	CD97	CD97 antigen	-	+	-	-	0	7	[4]
P14209	CD99	CD99 antigen	+	+	-	-	0	2	[5], [6]
Q8TCZ2	CD99L2	CD99 antigen-like protein 2	-	+	-	-	1	1	[2], [8]
Q9NRR3	CDC42SE2	CDC42 small effector protein 2	-	-	+	-	3	0	[1], [2]

UniProt Accession Number	Gene Name	Protein	Enriched in Jurkat T cells	Enriched in Primary T cells	Enriched in Martin et al. 2009	Enriched in Wilson et al. 2011	Predicted Palmitoylation Sites[a]	Predicted Transmembrane Helices[b]	Literature References[c]
Q5VV42	CDKAL1	Threonylcarbamoyladenosine tRNA methylthiotransferase	-	+	-	-	1	1	-
Q96G23	CERS2	Ceramide synthase 2	-	-	+	-	0	5	[1]
P23528	CFL1	Cofilin-1	-	-	-	+	0	0	[5], [7]
Q14839	CHD4	Chromodomain-helicase-DNA-binding protein 4	-	-	+	-	0	0	[1]
Q9NPF2	CHST11	Carbohydrate sulfotransferase 11	+	-	+	-	1	1	[1]
Q07065	CKAP4	Cytoskeleton-associated protein 4	+	+	-	-	1	1	[3], [4], [5]
Q9NY35	CLDND1	Claudin domain-containing protein 1	-	+	-	-	2	4	-
O14967	CLGN	Calmegin	+	-	+	-	1	1	[1]
O00299	CLIC1	Chloride intracellular channel protein 1	-	-	-	+	0	0	[7]
Q96DZ5	CLIP3	CAP-Gly domain-containing linker protein 3	+	-	-	-	2	0	[8]
Q00610	CLTC	Clathrin heavy chain 1	-	-	+	+	0	0	[1], [7]
A5YKK6	CNOT1	CCR4-NOT transcription complex subunit 1	-	-	+	-	3	0	[1]
Q6PJW8	CNST	Consortin	+	+	-	-	2	1	-
Q86VU5	COMTD1	Catechol O-methyltransferase domain-containing protein 1 (Fragment)	-	+	-	-	0	1	[2]
P53621	COPA	Coatomer subunit alpha	-	-	+	-	0	0	[1]
P31146	CORO1A	Coronin-1A	-	-	-	+	0	0	[7]
Q9UI42	CPA4	Carboxypeptidase A4	+	-	-	-	0	0	-
O75976	CPD	Carboxypeptidase D	-	-	+	-	3	1	[1], [8]
P50416	CPT1A	Carnitine O-palmitoyltransferase 1, liver isoform	-	-	+	-	0	2	[1]
P55060	CSE1L	Exportin-2	-	-	+	-	0	0	[1]
Q9HCP0	CSNK1G1	Casein kinase I isoform gamma-1	+	+	+	-	3	0	[1], [2], [8]
P78368	CSNK1G2	Casein kinase I isoform gamma-2	+	+	-	-	3	0	[8]
Q9Y6M4	CSNK1G3	Casein kinase I isoform gamma-3	-	+	+	-	3	0	[1], [2], [8]
Q9Y6M4-3	CSNK1G3	Casein kinase I isoform gamma-3 (Isoform 3)	+	-	-	-	3	0	-
Q9GZU7	CTDSP1	Carboxy-terminal domain RNA polymerase II polypeptide A small phosphatase 1	-	+	+	-	0	0	[1]
Q9GZU7-2	CTDSP1	Carboxy-terminal domain RNA polymerase II polypeptide A small phosphatase 1	+	+	-	-	0	0	-
O14595	CTDSP2	Carboxy-terminal domain RNA polymerase II polypeptide A small phosphatase 2	-	+	-	-	0	0	-
P78310-2	CXADR	Coxsackievirus and adenovirus receptor	+	-	-	-	2	1	-
P61073	CXCR4	C-X-C chemokine receptor type 4	-	-	+	-	1	7	[1]
O43169	CYB5B	Cytochrome b5 type B	-	-	-	+	0	1	[2], [5], [7], [8]
P00387	CYB5R3	NADH-cytochrome b5 reductase 3	-	-	+	-	0	0	[1], [8]
P04839	CYBB	Cytochrome b-245 heavy chain	-	+	-	-	2	4	[3]
Q9Y4D1	DAAM1	Disheveled-associated activator of morphogenesis 1	+	+	+	-	1	0	[1], [2], [8]
P61803	DAD1	Dolichyl-diphosphooligosaccharide--protein glycosyltransferase subunit DAD1	-	-	+	-	0	3	[1]
Q8NCG7	DAGLB	Sn1-specific diacylglycerol lipase beta	+	+	+	-	3	4	[1], [2], [3]
Q8IWE4	DCUN1D3	DCN1-like protein 3	+	+	-	-	2	0	-
Q16531	DDB1	DNA damage-binding protein 1	-	-	-	+	0	0	[7]
P17844	DDX5	Probable ATP-dependent RNA helicase DDX5	-	-	-	+	0	0	[7]
Q9BSY9	DESI2	Desumoylating isopeptidase 2	+	-	-	-	0	0	-
Q9P202	DFNB31	Whirlin	-	+	-	-	0	0	-
P52429	DGKE	Diacylglycerol kinase epsilon	-	+	-	-	2	1	-
O43143	DHX15	Putative pre-mRNA-splicing factor ATP-dependent RNA helicase DHX15	-	-	-	+	0	0	[7]
Q08211	DHX9	ATP-dependent RNA helicase A	-	-	-	+	1	0	[4], [7]
Q9Y2H0	DLGAP4	Disks large-associated protein 4	+	-	-	-	2	0	[2]
P31689	DNAJA1	DnaJ homolog subfamily A member 1	-	-	+	-	1	0	[1]
O75190-3	DNAJB6	DnaJ homolog subfamily B member 6	-	+	-	-	0	0	-
Q9NVH1	DNAJC11	DnaJ homolog subfamily C member 11	-	-	+	-	0	0	[1], [8]
Q9H3Z4	DNAJC5	DnaJ homolog subfamily C member 5	+	+	+	-	13	1	[1], [2], [3]
Q14126	DSG2	Desmoglein-2	+	-	+	-	1	2	[1]
Q14204	DYNC1H1	Cytoplasmic dynein 1 heavy chain 1	-	-	+	-	0	0	[1]
O00559	EBAG9	Receptor-binding cancer antigen expressed on SiSo cells	+	-	+	-	1	0	[1], [2], [8]
P42892-3	ECE1	Endothelin-converting enzyme 1 (Isoform 3)	+	-	-	-	0	1	-
P42892	ECE1	Endothelin-converting enzyme 1	+	+	-	-	0	1	[2], [5]
Q13011	ECH1	Delta(3,5)-Delta(2,4)-dienoyl-CoA isomerase, mitochondrial	-	-	-	+	0	0	[7]
Q6P2E9	EDC4	Enhancer of mRNA-decapping protein 4	-	-	+	-	2	0	[1]
P68104	EEF1A1	Elongation factor 1-alpha 1	-	-	-	+	0	0	[7], [8]
P29692	EEF1D	Elongation factor 1-delta	-	-	+	-	0	0	[1]
P26641	EEF1G	Elongation factor 1-gamma	-	-	+	-	0	0	[1], [6], [8]
P13639	EEF2	Elongation factor 2	-	-	+	+	1	0	[1], [5], [7]
O75071	EFCAB14	EF-hand calcium-binding domain-containing protein 14	+	-	+	-	2	1	[1]
Q14156	EFR3A	Protein EFR3 homolog A	-	+	-	-	4	0	[2], [3]
Q15029	EFTUD2	116 kDa U5 small nuclear ribonucleoprotein component	-	-	-	+	0	0	[7]
Q14152	EIF3A	Eukaryotic translation initiation factor 3 subunit A	-	-	+	-	0	0	[1]
P55884	EIF3B	Eukaryotic translation initiation factor 3 subunit B	-	-	+	-	0	0	[1]
Q99613	EIF3C	Eukaryotic translation initiation factor 3 subunit C	-	-	+	-	0	0	[1]
B5ME19	EIF3CL	Eukaryotic translation initiation factor 3 subunit C-like protein	-	-	+	-	0	0	[5]
P60228	EIF3E	Eukaryotic translation initiation factor 3 subunit E	-	-	+	-	0	0	[1]
Q9Y262	EIF3L	Eukaryotic translation initiation factor 3 subunit L	-	-	+	-	0	0	[1]
Q7L2H7	EIF3M	Eukaryotic translation initiation factor 3 subunit M	-	+	-	-	2	0	-
P38919	EIF4A3	Eukaryotic initiation factor 4A-III	-	-	+	-	0	0	[1]
P63241	EIF5A	Eukaryotic translation initiation factor 5A-1	-	-	+	-	0	0	[1], [2], [6]
Q9NYP7	ELOVL5	Elongation of very long chain fatty acids protein 5	-	+	-	-	1	7	-
Q6PCB8	EMB	Embigin	+	-	-	-	0	1	-
P54852	EMP3	Epithelial membrane protein 3 (Fragment)	-	+	-	-	1	4	-
P06733	ENO1	Alpha-enolase	-	-	-	+	0	0	[5], [7]
Q9Y227	ENTPD4	Ectonucleoside triphosphate diphosphohydrolase 4	-	+	-	-	0	2	-
P07814	EPRS	Bifunctional glutamate/proline--tRNA ligase	-	-	+	-	1	0	[1], [8]
Q96RT1	ERBB2IP	Protein LAP2	-	+	+	-	3	0	[1], [2]
Q96RT1-7	ERBB2IP	Protein LAP2 (Isoform 7)	+	-	-	-	3	0	-
Q969X5	ERGIC1	Endoplasmic reticulum-Golgi intermediate compartment protein 1	+	+	-	-	1	2	[3]
Q96RQ1	ERGIC2	Endoplasmic reticulum-Golgi intermediate compartment protein 2	-	+	+	-	0	0	[1]
Q9Y282	ERGIC3	Endoplasmic reticulum-Golgi intermediate compartment protein 3	+	+	+	-	0	2	[1], [2], [3], [5], [8]
Q9BSJ8	ESYT1	Extended synaptotagmin-1	-	-	+	-	0	2	[1], [6]
A0FGR8	ESYT2	Extended synaptotagmin-2	-	-	+	-	0	1	[1]
P34910	EVI2B	Protein EVI2B	-	+	-	-	1	1	-
Q92817	EVPL	Envoplakin	-	+	-	-	2	0	-
P05413	FABP3	Fatty acid-binding protein, heart	+	-	-	-	1	0	-
A8MVW0	FAM171A2	Protein FAM171A2	+	-	-	-	2	2	-
Q8N128	FAM177A1	Protein FAM177A1	+	+	-	-	0	0	-
Q8IW50	FAM219A	Protein FAM219A	-	-	+	-	2	0	[5]
Q9NUQ9	FAM49B	Protein FAM49B	+	+	+	-	0	0	[1], [2]
P25445	FAS	Tumor necrosis factor receptor superfamily member 6	+	+	-	-	2	0	-
Q9Y3I1	FBXO7	F-box only protein 7	-	+	-	-	0	0	-
P02671	FGA	Fibrinogen alpha chain	+	-	-	-	1	0	-
P02675	FGB	Fibrinogen beta chain	+	-	-	-	0	0	-
P09769	FGR	Tyrosine-protein kinase Fgr	-	+	-	-	2	0	-
Q14318	FKBP8	Peptidyl-prolyl cis-trans isomerase FKBP8	-	-	+	-	1	1	[1]
P21333	FLNA	Filamin-A	-	-	+	-	0	0	[1], [4]
O75369	FLNB	Filamin-B	-	-	+	-	0	0	[1], [4], [5]
O75955	FLOT1	Flotillin-1	+	+	+	-	1	0	[1], [2], [3], [4], [5]
Q14254	FLOT2	Flotillin-2	+	+	+	-	3	0	[1], [2], [3], [5]
Q9Y2H6	FNDC3A	Fibronectin type-III domain-containing protein 3A	-	+	-	-	1	1	[8]
P06241	FYN	Tyrosine-protein kinase Fyn	-	+	+	-	4	0	[1], [2], [3], [5]
P06241-3	FYN	Tyrosine-protein kinase Fyn (Isoform 3)	+	-	-	-	2	0	-
Q10472	GALNT1	Polypeptide N-acetylgalactosaminyltransferase 1	+	+	-	-	1	1	-
Q8IXK2	GALNT12	Polypeptide N-acetylgalactosaminyltransferase 12	+	-	+	-	1	1	[1]
Q14697	GANAB	Neutral alpha-glucosidase AB	-	-	-	+	0	1	[7]
P04406	GAPDH	Glyceraldehyde-3-phosphate dehydrogenase	-	-	-	+	1	0	[4], [6], [7]
Q9NY12	GAR1	H/ACA ribonucleoprotein complex subunit 1	+	-	-	-	0	0	-
P50395	GDI2	Rab GDP dissociation inhibitor beta	-	-	-	+	0	0	[7]
Q9UG22	GIMAP2	GTPase IMAP family member 2	-	+	-	-	3	2	-
Q92896	GLG1	Golgi apparatus protein 1	-	-	+	-	2	1	[1], [3], [8]
P29992	GNA11	Guanine nucleotide-binding protein subunit alpha-11	-	+	+	-	2	0	[1], [2], [4], [5], [6]
Q03113	GNA12	Guanine nucleotide-binding protein subunit alpha-12	-	+	-	-	0	0	[4]
Q14344	GNA13	Guanine nucleotide-binding protein subunit alpha-13	+	+	+	-	2	0	[1], [2], [4], [5]
P30679	GNA15	Guanine nucleotide-binding protein subunit alpha-15	+	-	+	-	3	0	[1], [2]
P04899	GNAI2	Guanine nucleotide-binding protein G(i) subunit alpha-2	-	-	+	-	1	0	[1], [4], [5]
P08754	GNAI3	Guanine nucleotide-binding protein G(k) subunit alpha	-	-	+	-	2	0	[1], [2], [4], [5]
P09471	GNAO1	Guanine nucleotide-binding protein G(o) subunit alpha	-	-	+	-	1	0	[5]
P09471-2	GNAO1	Isoform Alpha-2 of Guanine nucleotide-binding protein G(o) subunit alpha	-	+	-	-	1	0	-

UniProt Accession Number	Gene Name	Protein	Enriched in Jurkat T cells	Enriched in Primary T cells	Enriched in Martin et al. 2009	Enriched in Wilson et al. 2011	Predicted Palmitoylation Sites[a]	Predicted Transmembrane Helices[b]	Literature References[c]
P50148	GNAQ	Guanine nucleotide-binding protein G(q) subunit alpha	+	+	+	-	2	0	[1], [2], [3], [4], [5]
P84996	GNAS	Protein ALEX	-	-	+	-	4	0	[4], [5]
P63092	GNAS	Guanine nucleotide-binding protein G(s) subunit alpha isoforms short	-	+	-	-	1	0	[6]
P19086	GNAZ	Guanine nucleotide-binding protein G(z) subunit alpha	-	+	-	-	2	0	-
P63244	GNB2L1	Guanine nucleotide-binding protein subunit beta-2-like 1	-	-	-	+	0	0	[5], [7]
O15228	GNPAT	Dihydroxyacetone phosphate acyltransferase	-	-	+	-	1	0	[1]
Q7Z5G4	GOLGA7	Golgin subfamily A member 7	+	+	+	-	0	0	[1], [2], [3]
Q2TAP0	GOLGA7B	Golgin subfamily A member 7B	-	+	-	-	0	0	-
Q14789	GOLGB1	Golgin subfamily B member 1	+	+	+	-	2	1	[1]
Q99795	GPA33	Cell surface A33 antigen	-	+	-	-	6	1	-
Q9NPR9	GPR108	Protein GPR108	-	+	-	-	0	7	-
P32249	GPR183	G-protein coupled receptor 183	-	+	-	-	0	7	-
Q15743	GPR68	Ovarian cancer G-protein coupled receptor 1	-	+	-	-	1	7	-
Q6ZVF9	GPRIN3	G protein-regulated inducer of neurite outgrowth 3	-	+	-	-	1	0	[8]
P07203	GPX1	Glutathione peroxidase 1	+	-	-	-	2	0	[8]
P09211	GSTP1	Glutathione S-transferase P	-	-	+	+	0	0	[1], [7]
Q8WW33	GTSF1	Gametocyte-specific factor 1	+	-	-	-	0	0	-
O75367	H2AFY	Core histone macro-H2A.1	-	-	+	-	0	0	[5]
P84243	H3F3B	Histone H3	-	-	-	+	0	0	[7]
P40939	HADHA	Trifunctional enzyme subunit alpha, mitochondrial	-	-	+	-	1	0	[1], [8]
P55084	HADHB	Trifunctional enzyme subunit beta, mitochondrial	-	-	+	-	0	0	[1]
Q9BSH5	HDHD3	Haloacid dehalogenase-like hydrolase domain-containing protein 3	-	+	-	-	1	0	-
O75146	HIP1R	Huntingtin-interacting protein 1-related protein	-	+	-	-	1	0	-
P30443	HLA-A	HLA class I histocompatibility antigen, A-1 alpha chain	-	-	+	-	1	1	[5]
P01889	HLA-B	HLA class I histocompatibility antigen, B-7 alpha chain	+	-	+	-	2	1	[1], [4]
P30499	HLA-C	HLA class I histocompatibility antigen, Cw-1 alpha chain	-	-	+	-	2	1	[3], [4], [5]
P30504	HLA-C	HLA class I histocompatibility antigen, Cw-4 alpha chain	+	+	-	-	2	1	-
P30501	HLA-C	HLA class I histocompatibility antigen, Cw-2 alpha chain	-	+	-	-	1	1	-
P13747	HLA-E	HLA class I histocompatibility antigen, alpha chain E	-	-	+	-	0	1	[1]
P09429	HMGB1	High mobility group protein B1	-	-	-	+	0	0	[7]
P30519	HMOX2	Heme oxygenase 2	-	-	+	-	0	1	[1]
P09651	HNRNPA1	Heterogeneous nuclear ribonucleoprotein A1	-	-	-	+	0	0	[5], [7], [8]
P22626	HNRNPA2B1	Heterogeneous nuclear ribonucleoproteins A2/B1	-	-	-	+	0	0	[5], [7], [8]
Q14103	HNRNPD	Heterogeneous nuclear ribonucleoprotein D0	-	-	-	+	0	0	[7]
P31942	HNRNPH3	Heterogeneous nuclear ribonucleoprotein H3	-	-	+	-	0	0	[1]
P61978	HNRNPK	Heterogeneous nuclear ribonucleoprotein K	-	-	-	+	0	0	[5], [7]
P14866	HNRNPL	Heterogeneous nuclear ribonucleoprotein L	-	-	+	-	3	0	[1]
Q00839	HNRNPU	Heterogeneous nuclear ribonucleoprotein U	-	-	-	+	0	0	[7], [8]
P00738	HP	Haptoglobin	+	-	-	-	0	0	-
Q5SSJ5	HP1BP3	Heterochromatin protein 1-binding protein 3	-	-	+	-	0	0	[1]
P01112	HRAS	GTPase HRas	+	-	+	-	3	0	[1], [2], [5], [6]
Q99714	HSD17B10	3-hydroxyacyl-CoA dehydrogenase type-2	-	-	+	+	1	0	[1], [7]
P07900	HSP90AA1	Heat shock protein HSP 90-alpha	-	-	-	+	2	0	[7]
P08238	HSP90AB1	Heat shock protein HSP 90-beta	-	-	-	+	1	0	[7]
P14625	HSP90B1	Endoplasmin	-	-	-	+	3	0	[7]
P08107	HSPA1A	Heat shock 70 kDa protein 1A/1B	-	-	+	+	0	0	[1], [7]
P34932	HSPA4	Heat shock 70 kDa protein 4	-	-	-	+	2	0	[7]
P11021	HSPA5	78 kDa glucose-regulated protein	-	-	-	+	0	0	[7]
P11142	HSPA8	Heat shock cognate 71 kDa protein	-	-	-	+	1	0	[7]
P38646	HSPA9	Stress-70 protein, mitochondrial	-	-	-	+	0	0	[6], [7]
P10809	HSPD1	60 kDa heat shock protein, mitochondrial	-	-	-	+	0	0	[5], [7]
P41252	IARS	Isoleucine--tRNA ligase, cytoplasmic	-	-	+	-	0	0	[1]
P13598	ICAM2	Intercellular adhesion molecule 2	+	+	-	-	2	1	[5]
Q01629	IFITM2	Interferon-induced transmembrane protein 1	+	-	-	-	3	2	[3], [5]
Q01628	IFITM3	Interferon-induced transmembrane protein 3	-	+	-	-	3	2	[2], [3], [5]
P17181	IFNAR1	Interferon alpha/beta receptor 1	+	-	-	-	1	2	-
P11717	IGF2R	Cation-independent mannose-6-phosphate receptor	+	-	+	+	3	1	[1], [3], [7], [8]
Q969P0	IGSF8	Immunoglobulin superfamily member 8	+	+	+	-	2	1	[1], [8]
P24001	IL32	Interleukin-32	-	-	+	-	5	0	[5]
P24001-4	IL32	Interleukin-32 (Isoform 4)	+	-	-	-	1	0	-
Q71H61	ILDR2	Immunoglobulin-like domain-containing receptor 2	+	-	-	-	12	1	-
Q12905	ILF2	Interleukin enhancer-binding factor 2	-	-	+	-	0	0	[1]
Q16891	IMMT	Mitochondrial inner membrane protein	-	-	+	-	2	0	[1]
P12268	IMPDH2	Inosine-5-monophosphate dehydrogenase 2	-	-	+	-	0	0	[1]
O00410	IPO5	Importin-5	-	-	+	+	2	0	[1], [7], [8]
P46940	IQGAP1	Ras GTPase-activating-like protein IQGAP1	-	-	+	-	0	0	[1]
Q9H0X4	ITFG3	Protein ITFG3	+	+	-	-	2	1	-
P23229-4	ITGA6	Integrin alpha-6 (Isoform 4)	+	-	-	-	2	1	-
P23229	ITGA6	Integrin alpha-6	-	+	-	-	1	1	[5]
O43736	ITM2A	Integral membrane protein 2A	+	+	+	-	1	1	[1]
Q9Y287	ITM2B	Integral membrane protein 2B	+	-	-	-	1	1	-
Q9NQX7	ITM2C	Integral membrane protein 2C	+	-	-	-	2	1	[8]
P23458	JAK1	Tyrosine-protein kinase JAK1	-	+	-	-	3	0	-
Q9BX67	JAM3	Junctional adhesion molecule C	+	+	-	-	3	1	[2], [8]
Q15046	KARS	Lysine--tRNA ligase	-	-	+	-	0	0	[1]
Q9Y4C1	KDM3A	Lysine-specific demethylase 3A	-	+	-	-	0	0	-
Q8IZA0	KIAA0319L	Dyslexia-associated protein KIAA0319-like protein	+	+	-	-	4	1	-
A2VDJ0	KIAA0922	Transmembrane protein 131-like	+	-	-	-	3	0	-
Q9BY89	KIAA1671	Uncharacterized protein KIAA1671	-	+	-	-	0	0	-
Q8IYS2	KIAA2013	Uncharacterized protein KIAA2013	+	+	+	-	0	2	[1], [2], [3]
P52732	KIF11	Kinesin-like protein KIF11	-	-	+	-	2	0	[1]
P43630	KIR3DL2	Killer cell immunoglobulin-like receptor 3DL2	+	-	-	-	1	1	-
Q6UWL6-3	KIRREL2	Kin of IRRE-like protein 2	+	-	-	-	2	1	-
Q8NBE8	KLHL23	Kelch-like protein 23	-	+	-	-	0	0	-
P52292	KPNA2	Importin subunit alpha-2	-	-	+	-	0	0	[1]
Q14974	KPNB1	Importin subunit beta-1	-	-	+	+	2	0	[1], [7]
P01116	KRAS	GTPase KRas	-	-	+	-	2	0	[1]
Q6GTX8	LAIR1	Leukocyte-associated immunoglobulin-like receptor 1	+	-	-	-	1	1	-
Q6IAA8	LAMTOR1	Ragulator complex protein LAMTOR1	+	-	+	+	2	0	[1], [5], [7]
Q9P2J5	LARS	Leucine--tRNA ligase, cytoplasmic	-	-	+	-	0	0	[1]
O43561	LAT	Linker for activation of T-cells family member 1	+	+	+	-	3	1	[1], [2], [3]
P06239	LCK	Tyrosine-protein kinase Lck	+	+	+	-	2	0	[1], [5]
P13796	LCP1	Plastin-2	-	-	-	+	1	0	[7]
P00338	LDHA	L-lactate dehydrogenase A chain	-	-	-	+	1	0	[7]
P07195	LDHB	L-lactate dehydrogenase B chain	-	-	-	+	0	0	[5], [7], [8]
O15165	LDLRAD4	Low-density lipoprotein receptor class A domain-containing protein 4	-	-	+	-	0	1	[5]
O95202	LETM1	LETM1 and EF-hand domain-containing protein 1, mitochondrial	-	-	+	-	1	1	[1]
Q9H400	LIME1	Lck-interacting transmembrane adapter 1	+	+	-	-	3	1	-
Q9BU23	LMF2	Lipase maturation factor 2	-	+	-	-	0	7	-
P20700	LMNB1	Lamin-B1	-	-	-	+	2	0	[7]
Q8IWU2	LMTK2	Serine/threonine-protein kinase LMTK2	+	+	-	-	2	3	-
Q9UIQ6	LNPEP	Leucyl-cystinyl aminopeptidase	-	+	+	-	0	1	[1], [2], [3]
Q9UIQ6-3	LNPEP	Leucyl-cystinyl aminopeptidase (Isoform 3)	+	-	-	-	0	1	-
Q9Y561	LRP12	Low-density lipoprotein receptor-related protein 12	+	-	-	-	3	1	-
Q9BTT6	LRRC1	Leucine-rich repeat-containing protein 1	+	-	+	-	2	0	[1]
A6NHZ5	LRRC14B	Leucine-rich repeat-containing protein 14B	-	+	-	-	0	0	-
Q8N386	LRRC25	Leucine-rich repeat-containing protein 25	-	+	-	-	0	1	-
Q86X29	LSR	Lipolysis-stimulated lipoprotein receptor	-	+	-	-	12	1	-
P48449	LSS	Lanosterol synthase	-	-	+	-	0	0	[1]
Q9HBG7	LY9	T-lymphocyte surface antigen Ly-9 (Fragment)	-	+	-	-	3	1	[3]
P20645	M6PR	Cation-dependent mannose-6-phosphate receptor	+	+	-	-	2	1	[2], [4]
Q9UKM7	MAN1B1	Endoplasmic reticulum mannosyl-oligosaccharide 1,2-alpha-mannosidase	+	-	-	-	1	1	-
P56192	MARS	Methionine--tRNA ligase, cytoplasmic	-	-	+	-	1	0	[1]
Q68D91	MBLAC2	Metallo-beta-lactamase domain-containing protein 2	+	+	-	-	0	0	[2], [6]
P49736	MCM2	DNA replication licensing factor MCM2	-	-	+	-	1	0	[1]
P33991	MCM4	DNA replication licensing factor MCM4	-	-	+	-	1	0	[1]
Q14566	MCM6	DNA replication licensing factor MCM6	-	-	+	-	2	0	[1]
P33993	MCM7	DNA replication licensing factor MCM7	-	-	+	-	1	0	[1]
Q9GZU1	MCOLN1	Mucolipin-1	+	-	-	-	7	5	-
Q9P1T7	MDFIC	MyoD family inhibitor domain-containing protein	-	+	-	-	11	0	-

UniProt Accession Number	Gene Name	Protein	Enriched in Jurkat T cells	Enriched in Primary T cells	Enriched in Martin et al. 2009	Enriched in Wilson et al. 2011	Predicted Palmitoylation Sites[a]	Predicted Transmembrane Helices[b]	Literature References[c]
P40926	MDH2	Malate dehydrogenase, mitochondrial	-	-	-	+	2	0	[5], [7]
Q9NU22	MDN1	Midasin	-	+	+	-	4	0	[1]
Q6ZSS7	MFSD6	Major facilitator superfamily domain-containing protein 6	-	+	-	-	0	11	[2]
O14880	MGST3	Microsomal glutathione S-transferase 3	-	-	+	-	2	4	[1], [6]
Q29980	MICB	MHC class I polypeptide-related sequence B	+	-	-	-	0	0	-
Q14165	MLEC	Malectin	-	+	+	-	2	1	[1], [2], [3]
Q8IVH4	MMAA	Methylmalonic aciduria type A protein, mitochondrial	+	-	-	-	0	0	-
Q9BRT2	MNF1	Mitochondrial nucleoid factor 1	+	+	-	-	0	0	[4], [5]
P05164	MPO	Myeloperoxidase	+	-	-	-	0	0	-
Q9NZW5	MPP6	MAGUK p55 subfamily member 6	-	+	+	-	0	0	[1], [4], [6]
Q8N565	MREG	Melanoregulin	+	+	+	-	6	0	[1]
P11836	MS4A1	B-lymphocyte antigen CD20	-	+	-	-	1	4	-
P26038	MSN	Moesin	-	-	-	+	0	0	[7]
Q9Y3D2	MSRB2	Methionine-R-sulfoxide reductase B2, mitochondrial	+	-	-	-	0	0	-
P03915	MT-ND5	NADH-ubiquinone oxidoreductase chain 5	-	+	-	-	0	15	-
Q86UE4	MTDH	Protein LYRIC	+	+	+	-	1	1	[1], [2], [3], [5], [8]
Q13614	MTMR2	Myotubularin-related protein 2	-	+	-	-	1	0	-
O75570	MTRF1	Peptide chain release factor 1, mitochondrial	+	-	-	-	1	0	-
Q8WXI7	MUC16	Mucin-16	-	-	+	-	0	1	[5]
Q96S97	MYADM	Myeloid-associated differentiation marker	+	+	+	-	0	8	[1]
P35579	MYH9	Myosin-9	-	-	-	+	0	0	[7], [8]
P60660	MYL6	Myosin light polypeptide 6	-	-	-	+	2	0	[7]
B0I1T2	MYO1G	Unconventional myosin-Ig	-	-	+	+	0	0	[1], [7]
Q9NZM1	MYOF	Myoferlin	-	+	-	-	0	1	[3], [4], [5]
P54920	NAPA	Alpha-soluble NSF attachment protein	-	-	+	-	0	0	[1]
P18440	NAT1	Arylamine N-acetyltransferase 1	-	+	-	-	0	0	-
Q9H0A0	NAT10	N-acetyltransferase 10	-	-	+	-	0	0	[1]
Q6PIU2	NCEH1	Neutral cholesterol ester hydrolase 1	-	+	+	-	1	1	[1]
Q92542	NCSTN	Nicastrin	+	+	-	-	1	1	-
Q9BT67	NDFIP1	NEDD4 family-interacting protein 1	-	-	+	-	1	3	[1], [4]
O14561	NDUFAB1	Acyl carrier protein	+	-	-	-	0	0	-
Q00653	NFKB2	Nuclear factor NF-kappa-B p100 subunit	-	+	-	-	1	0	-
Q13423	NNT	NAD(P) transhydrogenase, mitochondrial	-	-	+	-	1	12	[1], [2], [8]
Q15233	NONO	Non-POU domain-containing octamer-binding protein	-	-	+	-	0	0	[1], [4]
O15118	NPC1	Niemann-Pick C1 protein	+	+	-	-	1	12	[3]
P06748	NPM1	Nucleophosmin	-	-	-	+	0	0	[7], [8]
P01111	NRAS	GTPase NRas	+	+	-	-	2	0	[2], [3], [5]
Q96D31	ORAI1	Calcium release-activated calcium channel protein 1	-	+	-	-	0	3	[2]
Q9NWU1	OXSM	3-oxoacyl-[acyl-carrier-protein] synthase, mitochondrial	+	+	-	-	2	0	[2], [4], [5]
Q99572	P2RX7	P2X purinoceptor 7	-	+	-	-	5	1	[8]
P07237	P4HB	Protein disulfide-isomerase	-	-	-	+	1	0	[6], [7]
Q9UQ80	PA2G4	Proliferation-associated protein 2G4	-	-	+	-	0	0	[1]
Q9UKS6	PACSIN3	Protein kinase C and casein kinase substrate in neurons protein 3	+	-	-	-	2	0	-
Q99487	PAFAH2	Platelet-activating factor acetylhydrolase 2, cytoplasmic	-	-	+	-	1	0	[1]
Q9NWQ8	PAG1	Phosphoprotein associated with glycosphingolipid-enriched microdomains 1	+	+	-	-	2	1	-
Q9H6A9	PCNXL3	Pecanex-like protein 3	-	+	-	-	1	11	-
P11177	PDHB	Pyruvate dehydrogenase E1 component subunit beta, mitochondrial	-	-	+	-	1	0	[1]
O00330	PDHX	Pyruvate dehydrogenase protein X component, mitochondrial	+	+	-	-	0	0	-
Q29RF7	PDS5A	Sister chromatid cohesion protein PDS5 homolog A	-	-	+	-	2	0	[1]
P16284	PECAM1	Platelet endothelial cell adhesion molecule	+	+	-	-	1	1	[5]
Q01813	PFKP	6-phosphofructokinase type C	-	-	+	-	5	0	[1]
P07737	PFN1	Profilin-1	-	-	-	+	0	0	[5], [7], [8]
P18669	PGAM1	Phosphoglycerate mutase 1	-	-	-	+	0	0	[7]
O00264	PGRMC1	Membrane-associated progesterone receptor component 1	-	-	+	-	0	1	[1]
P35232	PHB	Prohibitin	-	-	-	+	1	0	[7]
Q9UPP1	PHF8	Histone lysine demethylase PHF8	-	+	-	-	1	0	-
Q9BTU6	PI4K2A	Phosphatidylinositol 4-kinase type 2-alpha	+	+	+	-	3	0	[1], [2], [3], [5]
Q8TCG2	PI4K2B	Phosphatidylinositol 4-kinase type 2-beta	+	+	+	-	4	0	[1], [2]
P14618	PKM	Pyruvate kinase isozymes M1/M2	-	-	-	+	2	0	[5], [7]
Q99569	PKP4	Plakophilin-4	+	-	-	-	0	0	[8]
O15162	PLSCR1	Phospholipid scramblase 1	+	+	-	-	7	0	[2], [3], [4]
Q9NRY6	PLSCR3	Phospholipid scramblase 3	+	+	-	-	7	0	[2], [3], [4], [5]
O15031	PLXNB2	Plexin-B2	-	+	-	-	1	0	[8]
Q9H307	PNN	Pinin	-	-	+	-	0	0	[1]
O00592	PODXL	Podocalyxin	+	-	-	-	3	1	[5]
A8CG34	POM121C	Nuclear envelope pore membrane protein POM 121C	+	+	-	-	0	1	-
P62937	PPIA	Peptidyl-prolyl cis-trans isomerase A	-	-	-	+	1	0	[7]
P23284	PPIB	Peptidyl-prolyl cis-trans isomerase B	-	-	-	+	0	1	[7], [8]
P62140	PPP1CB	Serine/threonine-protein phosphatase PP1-beta catalytic subunit	-	-	-	+	1	0	[7]
P36873	PPP1CC	Serine/threonine-protein phosphatase	-	-	-	+	1	0	[7]
Q96T49	PPP1R16B	Protein phosphatase 1 regulatory inhibitor subunit 16B	-	+	-	-	2	0	-
O60831	PRAF2	PRA1 family protein 2	-	-	+	-	0	4	[1], [2], [3], [8]
Q06830	PRDX1	Peroxiredoxin-1	-	-	-	+	0	0	[7], [8]
P32119	PRDX2	Peroxiredoxin-2	-	-	-	+	0	0	[7]
P30048	PRDX3	Thioredoxin-dependent peroxide reductase, mitochondrial	-	-	-	+	0	0	[7]
P14314	PRKCSH	Glucosidase 2 subunit beta	-	-	+	-	0	0	[1]
P78527	PRKDC	DNA-dependent protein kinase catalytic subunit	-	-	+	-	7	0	[1]
P55345	PRMT2	Protein arginine N-methyltransferase 2	-	+	-	-	0	0	-
Q9UMS4	PRPF19	Pre-mRNA-processing factor 19	-	-	+	-	2	0	[1], [4]
P49768	PSEN1	Presenilin-1	+	-	+	-	0	9	[1]
P28066	PSMA5	Proteasome subunit alpha type-5	-	-	+	-	1	0	[1]
P60900	PSMA6	Proteasome subunit alpha type-6	-	-	-	+	1	0	[7]
P62191	PSMC1	26S protease regulatory subunit 4	-	-	+	-	0	0	[1]
P35998	PSMC2	26S protease regulatory subunit 7	-	-	+	-	0	0	[1]
P17980	PSMC3	26S protease regulatory subunit 6A	-	-	+	-	0	0	[1]
P43686	PSMC4	26S protease regulatory subunit 6B	-	-	+	-	0	0	[1]
P62195	PSMC5	26S protease regulatory subunit 8	-	-	+	-	0	0	[1]
P62333	PSMC6	26S protease regulatory subunit 10B	-	-	+	-	0	0	[1]
Q99460	PSMD1	26S proteasome non-ATPase regulatory subunit 1	-	-	+	-	1	0	[1]
Q9UNM6	PSMD13	26S proteasome non-ATPase regulatory subunit 13	-	+	+	-	0	0	[1]
Q13200	PSMD2	26S proteasome non-ATPase regulatory subunit 2	-	-	+	-	1	0	[1]
Q9UL46	PSME2	Proteasome activator complex subunit 2	-	-	+	+	1	0	[1], [7]
Q92530	PSMF1	Proteasome inhibitor PI31 subunit	-	+	-	-	0	0	-
O43586	PSTPIP1	Proline-serine-threonine phosphatase-interacting protein 1	-	+	-	-	0	0	-
P26599	PTBP1	Polypyrimidine tract-binding protein 1	-	-	+	-	2	0	[1], [3]
Q13308	PTK7	Inactive tyrosine-protein kinase 7	+	-	+	-	2	2	[1]
Q9P035	PTPLAD1	Very-long-chain (3R)-3-hydroxyacyl-[acyl-carrier protein] dehydratase 3	-	+	-	-	0	5	-
P08575	PTPRC	Receptor-type tyrosine-protein phosphatase C	-	-	+	-	0	2	[1]
Q9Y3E5	PTRH2	Peptidyl-tRNA hydrolase 2, mitochondrial	-	-	+	-	1	1	[1]
P53801	PTTG1IP	Pituitary tumor-transforming gene 1 protein-interacting protein	-	-	+	-	6	2	[1], [2]
P15151-3	PVR	Poliovirus receptor	+	-	-	-	0	0	-
P62820	RAB1A	Ras-related protein Rab-1A	-	-	-	+	2	0	[5], [7]
Q9H0U4	RAB1B	Ras-related protein Rab-1B	-	-	+	+	2	0	[1], [5], [7], [8]
P57729	RAB38	Ras-related protein Rab-38	-	-	+	-	2	0	[5]
P51148	RAB5C	Ras-related protein Rab-5C	-	-	+	-	1	0	[1], [8]
P11234	RALB	Ras-related protein Ral-B	+	-	-	-	2	0	[5], [8]
Q9UKM9	RALY	RNA-binding protein Raly	-	-	+	-	0	0	[1]
P62826	RAN	GTP-binding nuclear protein Ran	-	-	-	+	0	0	[5], [7], [8]
P62834	RAP1A	Ras-related protein Rap-1A	-	-	+	-	1	0	[1]
P61224	RAP1B	Ras-related protein Rap-1b	-	-	+	-	1	0	[1]
P10114	RAP2A	Ras-related protein Rap-2a	-	+	+	-	2	0	[1], [2]
P61225	RAP2B	Ras-related protein Rap-2b	+	+	+	-	3	0	[1], [2], [3], [5]
Q9Y3L5	RAP2C	Ras-related protein Rap-2c	+	+	+	-	3	0	[1], [2], [4], [5]
Q00765	REEP5	Receptor expression-enhancing protein 5	+	+	-	-	0	2	[5], [8]
Q8IUW5	RELL1	RELT-like protein 1	+	+	-	-	1	2	[6]
O15258	RER1	Protein RER1	-	-	+	-	0	3	[1]
Q8WZ73	RFFL	E3 ubiquitin-protein ligase rififylin	-	+	-	-	4	0	-
Q14699	RFTN1	Raftlin	+	+	+	-	1	0	[1], [2]
P22670	RFX1	MHC class II regulatory factor RFX1	+	+	+	-	0	0	[1], [5]
P49795	RGS19	Regulator of G-protein signaling 19	+	-	+	-	9	0	[1], [2]

UniProt Accession Number	Gene Name	Protein	Enriched in Jurkat T cells	Enriched in Primary T cells	Enriched in Martin et al. 2009	Enriched in Wilson et al. 2011	Predicted Palmitoylation Sites[a]	Predicted Transmembrane Helices[b]	Literature References[c]
Q6NTF9	RHBDD2	Rhomboid domain-containing protein 2	+	-	+	-	2	5	[1]
Q9Y3C5	RNF11	RING finger protein 11	-	+	-	-	1	0	[2]
O75695	RP2	Protein XRP2	+	-	-	-	2	0	-
P27635	RPL10	60S ribosomal protein L10	-	-	+	+	0	0	[1], [4], [6], [7]
P62906	RPL10A	60S ribosomal protein L10a	-	-	+	-	0	0	[1], [4], [6]
P30050	RPL12	60S ribosomal protein L12	-	-	+	+	1	0	[1], [4], [5], [7]
P26373	RPL13	60S ribosomal protein L13	-	-	+	-	0	0	[1]
Q02543	RPL18A	60S ribosomal protein L18a	-	-	+	-	0	0	[1], [4]
P62829	RPL23	60S ribosomal protein L23	-	-	+	-	0	0	[1]
P39023	RPL3	60S ribosomal protein L3	-	-	+	-	0	0	[1], [3], [8]
P46777	RPL5	60S ribosomal protein L5	-	-	+	-	0	0	[1], [8]
Q02878	RPL6	60S ribosomal protein L6	-	-	+	-	1	0	[1], [8]
P05388	RPLP0	60S acidic ribosomal protein P0	-	-	-	+	0	0	[4], [7]
P04843	RPN1	Dolichyl-diphosphooligosaccharide--protein glycosyltransferase subunit 1	-	-	+	+	0	1	[7], [8]
P62249	RPS16	40S ribosomal protein S16	-	-	+	+	0	0	[1], [6], [7]
P08708	RPS17	40S ribosomal protein S17	-	-	+	-	0	0	[5]
P15880	RPS2	40S ribosomal protein S2	-	-	+	-	0	0	[1], [4]
P62266	RPS23	40S ribosomal protein S23	-	-	+	-	1	0	[1], [6], [8]
P62857	RPS28	40S ribosomal protein S28	-	-	+	-	0	0	[1]
P23396	RPS3	40S ribosomal protein S3	-	-	-	+	0	0	[7]
P61247	RPS3A	40S ribosomal protein S3a	-	-	+	+	0	0	[1], [7]
P62701	RPS4X	40S ribosomal protein S4, X isoform	-	-	-	+	1	0	[7]
P46782	RPS5	40S ribosomal protein S5	-	-	+	-	1	0	[1], [6]
P62753	RPS6	40S ribosomal protein S6	-	-	+	-	1	0	[1], [5]
P62241	RPS8	40S ribosomal protein S8	-	-	-	+	0	0	[7]
P46781	RPS9	40S ribosomal protein S9	-	-	+	-	1	0	[1]
P10301	RRAS	Ras-related protein R-Ras	+	+	+	-	2	0	[1], [2], [3], [5], [8]
P62070	RRAS2	Ras-related protein R-Ras2	+	+	-	-	2	0	[5], [8]
Q5JTH9	RRP12	RRP12-like protein	-	-	+	-	0	0	[1]
Q96DX4	RSPRY1	RING finger and SPRY domain-containing protein 1	+	-	+	-	3	0	[1]
Q9NQC3	RTN4	Reticulon-4	-	+	+	-	0	2	[1], [5]
Q9Y230	RUVBL2	RuvB-like 2	-	-	+	-	0	0	[1], [4]
O95977	S1PR4	Sphingosine 1-phosphate receptor 4	-	+	-	-	3	7	-
Q9NTJ5	SACM1L	Phosphatidylinositide phosphatase SAC1	-	-	+	-	0	2	[1]
Q99590	SCAF11	Protein SCAF11	+	-	-	-	3	0	-
O15126	SCAMP1	Secretory carrier-associated membrane protein 1	-	+	+	-	0	4	[1], [2], [3], [8]
O15127	SCAMP2	Secretory carrier-associated membrane protein 2	+	+	+	-	0	4	[1], [2], [3], [8]
O14828	SCAMP3	Secretory carrier-associated membrane protein 3	+	+	+	-	0	4	[1], [2], [3], [8]
Q969E2	SCAMP4	Secretory carrier-associated membrane protein 4	-	+	-	-	0	4	[2]
Q8WTV0	SCARB1	Scavenger receptor class B member 1	+	-	-	-	1	2	[3]
Q14108	SCARB2	Lysosome membrane protein 2	+	+	-	-	3	2	[5], [8]
Q8NBX0	SCCPDH	Saccharopine dehydrogenase-like oxidoreductase	-	-	+	-	0	1	[1]
Q14160	SCRIB	Protein scribble homolog	+	+	+	-	1	0	[1], [4]
Q9H190	SDCBP2	Syntenin-2	-	+	-	-	0	0	-
Q6IQ49-3	SDE2	Protein SDE2 homolog	+	-	-	-	1	0	-
Q92854	SEMA4D	Semaphorin-4D	+	+	+	-	2	2	[1], [2], [8]
Q9NVA2	SEPT11	Septin-11	-	+	-	-	0	0	-
Q8NC51	SERBP1	Plasminogen activator inhibitor 1 RNA-binding protein	-	-	+	-	1	0	[1]
Q9NRX5	SERINC1	Serine incorporator 1	+	+	-	-	5	11	-
O75533	SF3B1	Splicing factor 3B subunit 1	-	-	+	-	0	0	[1]
Q15393	SF3B3	Splicing factor 3B subunit 3	-	-	-	+	0	0	[7]
P31947	SFN	14-3-3 protein sigma	-	-	-	+	0	0	[7]
P23246	SFPQ	Splicing factor, proline- and glutamine-rich	-	-	+	-	0	0	[1]
Q8WV19	SFT2D1	Vesicle transport protein SFT2A	-	-	+	-	2	4	[5]
Q587I9	SFT2D3	Vesicle transport protein SFT2C	+	-	+	-	1	4	[1], [2], [4]
P34897	SHMT2	Serine hydroxymethyltransferase, mitochondrial	-	-	+	-	1	0	[1]
Q8NBJ9	SIDT2	SID1 transmembrane family member 2	-	+	-	-	2	9	[8]
Q6IA17	SIGIRR	Single Ig IL-1-related receptor	+	+	-	-	2	1	-
Q8N697	SLC15A4	Solute carrier family 15 member 4	-	+	-	-	2	13	-
O15427	SLC16A3	Monocarboxylate transporter 4 (Fragment)	-	+	-	-	1	12	-
P41440	SLC19A1	Folate transporter 1	+	-	-	-	0	11	-
P43007	SLC1A4	Neutral amino acid transporter A	-	-	+	-	0	9	[1], [8]
Q15758	SLC1A5	Neutral amino acid transporter B(0)	+	+	+	+	0	9	[1], [3], [4], [5], [7]
Q02978	SLC25A11	Mitochondrial 2-oxoglutarate/malate carrier protein	-	-	+	-	0	0	[1]
Q00325	SLC25A3	Phosphate carrier protein, mitochondrial	-	-	+	-	0	2	[1], [8]
P12235	SLC25A4	ADP/ATP translocase 1	-	-	-	+	0	3	[7]
P05141	SLC25A5	ADP/ATP translocase 2	-	-	-	+	0	3	[7]
P12236	SLC25A6	ADP/ATP translocase 3	-	-	+	+	0	2	[1], [7]
Q6NXT4	SLC30A6	Zinc transporter 6	-	+	-	-	3	6	-
Q8TB61	SLC35B2	Adenosine 3'-phospho 5'-phosphosulfate transporter 1	-	+	+	-	0	8	[1], [3], [4]
Q8TB61-3	SLC35B2	Adenosine 3-phospho 5-phosphosulfate transporter 1 (Isoform 3)	+	-	-	-	0	7	-
Q96K37	SLC35E1	Solute carrier family 35 member E1	-	+	-	-	0	6	-
P0CK97	SLC35E2	Solute carrier family 35 member E2	-	-	+	-	1	3	[5]
P0CK96	SLC35E2B	Solute carrier family 35 member E2B	-	+	-	-	1	8	-
Q96QD8	SLC38A2	Sodium-coupled neutral amino acid transporter 2	+	-	+	-	0	9	[1], [6]
Q9ULF5	SLC39A10	Zinc transporter ZIP10	+	-	-	-	2	7	[8]
Q96GZ6	SLC41A3	Solute carrier family 41 member 3	-	+	-	-	0	10	-
Q8NBI5	SLC43A3	Solute carrier family 43 member 3	+	-	+	-	1	11	[1], [2]
Q8WWI5	SLC44A1	Choline transporter-like protein 1	+	+	-	-	9	9	[2], [3], [8]
Q8IWA5	SLC44A2	Choline transporter-like protein 2	-	+	-	-	6	11	[2], [8]
Q9Y289	SLC5A6	Sodium-dependent multivitamin transporter	-	-	+	-	1	14	[1], [4]
P30825	SLC7A1	High affinity cationic amino acid transporter 1	+	-	+	-	2	14	[1], [4], [8]
Q92581	SLC9A6	Sodium/hydrogen exchanger 6	-	-	+	-	1	12	[1], [8]
Q92581-3	SLC9A6	Sodium/hydrogen exchanger 6 (Isoform 3)	+	-	-	-	1	11	-
Q92581-2	SLC9A6	Isoform 2 of Sodium/hydrogen exchanger 6	-	+	-	-	1	12	-
Q8IVB4	SLC9A9	Sodium/hydrogen exchanger 9	-	+	-	-	0	10	[2]
Q9UIG8	SLCO3A1	Solute carrier organic anion transporter family member 3A1	-	+	-	-	2	12	-
P51532	SMARCA4	Transcription activator BRG1	-	-	+	-	0	0	[5]
B2RUZ4	SMIM1	Small integral membrane protein 1	-	+	-	-	1	1	-
Q9NY59	SMPD3	Sphingomyelin phosphodiesterase 3	-	+	-	-	6	2	-
O00161	SNAP23	Synaptosomal-associated protein 23	+	+	+	+	6	0	[1], [2], [3], [4], [5], [6], [7]
P60880-2	SNAP25	Synaptosomal-associated protein 25 (Isoform 2)	+	-	-	-	4	0	-
O75643	SNRNP200	U5 small nuclear ribonucleoprotein 200 kDa helicase	-	-	+	-	2	0	[1]
P62308	SNRPG	Small nuclear ribonucleoprotein G	-	-	+	-	0	0	[1]
Q9Y5W9	SNX11	Sorting nexin-11	+	-	-	-	2	0	-
Q99523	SORT1	Sortilin	+	+	+	-	1	1	[1], [2], [8]
Q8TCT8	SPPL2A	Signal peptide peptidase-like 2A	+	+	-	-	1	9	-
Q7Z698	SPRED2	Sprouty-related, EVH1 domain-containing protein 2	+	-	-	-	3	0	[8]
O43609	SPRY1	Protein sprouty homolog 1	+	-	-	-	1	0	-
Q5W111	SPRYD7	SPRY domain-containing protein 7	+	-	-	-	2	0	[4]
Q13813	SPTAN1	Spectrin alpha chain, non-erythrocytic 1	-	-	-	+	0	0	[7]
Q9NUV7	SPTLC3	Serine palmitoyltransferase 3 (Fragment)	-	+	-	-	0	0	-
Q9Y5M8	SRPRB	Signal recognition particle receptor subunit beta	-	-	+	-	0	1	[1]
Q13247	SRSF6	Serine/arginine-rich splicing factor 6	-	-	+	-	0	0	[1]
O95772	STARD3NL	MLN64 N-terminal domain homolog	-	+	-	-	0	4	[2]
O75716	STK16	Serine/threonine-protein kinase 16	-	+	-	-	0	0	-
Q9NZ72	STMN3	Stathmin	+	-	-	-	1	0	-
P27105	STOM	Erythrocyte band 7 integral membrane protein	-	+	+	-	2	1	[1], [2], [3], [4], [5]
Q9UJZ1	STOML2	Stomatin-like protein 2, mitochondrial	-	-	+	-	0	0	[1]
Q8TCJ2	STT3B	Dolichyl-diphosphooligosaccharide--protein glycosyltransferase subunit STT3B	-	-	+	-	1	10	[1]
O60499	STX10	Syntaxin-10	+	-	+	-	1	1	[1], [2]
O75558	STX11	Syntaxin-11	+	+	-	-	6	0	[2], [5]
Q86Y82	STX12	Syntaxin-12	+	+	+	-	1	1	[1], [2], [3], [5], [8]
P32856-3	STX2	Syntaxin-2 (Isoform 3)	+	-	-	-	0	0	-
Q12846	STX4	Syntaxin-4	+	-	+	-	1	1	[1]
O43752	STX6	Syntaxin-6	+	+	+	-	1	1	[1], [3], [5], [8]
O15400	STX7	Syntaxin-7	+	+	+	-	1	1	[1], [3], [8]
Q9UNK0	STX8	Syntaxin-8	+	+	+	-	1	1	[1], [2], [3], [8]
O15260	SURF4	Surfeit locus protein 4	-	+	+	-	0	5	[1], [8]
Q96L08	SUSD3	Sushi domain-containing protein 3	-	+	-	-	2	1	[2]

UniProt Accession Number	Gene Name	Protein	Enriched in Jurkat T cells	Enriched in Primary T cells	Enriched in Martin et al. 2009	Enriched in Wilson et al. 2011	Predicted Palmitoylation Sites[a]	Predicted Transmembrane Helices[b]	Literature References[c]
Q8NHG7	SVIP	Small VCP/p97-interacting protein	+	-	-	-	1	0	[2], [5]
Q6ZMZ3	SYNE3	Nesprin-3	-	+	-	-	1	1	-
Q03518	TAP1	Antigen peptide transporter 1	-	-	+	-	1	7	[1]
Q6NXT6	TAPT1	Transmembrane anterior posterior transformation protein 1 homolog	-	+	-	-	1	5	-
P17987	TCP1	T-complex protein 1 subunit alpha	-	-	-	+	0	0	[7], [8]
Q96BS2	TESC	Calcineurin B homologous protein 3	+	+	-	-	1	0	-
P02787	TF	Serotransferrin	+	-	-	-	2	0	-
Q9H5Q4	TFB2M	Dimethyladenosine transferase 2, mitochondrial	+	-	-	-	0	0	-
P02786	TFRC	Transferrin receptor protein 1	-	+	+	-	0	1	[1], [2], [5]
Q8WUY1	THEM6	Protein THEM6	-	+	-	-	0	1	-
Q9Y490	TLN1	Talin-1	-	-	-	+	4	0	[7]
Q99805	TM9SF2	Transmembrane 9 superfamily member 2	-	+	-	-	0	9	[6], [8]
Q7Z403	TMC6	Transmembrane channel-like protein 6	-	+	-	-	0	10	-
Q8IU68	TMC8	Transmembrane channel-like protein 8	-	+	-	-	1	8	-
Q13445	TMED1	Transmembrane emp24 domain-containing protein 1	+	+	+	-	1	1	[1]
Q12893	TMEM115	Transmembrane protein 115	-	-	+	-	1	6	[1]
Q9H6X4	TMEM134	Transmembrane protein 134	+	+	-	-	2	2	-
Q7Z7N9	TMEM179B	Transmembrane protein 179B	-	+	-	-	2	3	-
Q8IY95	TMEM192	Transmembrane protein 192	+	+	-	-	1	4	-
Q9H813	TMEM206	Transmembrane protein 206	+	-	+	-	0	1	[1]
Q9H0R3	TMEM222	Transmembrane protein 222	-	+	+	-	1	3	[1], [2]
Q9H330	TMEM245	Transmembrane protein 245	-	+	-	-	2	14	-
Q86T03	TMEM55B	Transmembrane protein 55B	-	+	+	-	1	2	[1], [2], [3], [8]
O94886	TMEM63A	Transmembrane protein 63A	+	+	+	-	4	11	[1], [2]
Q8NBN3	TMEM87A	Transmembrane protein 87A	-	+	-	-	0	7	-
Q96BF3	TMIGD2	Transmembrane and immunoglobulin domain-containing protein 2	+	+	-	-	0	1	-
Q6ZT21	TMPPE	Transmembrane protein with metallophosphoesterase domain	-	+	-	-	1	5	[2]
Q9H3N1	TMX1	Thioredoxin-related transmembrane protein 1	+	+	+	+	0	3	[1], [2], [3], [5], [7], [8]
Q96JJ7	TMX3	Protein disulfide-isomerase TMX3	+	+	+	-	1	1	[1], [2]
Q9H1E5	TMX4	Thioredoxin-related transmembrane protein 4	+	+	+	-	1	1	[1], [2]
P28908	TNFRSF8	Tumor necrosis factor receptor superfamily member 8	+	-	-	-	1	1	-
O43557	TNFSF14	Tumor necrosis factor ligand superfamily member 14	-	+	-	-	0	1	-
O96008	TOMM40	Mitochondrial import receptor subunit TOM40 homolog	-	-	+	-	0	0	[1]
P60174	TPI1	Triosephosphate isomerase	-	-	-	+	0	0	[7]
Q9Y228	TRAF3IP3	TRAF3-interacting JNK-activating modulator	+	+	+	-	0	1	[1]
Q12931	TRAP1	Heat shock protein 75 kDa, mitochondrial	-	-	+	-	0	0	[1]
O43617	TRAPPC3	Trafficking protein particle complex subunit 3	+	-	-	-	1	0	[2], [3], [5]
Q86YW5	TREML1	Trem-like transcript 1 protein	-	+	-	-	0	1	-
P49815	TSC2	Tuberin	-	+	-	-	0	0	-
Q8NG11	TSPAN14	Tetraspanin-14	+	-	-	-	1	4	[2], [6]
O95858	TSPAN15	Tetraspanin-15	+	-	-	-	3	4	[2]
Q96SJ8	TSPAN18	Tetraspanin-18 (Fragment)	-	+	-	-	6	4	-
Q86UF1	TSPAN33	Tetraspanin-33	+	+	-	-	3	4	[2]
P41732	TSPAN7	Tetraspanin-7	+	-	-	-	4	4	-
O75954	TSPAN9	Tetraspanin-9	-	+	-	-	4	4	[2]
Q8NFU3	TSTD1	Thiosulfate sulfurtransferase/rhodanese-like domain-containing protein 1	+	-	-	-	0	0	-
Q8WZ42	TTN	Titin	-	-	+	-	13	0	[1]
Q9C0H2	TTYH3	Protein tweety homolog 3	-	+	-	-	3	5	[2], [8]
Q71U36	TUBA1A	Tubulin alpha-1A chain	-	-	-	+	8	0	[7]
P49411	TUFM	Elongation factor Tu, mitochondrial	-	-	+	-	0	0	[1], [6]
O15042	U2SURP	U2 snRNP-associated SURP motif-containing protein	-	-	+	-	0	0	[1]
Q9Y3C8	UFC1	Ubiquitin-fold modifier-conjugating enzyme 1	+	-	-	-	1	0	-
O75385	ULK1	Serine/threonine-protein kinase ULK1	-	+	-	-	3	0	-
Q96IX5	USMG5	Up-regulated during skeletal muscle growth protein 5	-	-	+	-	0	1	[1], [8]
P63027	VAMP2	Vesicle-associated membrane protein 2	-	+	+	-	0	1	[1], [5], [8]
Q15836	VAMP3	Vesicle-associated membrane protein 3	+	+	+	-	0	1	[1], [2], [3], [5]
O75379	VAMP4	Vesicle-associated membrane protein 4	+	+	+	-	0	1	[1], [2], [3], [8]
O95183	VAMP5	Vesicle-associated membrane protein 5	+	-	+	-	0	1	[1], [2], [3]
P51809	VAMP7	Vesicle-associated membrane protein 7	-	-	+	-	3	1	[1], [2], [3], [8]
Q8TAA9	VANGL1	Vang-like protein 1	-	-	+	-	0	4	[1]
Q9P0L0	VAPA	Vesicle-associated membrane protein-associated protein A	-	-	+	-	0	1	[1], [4], [8]
Q99536	VAT1	Synaptic vesicle membrane protein VAT-1 homolog	-	-	-	+	0	0	[7]
P55072	VCP	Transitional endoplasmic reticulum ATPase	-	-	-	+	0	0	[7]
P21796	VDAC1	Voltage-dependent anion-selective channel protein 1	-	-	-	+	0	0	[7], [8]
P45880	VDAC2	Voltage-dependent anion-selective channel protein 2	-	-	-	+	1	0	[5], [7], [8]
Q9Y277	VDAC3	Voltage-dependent anion-selective channel protein 3	-	-	-	+	1	0	[5], [7], [8]
P08670	VIM	Vimentin	-	-	+	-	0	0	[1]
O14980	XPO1	Exportin-1	-	-	+	-	0	0	[1], [8]
P13010	XRCC5	X-ray repair cross-complementing protein 5	-	-	-	+	0	0	[7]
P12956	XRCC6	X-ray repair cross-complementing protein 6	-	-	-	+	0	0	[7]
P07947	YES1	Tyrosine-protein kinase Yes	-	-	+	-	1	0	[1], [2], [4], [5]
P31946	YWHAB	14-3-3 protein beta/alpha	-	-	-	+	0	0	[7]
P62258	YWHAE	14-3-3 protein epsilon	-	-	-	+	2	0	[7]
P61981	YWHAG	14-3-3 protein gamma	-	-	+	+	1	0	[1], [7]
Q04917	YWHAH	14-3-3 protein eta	-	-	-	+	1	0	[7]
P27348	YWHAQ	14-3-3 protein theta	-	-	-	+	1	0	[7]
P63104	YWHAZ	14-3-3 protein zeta/delta	-	-	-	+	0	0	[5], [7]
Q8IUH4	ZDHHC13	Palmitoyltransferase ZDHHC13	+	-	-	-	1	6	-
Q8IUH5	ZDHHC17	Palmitoyltransferase ZDHHC17	+	+	+	-	0	6	[1]
Q9NUE0	ZDHHC18	Palmitoyltransferase ZDHHC18	-	+	-	-	2	4	-
Q5W0Z9	ZDHHC20	Probable palmitoyltransferase ZDHHC20	-	+	+	-	2	4	[1]
Q9C0B5	ZDHHC5	Palmitoyltransferase ZDHHC5	+	+	+	-	2	4	[1], [4]
Q9H6R6	ZDHHC6	Palmitoyltransferase ZDHHC6	-	-	+	-	2	4	[1], [4]

Supplementary Table 3

Analysis of palmitoylated proteins enriched in Jurkat T cells and primary T cells. Novel candidates (not reported in the eight published proteomic studies analyzed here) with at least one predicted palmitoylation site are shown in blue. [a]Palmitoylation sites predicted using CSS-Palm (version 3.0), with a "High" threshold and a cutoff score of 1.0. [b]Prediction of transmembrane domains was performed using TMHMM Server (version 2.0). [c]References: [1] Martin and Cravatt, Nat. Meth., 2009, [2] Dowal et al., Blood, 2011, [3] Merrick et al., Mol. & Cell. Proteom., 2011, [4] Yang et al., Mol. & Cell. Proteom., 2010, [5] Marin et al., Circ. Res., 2012, [6] Forrester et al., J. Lip. Res., 2011, [7] Wilson et al., Mol. & Cell. Proteom., 2011, [8] Kang et al., Nature, 2008.

UniProt Accession Number	Gene Name	Protein	Enriched in Jurkat T cells	Enriched in Primary T cells	Predicted Palmitoylation Sites[a]	Predicted Transmembrane Helices[b]	Literature References[c]
Q9NP58	ABCB6	ATP-binding cassette sub-family B member 6, mitochondrial	+	-	0	9	[2]
O15439	ABCC4	Multidrug resistance-associated protein 4	-	+	2	11	[2]
O95870	ABHD16A	Abhydrolase domain-containing protein 16A	-	+	1	2	[1], [8]
Q96GS6	ABHD17A	Alpha/beta hydrolase domain-containing protein 17A	+	+	4	0	[1]
Q5VST6	ABHD17B	Alpha/beta hydrolase domain-containing protein 17B	+	+	4	0	[1], [3], [7]
P09110	ACAA1	3-ketoacyl-CoA thiolase, peroxisomal	+	+	0	0	-
P24752	ACAT1	Acetyl-CoA acetyltransferase, mitochondrial	+	+	0	0	-
Q9BWD1	ACAT2	Acetyl-CoA acetyltransferase, cytosolic	+	+	2	0	-
O14734	ACOT8	Acyl-coenzyme A thioesterase 8	-	+	0	0	-
P33121	ACSL1	Long-chain-fatty-acid--CoA ligase 1	-	+	0	1	-
O43707	ACTN4	Alpha-actinin-4	-	+	0	0	[4], [5]
O14672	ADAM10	Disintegrin and metalloproteinase domain-containing protein 10	-	+	2	1	[1], [8]
P78536	ADAM17	Disintegrin and metalloproteinase domain-containing protein 17	+	+	4	1	[3]
O43306	ADCY6	Adenylate cyclase type 6	-	+	0	12	[2], [8]
P51828	ADCY7	Adenylate cyclase type 7	+	+	4	12	-
Q99943	AGPAT1	1-acyl-sn-glycerol-3-phosphate acyltransferase alpha	+	-	0	3	[1], [3], [8]
Q9NUQ2	AGPAT5	1-acyl-sn-glycerol-3-phosphate acyltransferase epsilon	-	+	1	3	-
P43353	ALDH3B1	Aldehyde dehydrogenase family 3 member B1	-	+	2	0	[3]
Q02252	ALDH6A1	Methylmalonate-semialdehyde dehydrogenase [acylating], mitochondrial	+	+	1	1	-
Q86YT9	AMICA1	Junctional adhesion molecule-like	-	+	2	1	-
Q9H6X2	ANTXR1	Anthrax toxin receptor 1	+	-	4	1	[1]
P58335	ANTXR2	Anthrax toxin receptor 2	-	+	5	1	[5]
Q63HQ0	AP1AR	AP-1 complex-associated regulatory protein	+	+	3	0	[1]
Q9NXU5	ARL15	ADP-ribosylation factor-like protein 15	+	+	0	0	[1], [2], [3], [8]
Q8N6S5	ARL6IP6	ADP-ribosylation factor-like protein 6-interacting protein 6	+	+	1	3	[1]
P98196	ATP11A	Probable phospholipid-transporting ATPase IH	-	+	3	7	[4]
Q9Y2G3	ATP11B	Probable phospholipid-transporting ATPase IF	+	+	4	8	[1], [2]
Q8NB49	ATP11C	Probable phospholipid-transporting ATPase IG	-	+	0	7	-
Q9Y487	ATP6V0A2	V-type proton ATPase 116 kDa subunit a isoform 2	+	+	1	6	[1], [2]
O43861	ATP9B	Probable phospholipid-transporting ATPase IIB	+	-	1	0	[1]
O75882	ATRN	Attractin	+	-	4	1	[8]
Q9Y679	AUP1	Ancient ubiquitous protein 1	+	+	1	2	[1]
Q9Y679-3	AUP1	Ancient ubiquitous protein 1 (Isoform 3)	+	-	1	0	-
O43505	B3GNT1	N-acetyllactosaminide beta-1,3-N-acetylglucosaminyltransferase	+	-	1	1	[1]
P15291	B4GALT1	Beta-1,4-galactosyltransferase 1	+	+	1	1	[1]
Q9UBV7	B4GALT7	Beta-1,4-galactosyltransferase 7	+	+	1	1	-
Q9HB09	BCL2L12	Bcl-2-like protein 12	+	-	0	0	-
O15155	BET1	BET1 homolog	-	+	0	1	[2]
Q9NYM9	BET1L	BET1-like protein	+	-	0	1	[2]
Q10589	BST2	Bone marrow stromal antigen 2	+	+	2	1	-
Q8N357	C2orf18	Solute carrier family 35 member F6	-	+	0	10	-
Q6P1X6	C8orf82	UPF0598 protein C8orf82	+	-	1	0	[2]
Q9HA72	CALHM2	Calcium homeostasis modulator protein 2	-	+	1	4	-
P27824	CANX	Calnexin	+	+	2	1	[1], [3], [5], [7], [8]
O15484	CAPN5	Calpain-5	-	+	1	0	[4], [5], [8]
A6NI79	CCDC69	Coiled-coil domain-containing protein 69	-	+	1	0	-
Q8ND76	CCNY	Cyclin-Y	+	+	2	0	[1], [2], [3]
Q8ND76-3	CCNY	Cyclin-Y	+	-	0	0	-
Q8N7R7	CCNYL1	Cyclin-Y-like protein 1	+	-	2	0	[1]
P51681	CCR5	C-C chemokine receptor type 5	-	+	4	6	-
P48509	CD151	CD151 antigen	-	+	7	4	[2], [6]
P29017	CD1C	T-cell surface glycoprotein CD1c	+	-	1	1	-
Q8TD46	CD200R1	Cell surface glycoprotein CD200 receptor 1	-	+	1	2	-
P26842	CD27	CD27 antigen	-	+	1	1	-
P16671	CD36	Platelet glycoprotein 4 (Fragment)	-	+	4	2	[2], [3]
P11049	CD37	Leukocyte antigen CD37	-	+	4	4	-
P28907	CD38	ADP-ribosyl cyclase 1	+	+	2	1	[1], [2], [4], [8]
P04234	CD3D	T-cell surface glycoprotein CD3 delta chain	+	+	0	0	[1]
P01730	CD4	T-cell surface glycoprotein CD4	-	+	3	1	[1]
P16070	CD44	CD44 antigen (Fragment)	-	+	2	1	[3], [5]
P06127	CD5	T-cell surface glycoprotein CD5	+	-	2	1	[1]
P19397	CD53	Leukocyte surface antigen CD53	+	+	4	4	-
P08962	CD63	CD63 antigen	+	+	7	4	[2], [5]
P09564	CD7	T-cell antigen CD7	+	+	2	1	-
P21854	CD72	B-cell differentiation antigen CD72	-	+	2	1	-
P27701	CD82	CD82 antigen	+	+	5	4	[1], [2], [5], [7]
Q01151	CD83	CD83 antigen	-	+	0	1	-
P21926	CD9	CD9 antigen	-	+	6	4	[5]
P40200	CD96	T-cell surface protein tactile	-	+	1	1	-
P48960-2	CD97	CD97 antigen (Isoform 2)	+	-	0	7	-
P48960	CD97	CD97 antigen	-	+	0	7	[4]
P14209	CD99	CD99 antigen	+	+	0	2	[5], [6]
Q8TCZ2	CD99L2	CD99 antigen-like protein 2	-	+	1	1	[2], [8]
Q5VV42	CDKAL1	Threonylcarbamoyladenosine tRNA methylthiotransferase	-	+	1	1	-
Q9NPF2	CHST11	Carbohydrate sulfotransferase 11	+	-	1	1	[1]
Q07065	CKAP4	Cytoskeleton-associated protein 4	+	+	1	1	[3], [4], [5]
Q9NY35	CLDND1	Claudin domain-containing protein 1	-	+	2	4	-
O14967	CLGN	Calmegin	+	-	1	1	[1]
Q96DZ5	CLIP3	CAP-Gly domain-containing linker protein 3	+	-	2	0	[8]
Q6PJW8	CNST	Consortin	+	+	2	1	-
Q86VU5	COMTD1	Catechol O-methyltransferase domain-containing protein 1 (Fragment)	-	+	0	1	[2]
Q9UI42	CPA4	Carboxypeptidase A4	+	-	0	0	-
Q9HCP0	CSNK1G1	Casein kinase I isoform gamma-1	+	+	3	0	[1], [2], [8]
P78368	CSNK1G2	Casein kinase I isoform gamma-2	+	+	3	0	[8]
Q9Y6M4-3	CSNK1G3	Casein kinase I isoform gamma-3 (Isoform 3)	+	-	3	0	-
Q9Y6M4	CSNK1G3	Casein kinase I isoform gamma-3	-	+	3	0	[1], [2], [8]
Q9GZU7-2	CTDSP1	Carboxy-terminal domain RNA polymerase II polypeptide A small phosphatase 1	+	+	0	0	-
Q9GZU7	CTDSP1	Carboxy-terminal domain RNA polymerase II polypeptide A small phosphatase 1	-	+	0	0	[1]
O14595	CTDSP2	Carboxy-terminal domain RNA polymerase II polypeptide A small phosphatase 2	-	+	0	0	-
P78310-2	CXADR	Coxsackievirus and adenovirus receptor (Isoform 2)	+	-	2	1	-
P04839	CYBB	Cytochrome b-245 heavy chain	-	+	2	4	[3]
Q9Y4D1	DAAM1	Disheveled-associated activator of morphogenesis 1	+	+	1	0	[1], [2], [8]
Q8NCG7	DAGLB	Sn1-specific diacylglycerol lipase beta	+	+	3	4	[1], [2], [3]
Q8IWE4	DCUN1D3	DCN1-like protein 3	+	+	2	0	-
Q9BSY9	DESI2	Desumoylating isopeptidase 2	+	-	0	0	-

UniProt Accession Number	Gene Name	Protein	Enriched in Jurkat T cells	Enriched in Primary T cells	Predicted Palmitoylation Sites[a]	Predicted Transmembrane Helices[b]	Literature References[c]
Q9P202	DFNB31	Whirlin	-	+	0	0	-
P52429	DGKE	Diacylglycerol kinase epsilon	-	+	2	1	-
Q9Y2H0	DLGAP4	Disks large-associated protein 4	+	-	2	0	[2]
O75190-3	DNAJB6	DnaJ homolog subfamily B member 6	-	+	0	0	-
Q9H3Z4	DNAJC5	DnaJ homolog subfamily C member 5	+	+	13	1	[1], [2], [3]
Q14126	DSG2	Desmoglein-2	+	-	1	2	[1]
O00559	EBAG9	Receptor-binding cancer antigen expressed on SiSo cells	+	-	1	0	[1], [2], [8]
P42892-3	ECE1	Endothelin-converting enzyme 1	+	-	0	1	-
P42892	ECE1	Endothelin-converting enzyme 1	+	+	0	1	[2], [5]
O75071	EFCAB14	EF-hand calcium-binding domain-containing protein 14	+	-	2	1	[1]
Q14156	EFR3A	Protein EFR3 homolog A	-	+	4	0	[2], [3]
Q7L2H7	EIF3M	Eukaryotic translation initiation factor 3 subunit M	-	+	2	0	-
Q9NYP7	ELOVL5	Elongation of very long chain fatty acids protein 5	-	+	1	7	-
Q6PCB8	EMB	Embigin	+	-	0	1	-
P54852	EMP3	Epithelial membrane protein 3 (Fragment)	-	+	1	4	-
Q9Y227	ENTPD4	Ectonucleoside triphosphate diphosphohydrolase 4	-	+	0	2	-
Q96RT1-7	ERBB2IP	Protein LAP2 (Isoform 7)	+	-	3	0	-
Q96RT1	ERBB2IP	Protein LAP2	-	+	3	0	[1], [2]
Q969X5	ERGIC1	Endoplasmic reticulum-Golgi intermediate compartment protein 1	+	+	1	2	[3]
Q96RQ1	ERGIC2	Endoplasmic reticulum-Golgi intermediate compartment protein 2	-	+	0	0	[1]
Q9Y282	ERGIC3	Endoplasmic reticulum-Golgi intermediate compartment protein 3	+	+	0	2	[1], [2], [3], [5], [8]
P34910	EVI2B	Protein EVI2B	-	+	1	1	-
Q92817	EVPL	Envoplakin	-	+	2	0	-
P05413	FABP3	Fatty acid-binding protein, heart	+	-	1	0	-
A8MVW0	FAM171A2	Protein FAM171A2	+	-	2	2	-
Q8N128	FAM177A1	Protein FAM177A1	+	+	0	0	-
Q9NUQ9	FAM49B	Protein FAM49B	+	+	0	0	[1], [2]
P25445	FAS	Tumor necrosis factor receptor superfamily member 6	+	+	2	0	-
Q9Y3I1	FBXO7	F-box only protein 7	-	+	0	0	-
P02671	FGA	Fibrinogen alpha chain	+	-	1	0	-
P02675	FGB	Fibrinogen beta chain	+	-	0	0	-
P09769	FGR	Tyrosine-protein kinase Fgr	-	+	2	0	-
O75955	FLOT1	Flotillin-1	+	+	1	0	[1], [2], [3], [4], [5]
Q14254	FLOT2	Flotillin-2	+	+	3	0	[1], [2], [3], [5]
Q9Y2H6	FNDC3A	Fibronectin type-III domain-containing protein 3A	-	+	1	1	[8]
P06241-3	FYN	Tyrosine-protein kinase Fyn (Isoform 3)	+	-	2	0	-
P06241	FYN	Tyrosine-protein kinase Fyn	-	+	4	0	[1], [2], [3], [5]
Q10472	GALNT1	Polypeptide N-acetylgalactosaminyltransferase 1	+	+	1	1	-
Q8IXK2	GALNT12	Polypeptide N-acetylgalactosaminyltransferase 12	+	-	1	1	[1]
Q9NY12	GAR1	H/ACA ribonucleoprotein complex subunit 1	+	-	0	0	-
Q9UG22	GIMAP2	GTPase IMAP family member 2	-	+	3	2	-
P29992	GNA11	Guanine nucleotide-binding protein subunit alpha-11	-	+	2	0	[1], [2], [4], [5], [6]
Q03113	GNA12	Guanine nucleotide-binding protein subunit alpha-12	-	+	0	0	[4]
Q14344	GNA13	Guanine nucleotide-binding protein subunit alpha-13	+	+	2	0	[1], [2], [4], [5]
P30679	GNA15	Guanine nucleotide-binding protein subunit alpha-15	+	-	3	0	[1], [2]
P09471-2	GNAO1	Isoform Alpha-2 of Guanine nucleotide-binding protein G(o) subunit alpha	-	+	1	0	-
P50148	GNAQ	Guanine nucleotide-binding protein G(q) subunit alpha	+	+	2	0	[1], [2], [3], [4], [5]
P63092	GNAS	Guanine nucleotide-binding protein G(s) subunit alpha isoforms short	-	+	1	0	[6]
P19086	GNAZ	Guanine nucleotide-binding protein G(z) subunit alpha	-	+	2	0	-
Q7Z5G4	GOLGA7	Golgin subfamily A member 7	+	+	0	0	[1], [2], [3]
Q2TAP0	GOLGA7B	Golgin subfamily A member 7B	-	+	0	0	-
Q14789	GOLGB1	Golgin subfamily B member 1	+	+	2	1	[1]
Q99795	GPA33	Cell surface A33 antigen	-	+	6	1	-
Q9NPR9	GPR108	Protein GPR108	-	+	0	7	-
P32249	GPR183	G-protein coupled receptor 183	-	+	0	7	-
Q15743	GPR68	Ovarian cancer G-protein coupled receptor 1	-	+	1	7	-
Q6ZVF9	GPRIN3	G protein-regulated inducer of neurite outgrowth 3	-	+	1	0	[8]
P07203	GPX1	Glutathione peroxidase 1	+	-	2	0	[8]
Q8WW33	GTSF1	Gametocyte-specific factor 1	+	-	0	0	-
Q9BSH5	HDHD3	Haloacid dehalogenase-like hydrolase domain-containing protein 3	-	+	1	0	-
O75146	HIP1R	Huntingtin-interacting protein 1-related protein	-	+	1	0	-
P01889	HLA-B	HLA class I histocompatibility antigen, B-7 alpha chain	+	-	2	1	[1], [4]
P30504	HLA-C	HLA class I histocompatibility antigen, Cw-4 alpha chain	+	+	2	1	-
P30501	HLA-C	HLA class I histocompatibility antigen, Cw-2 alpha chain	-	+	1	1	-
P00738	HP	Haptoglobin	+	-	0	0	-
P01112	HRAS	GTPase HRas	+	-	3	0	[1], [2], [5], [6]
P13598	ICAM2	Intercellular adhesion molecule 2	+	+	2	1	[5]
Q01629	IFITM2	Interferon-induced transmembrane protein 1	+	-	3	2	[3], [5]
Q01628	IFITM3	Interferon-induced transmembrane protein 3	-	+	3	2	[2], [3], [5]
P17181	IFNAR1	Interferon alpha/beta receptor 1	+	-	1	2	-
P11717	IGF2R	Cation-independent mannose-6-phosphate receptor	+	-	3	1	[1], [3], [7], [8]
Q969P0	IGSF8	Immunoglobulin superfamily member 8	+	+	2	1	[1], [8]
P24001-4	IL32	Interleukin-32 (Isoform 4)	+	-	1	0	-
Q71H61	ILDR2	Immunoglobulin-like domain-containing receptor 2	+	-	12	1	-
Q9H0X4	ITFG3	Protein ITFG3	+	+	2	1	-
P23229-4	ITGA6	Integrin alpha-6 (Isoform 4)	+	-	2	1	-
P23229	ITGA6	Integrin alpha-6	-	+	1	1	[5]
O43736	ITM2A	Integral membrane protein 2A	+	+	1	1	[1]
Q9Y287	ITM2B	Integral membrane protein 2B	+	-	1	1	-
Q9NQX7	ITM2C	Integral membrane protein 2C	+	-	2	1	[8]
P23458	JAK1	Tyrosine-protein kinase JAK1	-	+	3	0	-
Q9BX67	JAM3	Junctional adhesion molecule C	+	+	3	1	[2], [8]
Q9Y4C1	KDM3A	Lysine-specific demethylase 3A	-	+	0	0	-
Q8IZA0	KIAA0319L	Dyslexia-associated protein KIAA0319-like protein	+	+	4	1	-
A2VDJ0	KIAA0922	Transmembrane protein 131-like	+	-	3	0	-
Q9BY89	KIAA1671	Uncharacterized protein KIAA1671	-	+	0	0	-
Q8IYS2	KIAA2013	Uncharacterized protein KIAA2013	+	+	0	2	[1], [2], [3]
P43630	KIR3DL2	Killer cell immunoglobulin-like receptor 3DL2	+	-	1	1	-
Q6UWL6-3	KIRREL2	Kin of IRRE-like protein 2 (Isoform 3)	+	-	2	1	-
Q8NBE8	KLHL23	Kelch-like protein 23	-	+	0	0	-
Q6GTX8	LAIR1	Leukocyte-associated immunoglobulin-like receptor 1	+	-	1	1	-
Q6IAA8	LAMTOR1	Ragulator complex protein LAMTOR1	+	-	2	0	[1], [5], [7]
O43561	LAT	Linker for activation of T-cells family member 1	+	+	3	1	[1], [2], [3]
P06239	LCK	Tyrosine-protein kinase Lck	+	+	2	0	[1], [5]
Q9H400	LIME1	Lck-interacting transmembrane adapter 1	+	+	3	1	-
Q9BU23	LMF2	Lipase maturation factor 2	-	+	0	7	-
Q8IWU2	LMTK2	Serine/threonine-protein kinase LMTK2	+	+	2	3	-
Q9UIQ6-3	LNPEP	Leucyl-cystinyl aminopeptidase (Isoform 3)	+	-	0	1	-
Q9UIQ6	LNPEP	Leucyl-cystinyl aminopeptidase	-	+	0	1	[1], [2], [3]
Q9Y561	LRP12	Low-density lipoprotein receptor-related protein 12	+	-	3	1	-
Q9BTT6	LRRC1	Leucine-rich repeat-containing protein 1	+	-	2	0	[1]
A6NHZ5	LRRC14B	Leucine-rich repeat-containing protein 14B	-	+	0	0	-

UniProt Accession Number	Gene Name	Protein	Enriched in Jurkat T cells	Enriched in Primary T cells	Predicted Palmitoylation Sites[a]	Predicted Transmembrane Helices[b]	Literature References[c]
Q8N386	LRRC25	Leucine-rich repeat-containing protein 25	-	+	0	1	-
Q86X29	LSR	Lipolysis-stimulated lipoprotein receptor	-	+	12	1	-
Q9HBG7	LY9	T-lymphocyte surface antigen Ly-9 (Fragment)	-	+	3	1	[3]
P20645	M6PR	Cation-dependent mannose-6-phosphate receptor	+	+	2	1	[2], [4]
Q9UKM7	MAN1B1	Endoplasmic reticulum mannosyl-oligosaccharide 1,2-alpha-mannosidase	+	-	1	1	-
Q68D91	MBLAC2	Metallo-beta-lactamase domain-containing protein 2	+	+	0	0	[2], [6]
Q9GZU1	MCOLN1	Mucolipin-1	+	-	7	5	-
Q9P1T7	MDFIC	MyoD family inhibitor domain-containing protein	-	+	11	0	-
Q9NU22	MDN1	Midasin	-	+	4	0	[1]
Q6ZSS7	MFSD6	Major facilitator superfamily domain-containing protein 6	-	+	0	11	[2]
Q29980	MICB	MHC class I polypeptide-related sequence B	+	-	0	0	-
Q14165	MLEC	Malectin	-	+	2	1	[1], [2], [3]
Q8IVH4	MMAA	Methylmalonic aciduria type A protein, mitochondrial	+	-	0	0	-
Q9BRT2	MNF1	Mitochondrial nucleoid factor 1	+	+	0	0	[4], [5]
P05164	MPO	Myeloperoxidase	+	-	0	0	-
Q9NZW5	MPP6	MAGUK p55 subfamily member 6	-	+	0	0	[1], [4], [6]
Q8N565	MREG	Melanoregulin	+	+	6	0	[1]
P11836	MS4A1	B-lymphocyte antigen CD20	-	+	1	4	-
Q9Y3D2	MSRB2	Methionine-R-sulfoxide reductase B2, mitochondrial	+	-	0	0	-
P03915	MT-ND5	NADH-ubiquinone oxidoreductase chain 5	-	+	0	15	-
Q86UE4	MTDH	Protein LYRIC	+	+	1	1	[1], [2], [3], [5], [8]
Q13614	MTMR2	Myotubularin-related protein 2	-	+	1	0	-
O75570	MTRF1	Peptide chain release factor 1, mitochondrial	+	-	1	0	-
Q96S97	MYADM	Myeloid-associated differentiation marker	+	+	0	8	[1]
Q9NZM1	MYOF	Myoferlin	-	+	0	1	[3], [4], [5]
P18440	NAT1	Arylamine N-acetyltransferase 1	-	+	0	0	-
Q6PIU2	NCEH1	Neutral cholesterol ester hydrolase 1	-	+	1	1	[1]
Q92542	NCSTN	Nicastrin	+	+	1	1	-
O14561	NDUFAB1	Acyl carrier protein	+	-	0	0	-
Q00653	NFKB2	Nuclear factor NF-kappa-B p100 subunit	-	+	1	0	-
O15118	NPC1	Niemann-Pick C1 protein	+	+	1	12	[3]
P01111	NRAS	GTPase NRas	+	+	2	0	[2], [3], [5]
Q96D31	ORAI1	Calcium release-activated calcium channel protein 1	-	+	0	3	[2]
Q9NWU1	OXSM	3-oxoacyl-[acyl-carrier-protein] synthase, mitochondrial	+	+	2	0	[2], [4], [5]
Q99572	P2RX7	P2X purinoceptor 7	-	+	5	1	[8]
Q9UKS6	PACSIN3	Protein kinase C and casein kinase substrate in neurons protein 3	+	-	2	0	-
Q9NWQ8	PAG1	Phosphoprotein associated with glycosphingolipid-enriched microdomains 1	+	+	2	1	-
Q9H6A9	PCNXL3	Pecanex-like protein 3	-	+	1	11	-
O00330	PDHX	Pyruvate dehydrogenase protein X component, mitochondrial	+	+	0	0	-
P16284	PECAM1	Platelet endothelial cell adhesion molecule	+	+	1	1	[5]
Q9UPP1	PHF8	Histone lysine demethylase PHF8	-	+	1	0	-
Q9BTU6	PI4K2A	Phosphatidylinositol 4-kinase type 2-alpha	+	+	3	0	[1], [2], [3], [5]
Q8TCG2	PI4K2B	Phosphatidylinositol 4-kinase type 2-beta	+	+	4	0	[1], [2]
Q99569	PKP4	Plakophilin-4	+	-	0	0	[8]
O15162	PLSCR1	Phospholipid scramblase 1	+	+	7	0	[2], [3], [4]
Q9NRY6	PLSCR3	Phospholipid scramblase 3	+	+	7	0	[2], [3], [4], [5]
O15031	PLXNB2	Plexin-B2	-	+	1	0	[8]
O00592	PODXL	Podocalyxin	+	-	3	1	[5]
A8CG34	POM121C	Nuclear envelope pore membrane protein POM 121C	+	+	0	1	-
Q96T49	PPP1R16B	Protein phosphatase 1 regulatory inhibitor subunit 16B	-	+	2	0	-
P55345	PRMT2	Protein arginine N-methyltransferase 2	-	+	0	0	-
P49768	PSEN1	Presenilin-1	+	-	0	9	[1]
Q9UNM6	PSMD13	26S proteasome non-ATPase regulatory subunit 13	-	+	0	0	[1]
Q92530	PSMF1	Proteasome inhibitor PI31 subunit	-	+	0	0	-
O43586	PSTPIP1	Proline-serine-threonine phosphatase-interacting protein 1	-	+	0	0	-
Q13308	PTK7	Inactive tyrosine-protein kinase 7	+	-	2	2	[1]
Q9P035	PTPLAD1	Very-long-chain (3R)-3-hydroxyacyl-[acyl-carrier protein] dehydratase 3	-	+	0	5	-
P15151-3	PVR	Poliovirus receptor	+	-	0	0	-
P11234	RALB	Ras-related protein Ral-B	+	-	2	0	[5], [8]
P10114	RAP2A	Ras-related protein Rap-2a	-	+	2	0	[1], [2]
P61225	RAP2B	Ras-related protein Rap-2b	+	+	3	0	[1], [2], [3], [5]
Q9Y3L5	RAP2C	Ras-related protein Rap-2c	+	+	3	0	[1], [2], [4], [5]
Q00765	REEP5	Receptor expression-enhancing protein 5	+	+	0	2	[5], [8]
Q8IUW5	RELL1	RELT-like protein 1	+	+	1	2	[6]
Q8WZ73	RFFL	E3 ubiquitin-protein ligase rififylin	-	+	4	0	-
Q14699	RFTN1	Raftlin	+	+	1	0	[1], [2]
P22670	RFX1	MHC class II regulatory factor RFX1	+	+	0	0	[1], [5]
P49795	RGS19	Regulator of G-protein signaling 19	+	-	9	0	[1], [2]
Q6NTF9	RHBDD2	Rhomboid domain-containing protein 2	+	-	2	5	[1]
Q9Y3C5	RNF11	RING finger protein 11	-	+	1	0	[2]
O75695	RP2	Protein XRP2	+	-	2	0	-
P10301	RRAS	Ras-related protein R-Ras	+	+	2	0	[1], [2], [3], [5], [8]
P62070	RRAS2	Ras-related protein R-Ras2	+	+	2	0	[5], [8]
Q96DX4	RSPRY1	RING finger and SPRY domain-containing protein 1	+	-	3	0	[1]
Q9NQC3	RTN4	Reticulon-4	-	+	0	2	[1], [5]
O95977	S1PR4	Sphingosine 1-phosphate receptor 4	-	+	3	7	-
Q99590	SCAF11	Protein SCAF11	+	-	3	0	-
O15126	SCAMP1	Secretory carrier-associated membrane protein 1	-	+	0	4	[1], [2], [3], [8]
O15127	SCAMP2	Secretory carrier-associated membrane protein 2	+	+	0	4	[1], [2], [3], [8]
O14828	SCAMP3	Secretory carrier-associated membrane protein 3	+	+	0	4	[1], [2], [3], [8]
Q969E2	SCAMP4	Secretory carrier-associated membrane protein 4	-	+	0	4	[2]
Q8WTV0	SCARB1	Scavenger receptor class B member 1	+	-	1	2	[3]
Q14108	SCARB2	Lysosome membrane protein 2	+	+	3	2	[5], [8]
Q14160	SCRIB	Protein scribble homolog	+	+	1	0	[1], [4]
Q9H190	SDCBP2	Syntenin-2	-	+	0	0	-
Q6IQ49-3	SDE2	Protein SDE2 homolog	+	-	1	0	-
Q92854	SEMA4D	Semaphorin-4D	+	+	2	2	[1], [2], [8]
Q9NVA2	SEPT11	Septin-11	-	+	0	0	-
Q9NRX5	SERINC1	Serine incorporator 1	+	+	5	11	-
Q587I9	SFT2D3	Vesicle transport protein SFT2C	+	-	1	4	[1], [2], [4]
Q8NBJ9	SIDT2	SID1 transmembrane family member 2	-	+	2	9	[8]
Q6IA17	SIGIRR	Single Ig IL-1-related receptor	+	+	2	1	-
Q8N697	SLC15A4	Solute carrier family 15 member 4	-	+	2	13	-
O15427	SLC16A3	Monocarboxylate transporter 4 (Fragment)	-	+	1	12	-
P41440	SLC19A1	Folate transporter 1	+	-	0	11	-
Q15758	SLC1A5	Neutral amino acid transporter B(0)	+	+	0	9	[1], [3], [4], [5], [7]
Q6NXT4	SLC30A6	Zinc transporter 6	-	+	3	6	-
Q8TB61-3	SLC35B2	Adenosine 3-phospho 5-phosphosulfate transporter 1	+	-	0	7	-
Q8TB61	SLC35B2	Adenosine 3'-phospho 5'-phosphosulfate transporter 1	-	+	0	8	[1], [3], [4]
Q96K37	SLC35E1	Solute carrier family 35 member E1	-	+	0	6	-
P0CK96	SLC35E2B	Solute carrier family 35 member E2B	-	+	1	8	-
Q96QD8	SLC38A2	Sodium-coupled neutral amino acid transporter 2	+	-	0	9	[1], [6]

UniProt Accession Number	Gene Name	Protein	Enriched in Jurkat T cells	Enriched in Primary T cells	Predicted Palmitoylation Sites[a]	Predicted Transmembrane Helices[b]	Literature References[c]
Q9ULF5	SLC39A10	Zinc transporter ZIP10	+	-	2	7	[8]
Q96GZ6	SLC41A3	Solute carrier family 41 member 3	-	+	0	10	-
Q8NBI5	SLC43A3	Solute carrier family 43 member 3	+	-	1	11	[1], [2]
Q8WWI5	SLC44A1	Choline transporter-like protein 1	+	+	9	9	[2], [3], [8]
Q8IWA5	SLC44A2	Choline transporter-like protein 2	-	+	6	11	[2], [8]
P30825	SLC7A1	High affinity cationic amino acid transporter 1	+	-	2	14	[1], [4], [8]
Q92581-3	SLC9A6	Sodium/hydrogen exchanger 6	+	-	1	11	-
Q92581-2	SLC9A6	Isoform 2 of Sodium/hydrogen exchanger 6	-	+	1	12	-
Q8IVB4	SLC9A9	Sodium/hydrogen exchanger 9	-	+	0	10	[2]
Q9UIG8	SLCO3A1	Solute carrier organic anion transporter family member 3A1	-	+	2	12	-
B2RUZ4	SMIM1	Small integral membrane protein 1	-	+	1	1	-
Q9NY59	SMPD3	Sphingomyelin phosphodiesterase 3	-	+	6	2	-
O00161	SNAP23	Synaptosomal-associated protein 23	+	+	6	0	[1], [2], [3], [4], [5], [6], [7]
P60880-2	SNAP25	Synaptosomal-associated protein 25	+	-	4	0	-
Q9Y5W9	SNX11	Sorting nexin-11	+	-	2	0	-
Q99523	SORT1	Sortilin	+	+	1	1	[1], [2], [8]
Q8TCT8	SPPL2A	Signal peptide peptidase-like 2A	-	+	1	9	-
Q7Z698	SPRED2	Sprouty-related, EVH1 domain-containing protein 2	+	-	3	0	[8]
O43609	SPRY1	Protein sprouty homolog 1	+	-	1	0	-
Q5W111	SPRYD7	SPRY domain-containing protein 7	+	-	2	0	[4]
Q9NUV7	SPTLC3	Serine palmitoyltransferase 3 (Fragment)	-	+	0	0	-
O95772	STARD3NL	MLN64 N-terminal domain homolog	-	+	0	4	[2]
O75716	STK16	Serine/threonine-protein kinase 16	-	+	0	0	-
Q9NZ72	STMN3	Stathmin	+	-	1	0	-
P27105	STOM	Erythrocyte band 7 integral membrane protein	-	+	2	1	[1], [2], [3], [4], [5]
O60499	STX10	Syntaxin-10	+	-	1	1	[1], [2]
O75558	STX11	Syntaxin-11	+	+	6	0	[2], [5]
Q86Y82	STX12	Syntaxin-12	+	+	1	1	[1], [2], [3], [5], [8]
P32856-3	STX2	Syntaxin-2	+	-	0	0	-
Q12846	STX4	Syntaxin-4	+	-	1	1	[1]
O43752	STX6	Syntaxin-6	+	+	1	1	[1], [3], [5], [8]
O15400	STX7	Syntaxin-7	+	+	1	1	[1], [3], [8]
Q9UNK0	STX8	Syntaxin-8	+	+	1	1	[1], [2], [3], [8]
O15260	SURF4	Surfeit locus protein 4	-	+	0	5	[1], [8]
Q96L08	SUSD3	Sushi domain-containing protein 3	-	+	2	1	[2]
Q8NHG7	SVIP	Small VCP/p97-interacting protein	+	-	1	0	[2], [5]
Q6ZMZ3	SYNE3	Nesprin-3	-	+	1	1	-
Q6NXT6	TAPT1	Transmembrane anterior posterior transformation protein 1 homolog	-	+	1	5	-
Q96BS2	TESC	Calcineurin B homologous protein 3	+	+	1	0	-
P02787	TF	Serotransferrin	+	-	2	0	-
Q9H5Q4	TFB2M	Dimethyladenosine transferase 2, mitochondrial	+	-	0	0	-
P02786	TFRC	Transferrin receptor protein 1	-	+	0	1	[1], [2], [5]
Q8WUY1	THEM6	Protein THEM6	-	+	0	1	-
Q99805	TM9SF2	Transmembrane 9 superfamily member 2	-	+	0	9	[6], [8]
Q7Z403	TMC6	Transmembrane channel-like protein 6	-	+	0	10	-
Q8IU68	TMC8	Transmembrane channel-like protein 8	-	+	1	8	-
Q13445	TMED1	Transmembrane emp24 domain-containing protein 1	+	+	1	1	[1]
Q9H6X4	TMEM134	Transmembrane protein 134	+	+	2	2	-
Q7Z7N9	TMEM179B	Transmembrane protein 179B	-	+	2	3	-
Q8IY95	TMEM192	Transmembrane protein 192	+	+	1	4	-
Q9H813	TMEM206	Transmembrane protein 206	+	-	0	1	[1]
Q9H0R3	TMEM222	Transmembrane protein 222	-	+	1	3	[1], [2]
Q9H330	TMEM245	Transmembrane protein 245	-	+	2	14	-
Q86T03	TMEM55B	Transmembrane protein 55B	-	+	1	2	[1], [2], [3], [8]
O94886	TMEM63A	Transmembrane protein 63A	+	+	4	11	[1], [2]
Q8NBN3	TMEM87A	Transmembrane protein 87A	-	+	0	7	-
Q96BF3	TMIGD2	Transmembrane and immunoglobulin domain-containing protein 2	+	+	0	1	-
Q6ZT21	TMPPE	Transmembrane protein with metallophosphoesterase domain	-	+	1	5	[2]
Q9H3N1	TMX1	Thioredoxin-related transmembrane protein 1	+	+	0	3	[1], [2], [3], [5], [7], [8]
Q96JJ7	TMX3	Protein disulfide-isomerase TMX3	+	+	1	1	[1], [2]
Q9H1E5	TMX4	Thioredoxin-related transmembrane protein 4	+	+	1	1	[1], [2]
P28908	TNFRSF8	Tumor necrosis factor receptor superfamily member 8	+	-	1	1	-
O43557	TNFSF14	Tumor necrosis factor ligand superfamily member 14	-	+	0	1	-
Q9Y228	TRAF3IP3	TRAF3-interacting JNK-activating modulator	+	+	0	1	[1]
O43617	TRAPPC3	Trafficking protein particle complex subunit 3	+	-	1	0	[2], [3], [5]
Q86YW5	TREML1	Trem-like transcript 1 protein	-	+	0	1	-
P49815	TSC2	Tuberin	-	+	0	0	-
Q8NG11	TSPAN14	Tetraspanin-14	+	-	1	4	[2], [6]
O95858	TSPAN15	Tetraspanin-15	+	-	3	4	[2]
Q96SJ8	TSPAN18	Tetraspanin-18 (Fragment)	-	+	6	4	-
Q86UF1	TSPAN33	Tetraspanin-33	+	+	3	4	[2]
P41732	TSPAN7	Tetraspanin-7	+	-	4	4	-
O75954	TSPAN9	Tetraspanin-9	-	+	4	4	[2]
Q8NFU3	TSTD1	Thiosulfate sulfurtransferase/rhodanese-like domain-containing protein 1	+	-	0	0	-
Q9C0H2	TTYH3	Protein tweety homolog 3	-	+	3	5	[2], [8]
Q9Y3C8	UFC1	Ubiquitin-fold modifier-conjugating enzyme 1	+	-	1	0	-
O75385	ULK1	Serine/threonine-protein kinase ULK1	-	+	3	0	-
P63027	VAMP2	Vesicle-associated membrane protein 2	-	+	0	1	[1], [5], [8]
Q15836	VAMP3	Vesicle-associated membrane protein 3	+	+	0	1	[1], [2], [3], [5]
O75379	VAMP4	Vesicle-associated membrane protein 4	+	+	0	1	[1], [2], [3], [8]
O95183	VAMP5	Vesicle-associated membrane protein 5	+	-	0	1	[1], [2], [3]
Q8IUH4	ZDHHC13	Palmitoyltransferase ZDHHC13	+	-	1	6	-
Q8IUH5	ZDHHC17	Palmitoyltransferase ZDHHC17	+	+	0	6	[1]
Q9NUE0	ZDHHC18	Palmitoyltransferase ZDHHC18	-	+	2	4	-
Q5W0Z9	ZDHHC20	Probable palmitoyltransferase ZDHHC20	-	+	2	4	[1]
Q9C0B5	ZDHHC5	Palmitoyltransferase ZDHHC5	+	+	2	4	[1], [4]

Supplementary Table 4
The "known" palmitome, proteins quantified in our studies and identified as palmitoylated in at least two published proteomic studies. "Known" palmitoylated proteins we identified as unpalmitoylated are shown in yellow.

[a]Palmitoylation sites predicted using CSS-Palm (version 3.0), with a “High” threshold and a cutoff score of 1.0. [b]Prediction of transmembrane domains was performed using TMHMM Server (version 2.0). [c]References: [1] Martin and Cravatt, Nat. Meth., 2009, [2] Dowal et al., Blood, 2011, [3] Merrick et al., Mol. & Cell. Proteom., 2011, [4] Yang et al., Mol. & Cell. Proteom., 2010, [5] Marin et al., Circ. Res., 2012, [6] Forrester et al., J. Lip. Res., 2011, [7] Wilson et al., Mol. & Cell. Proteom., 2011, [8] Kang et al., Nature, 2008.

UniProt Accession Number	Gene Name	Protein	Enriched in Jurkat T cells	Enriched in Primary T cells	Predicted Palmitoylation Sites[a]	Predicted Transmembrane Helices[b]	Literature References[c]
O95870	ABHD16A	Abhydrolase domain-containing protein 16A	-	+	1	2	[1], [8]
Q5VST6	ABHD17B	Alpha/beta hydrolase domain-containing protein 17B	+	+	4	0	[1], [3], [7]
P68133	ACTA1	Actin, alpha skeletal muscle	-	-	1	0	[1], [7]
P60709	ACTB	Actin, cytoplasmic 1	-	-	1	0	[4], [5], [7]
O43707	ACTN4	Alpha-actinin-4	-	+	0	0	[4], [5]
O14672	ADAM10	Disintegrin and metalloproteinase domain-containing protein 10	-	+	2	1	[1], [8]
O43306	ADCY6	Adenylate cyclase type 6	-	+	0	12	[2], [8]
Q9Y4W6	AFG3L2	AFG3-like protein 2	-	-	2	2	[2], [8]
Q99943	AGPAT1	1-acyl-sn-glycerol-3-phosphate acyltransferase alpha	+	-	0	3	[1], [3], [8]
Q09666	AHNAK	Neuroblast differentiation-associated protein AHNAK	-	-	0	0	[4], [5]
P04075	ALDOA	Fructose-bisphosphate aldolase A	-	-	0	0	[1], [6]
P84077	ARF1	ADP-ribosylation factor 1	-	-	0	0	[1], [7]
P18085	ARF4	ADP-ribosylation factor 4	-	-	0	0	[5], [7], [8]
P84085	ARF5	ADP-ribosylation factor 5	-	-	0	0	[2], [7], [8]
Q9NXU5	ARL15	ADP-ribosylation factor-like protein 15	+	+	0	0	[1], [2], [3], [8]
Q13510	ASAH1	Acid ceramidase	-	-	1	0	[2], [8]
Q9Y2G3	ATP11B	Probable phospholipid-transporting ATPase IF	+	+	4	8	[1], [2]
P05023	ATP1A1	Sodium/potassium-transporting ATPase subunit alpha-1	-	-	0	10	[1], [2], [4], [6]
P25705	ATP5A1	ATP synthase subunit alpha, mitochondrial	-	-	0	0	[5], [7]
P36542	ATP5C1	ATP synthase subunit gamma, mitochondrial	-	-	0	0	[1], [6]
P48047	ATP5O	ATP synthase subunit O, mitochondrial	-	-	0	0	[1], [8]
Q9Y487	ATP6V0A2	V-type proton ATPase 116 kDa subunit a isoform 2	+	+	1	6	[1], [2]
Q07021	C1QBP	Complement component 1 Q subcomponent-binding protein, mitochondrial	-	-	0	0	[2], [5], [7]
P27824	CANX	Calnexin	+	+	2	1	[1], [3], [5], [7], [8]
O15484	CAPN5	Calpain-5	-	+	1	0	[4], [5], [8]
Q8ND76	CCNY	Cyclin-Y	+	+	2	0	[1], [2], [3]
P78371	CCT2	T-complex protein 1 subunit beta	-	-	1	0	[1], [7]
P48643	CCT5	T-complex protein 1 subunit epsilon	-	-	0	0	[1], [5], [7]
P40227	CCT6A	T-complex protein 1 subunit zeta	-	-	0	0	[5], [7]
P50990	CCT8	T-complex protein 1 subunit theta	-	-	1	0	[5], [7]
P48509	CD151	CD151 antigen	-	+	7	4	[2], [6]
P16671	CD36	Platelet glycoprotein 4 (Fragment)	-	+	4	2	[2], [3]
P28907	CD38	ADP-ribosyl cyclase 1	+	+	2	1	[1], [2], [4], [8]
P16070	CD44	CD44 antigen (Fragment)	-	+	2	1	[3], [5]
P08962	CD63	CD63 antigen	+	+	7	4	[2], [5]
P27701	CD82	CD82 antigen	+	+	5	4	[1], [2], [5], [7]
P14209	CD99	CD99 antigen	+	+	0	2	[5], [6]
Q8TCZ2	CD99L2	CD99 antigen-like protein 2	-	+	1	1	[2], [8]
P60953	CDC42	Cell division control protein 42 homolog	-	-	2	0	[2], [8]
Q9NRR3	CDC42SE2	CDC42 small effector protein 2	-	-	3	0	[1], [2]
P23528	CFL1	Cofilin-1	-	-	0	0	[5], [7]
Q07065	CKAP4	Cytoskeleton-associated protein 4	+	+	1	1	[3], [4], [5]
Q00610	CLTC	Clathrin heavy chain 1	-	-	0	0	[1], [7]
Q9ULV4	CORO1C	Coronin-1C	-	-	0	0	[4], [8]
O75976	CPD	Carboxypeptidase D	-	-	3	1	[1], [8]
Q9HCP0	CSNK1G1	Casein kinase I isoform gamma-1	+	+	3	0	[1], [2], [8]
Q9Y6M4	CSNK1G3	Casein kinase I isoform gamma-3	-	+	3	0	[1], [2], [8]
O43169	CYB5B	Cytochrome b5 type B	-	-	0	1	[2], [5], [7], [8]
P00387	CYB5R3	NADH-cytochrome b5 reductase 3	-	-	0	0	[1], [8]
P99999	CYCS	Cytochrome c	-	-	0	0	[6], [8]
Q9Y4D1	DAAM1	Disheveled-associated activator of morphogenesis 1	+	+	1	0	[1], [2], [8]
Q14118	DAG1	Dystroglycan	-	-	2	1	[2], [5], [8]
Q8NCG7	DAGLB	Sn1-specific diacylglycerol lipase beta	+	+	3	4	[1], [2], [3]
Q08211	DHX9	ATP-dependent RNA helicase A	-	-	1	0	[4], [7]
Q9NVH1	DNAJC11	DnaJ homolog subfamily C member 11	-	-	0	0	[1], [8]
Q9H3Z4	DNAJC5	DnaJ homolog subfamily C member 5	+	+	13	1	[1], [2], [3]
Q16555	DPYSL2	Dihydropyrimidinase-related protein 2	-	-	0	0	[4], [5]
O00559	EBAG9	Receptor-binding cancer antigen expressed on SiSo cells	+	-	1	0	[1], [2], [8]
P42892	ECE1	Endothelin-converting enzyme 1	+	+	0	1	[2], [5]
P68104	EEF1A1	Elongation factor 1-alpha 1	-	-	0	0	[7], [8]
P26641	EEF1G	Elongation factor 1-gamma	-	-	0	0	[1], [6], [8]
P13639	EEF2	Elongation factor 2	-	-	1	0	[1], [5], [7]
Q14156	EFR3A	Protein EFR3 homolog A	-	+	4	0	[2], [3]
P63241	EIF5A	Eukaryotic translation initiation factor 5A-1	-	-	0	0	[1], [2], [6]
P06733	ENO1	Alpha-enolase	-	-	0	0	[5], [7]
P07814	EPRS	Bifunctional glutamate/proline--tRNA ligase	-	-	1	0	[1], [8]
Q96RT1	ERBB2IP	Protein LAP2	-	+	3	0	[1], [2]
Q9Y282	ERGIC3	Endoplasmic reticulum-Golgi intermediate compartment protein 3	+	+	0	2	[1], [2], [3], [5], [8]
Q9BSJ8	ESYT1	Extended synaptotagmin-1	-	-	0	2	[1], [6]
Q9NUQ9	FAM49B	Protein FAM49B	+	+	0	0	[1], [2]
P49327	FASN	Fatty acid synthase	-	-	1	0	[5], [6]
P21333	FLNA	Filamin-A	-	-	0	0	[1], [4]
O75369	FLNB	Filamin-B	-	-	0	0	[1], [4], [5]
O75955	FLOT1	Flotillin-1	+	+	1	0	[1], [2], [3], [4], [5]
Q14254	FLOT2	Flotillin-2	+	+	3	0	[1], [2], [3], [5]
P06241	FYN	Tyrosine-protein kinase Fyn	-	+	4	0	[1], [2], [3], [5]
P04406	GAPDH	Glyceraldehyde-3-phosphate dehydrogenase	-	-	1	0	[4], [6], [7]
Q92896	GLG1	Golgi apparatus protein 1	-	-	2	1	[1], [3], [8]
P29992	GNA11	Guanine nucleotide-binding protein subunit alpha-11	-	+	2	0	[1], [2], [4], [5], [6]
Q14344	GNA13	Guanine nucleotide-binding protein subunit alpha-13	+	+	2	0	[1], [2], [4], [5]
P30679	GNA15	Guanine nucleotide-binding protein subunit alpha-15	+	-	3	0	[1], [2]
P50148	GNAQ	Guanine nucleotide-binding protein G(q) subunit alpha	+	+	2	0	[1], [2], [3], [4], [5]
P63244	GNB2L1	Guanine nucleotide-binding protein subunit beta-2-like 1	-	-	0	0	[5], [7]
Q7Z5G4	GOLGA7	Golgin subfamily A member 7	+	+	0	0	[1], [2], [3]
P09211	GSTP1	Glutathione S-transferase P	-	-	0	0	[1], [7]
P40939	HADHA	Trifunctional enzyme subunit alpha, mitochondrial	-	-	1	0	[1], [8]
P01889	HLA-B	HLA class I histocompatibility antigen, B-7 alpha chain	+	-	2	1	[1], [4]
P09651	HNRNPA1	Heterogeneous nuclear ribonucleoprotein A1	-	-	0	0	[5], [7], [8]
P22626	HNRNPA2B1	Heterogeneous nuclear ribonucleoproteins A2/B1	-	-	0	0	[5], [7], [8]
P61978	HNRNPK	Heterogeneous nuclear ribonucleoprotein K	-	-	0	0	[5], [7]
Q00839	HNRNPU	Heterogeneous nuclear ribonucleoprotein U	-	-	0	0	[7], [8]
P01112	HRAS	GTPase HRas	+	-	3	0	[1], [2], [5], [6]
Q99714	HSD17B10	3-hydroxyacyl-CoA dehydrogenase type-2	-	-	1	0	[1], [7]
Q53GQ0	HSD17B12	Estradiol 17-beta-dehydrogenase 12	-	-	1	3	[3], [8]
P08107	HSPA1A	Heat shock 70 kDa protein 1A/1B	-	-	0	0	[1], [7]

UniProt Accession Number	Gene Name	Protein	Enriched in Jurkat T cells	Enriched in Primary T cells	Predicted Palmitoylation Sites[a]	Predicted Transmembrane Helices[b]	Literature References[c]
P38646	HSPA9	Stress-70 protein, mitochondrial	-	-	0	0	[6], [7]
P10809	HSPD1	60 kDa heat shock protein, mitochondrial	-	-	0	0	[5], [7]
Q01629	IFITM2	Interferon-induced transmembrane protein 1	+	-	3	2	[3], [5]
Q01628	IFITM3	Interferon-induced transmembrane protein 3	-	+	3	2	[2], [3], [5]
P11717	IGF2R	Cation-independent mannose-6-phosphate receptor	+	-	3	1	[1], [3], [7], [8]
Q969P0	IGSF8	Immunoglobulin superfamily member 8	+	+	2	1	[1], [8]
Q14642	INPP5A	Type I inositol 1,4,5-trisphosphate 5-phosphatase	-	-	1	0	[2], [8]
O00410	IPO5	Importin-5	-	-	2	0	[1], [7], [8]
Q9BX67	JAM3	Junctional adhesion molecule C	+	+	3	1	[2], [8]
P14923	JUP	Junction plakoglobin	-	-	0	0	[4], [5]
Q8IYS2	KIAA2013	Uncharacterized protein KIAA2013	+	+	0	2	[1], [2], [3]
Q14974	KPNB1	Importin subunit beta-1	-	-	2	0	[1], [7]
Q6IAA8	LAMTOR1	Ragulator complex protein LAMTOR1	+	-	2	0	[1], [5], [7]
O43561	LAT	Linker for activation of T-cells family member 1	+	+	3	1	[1], [2], [3]
P06239	LCK	Tyrosine-protein kinase Lck	+	+	2	0	[1], [5]
P07195	LDHB	L-lactate dehydrogenase B chain	-	-	0	0	[5], [7], [8]
Q9UIQ6	LNPEP	Leucyl-cystinyl aminopeptidase	-	+	0	1	[1], [2], [3]
Q8NF37	LPCAT1	Lysophosphatidylcholine acyltransferase 1	-	-	1	1	[2], [8]
P20645	M6PR	Cation-dependent mannose-6-phosphate receptor	+	+	2	1	[2], [4]
Q68D91	MBLAC2	Metallo-beta-lactamase domain-containing protein 2	+	+	0	0	[2], [6]
P43121	MCAM	Cell surface glycoprotein MUC18	-	-	3	1	[5], [8]
P40926	MDH2	Malate dehydrogenase, mitochondrial	-	-	2	0	[5], [7]
O14880	MGST3	Microsomal glutathione S-transferase 3	-	-	2	4	[1], [6]
Q14165	MLEC	Malectin	-	+	2	1	[1], [2], [3]
Q9BRT2	MNF1	Mitochondrial nucleoid factor 1	+	+	0	0	[4], [5]
Q00013	MPP1	55 kDa erythrocyte membrane protein	-	-	1	0	[2], [4]
Q9NZW5	MPP6	MAGUK p55 subfamily member 6	-	+	0	0	[1], [4], [6]
P25325	MPST	3-mercaptopyruvate sulfurtransferase	-	-	0	0	[2], [8]
Q86UE4	MTDH	Protein LYRIC	+	+	1	1	[1], [2], [3], [5], [8]
P35579	MYH9	Myosin-9	-	-	0	0	[7], [8]
B0I1T2	MYO1G	Unconventional myosin-Ig	-	-	0	0	[1], [7]
Q9NZM1	MYOF	Myoferlin	-	+	0	1	[3], [4], [5]
Q9BT67	NDFIP1	NEDD4 family-interacting protein 1	-	-	1	3	[1], [4]
Q13423	NNT	NAD(P) transhydrogenase, mitochondrial	-	-	1	12	[1], [2], [8]
Q15233	NONO	Non-POU domain-containing octamer-binding protein	-	-	0	0	[1], [4]
P06748	NPM1	Nucleophosmin	-	-	0	0	[7], [8]
P01111	NRAS	GTPase NRas	+	+	2	0	[2], [3], [5]
Q9NWU1	OXSM	3-oxoacyl-[acyl-carrier-protein] synthase, mitochondrial	+	+	2	0	[2], [4], [5]
P07237	P4HB	Protein disulfide-isomerase	-	-	1	0	[6], [7]
P07737	PFN1	Profilin-1	-	-	0	0	[5], [7], [8]
Q9BTU6	PI4K2A	Phosphatidylinositol 4-kinase type 2-alpha	+	+	3	0	[1], [2], [3], [5]
Q8TCG2	PI4K2B	Phosphatidylinositol 4-kinase type 2-beta	+	+	4	0	[1], [2]
P14618	PKM	Pyruvate kinase isozymes M1/M2	-	-	2	0	[5], [7]
Q04941	PLP2	Proteolipid protein 2	-	-	0	4	[2], [4]
O15162	PLSCR1	Phospholipid scramblase 1	+	+	7	0	[2], [3], [4]
Q9NRY6	PLSCR3	Phospholipid scramblase 3	+	+	7	0	[2], [3], [4], [5]
P23284	PPIB	Peptidyl-prolyl cis-trans isomerase B	-	-	0	1	[7], [8]
O60831	PRAF2	PRA1 family protein 2	-	-	0	4	[1], [2], [3], [8]
Q06830	PRDX1	Peroxiredoxin-1	-	-	0	0	[7], [8]
P30041	PRDX6	Peroxiredoxin-6	-	-	0	0	[5], [8]
Q9UMS4	PRPF19	Pre-mRNA-processing factor 19	-	-	2	0	[1], [4]
Q9UL46	PSME2	Proteasome activator complex subunit 2	-	-	1	0	[1], [7]
P26599	PTBP1	Polypyrimidine tract-binding protein 1	-	-	2	0	[1], [3]
P53801	PTTG1IP	Pituitary tumor-transforming gene 1 protein-interacting protein	-	-	6	2	[1], [2]
P61026	RAB10	Ras-related protein Rab-10	-	-	2	0	[5], [8]
P62820	RAB1A	Ras-related protein Rab-1A	-	-	2	0	[5], [7]
Q9H0U4	RAB1B	Ras-related protein Rab-1B	-	-	2	0	[1], [5], [7], [8]
P61019	RAB2A	Ras-related protein Rab-2A	-	-	2	0	[5], [8]
P51148	RAB5C	Ras-related protein Rab-5C	-	-	1	0	[1], [8]
P11233	RALA	Ras-related protein Ral-A	-	-	2	0	[3], [5], [8]
P11234	RALB	Ras-related protein Ral-B	+	-	2	0	[5], [8]
P62826	RAN	GTP-binding nuclear protein Ran	-	-	0	0	[5], [7], [8]
P10114	RAP2A	Ras-related protein Rap-2a	-	+	2	0	[1], [2]
P61225	RAP2B	Ras-related protein Rap-2b	+	+	3	0	[1], [2], [3], [5]
Q9Y3L5	RAP2C	Ras-related protein Rap-2c	+	+	3	0	[1], [2], [4], [5]
Q00765	REEP5	Receptor expression-enhancing protein 5	+	+	0	2	[5], [8]
Q14699	RFTN1	Raftlin	+	+	1	0	[1], [2]
P22670	RFX1	MHC class II regulatory factor RFX1	+	+	0	0	[1], [5]
P49795	RGS19	Regulator of G-protein signaling 19	+	-	9	0	[1], [2]
P27635	RPL10	60S ribosomal protein L10	-	-	0	0	[1], [4], [6], [7]
P62906	RPL10A	60S ribosomal protein L10a	-	-	0	0	[1], [4], [6]
P30050	RPL12	60S ribosomal protein L12	-	-	1	0	[1], [4], [5], [7]
P61313	RPL15	60S ribosomal protein L15	-	-	0	0	[6], [8]
Q02543	RPL18A	60S ribosomal protein L18a	-	-	0	0	[1], [4]
P61353	RPL27	60S ribosomal protein L27	-	-	0	0	[3], [8]
P39023	RPL3	60S ribosomal protein L3	-	-	0	0	[1], [3], [8]
P46777	RPL5	60S ribosomal protein L5	-	-	0	0	[1], [8]
Q02878	RPL6	60S ribosomal protein L6	-	-	1	0	[1], [8]
P62917	RPL8	60S ribosomal protein L8	-	-	0	0	[4], [8]
P32969	RPL9	60S ribosomal protein L9	-	-	0	0	[3], [5]
P05388	RPLP0	60S acidic ribosomal protein P0	-	-	0	0	[4], [7]
P04843	RPN1	Dolichyl-diphosphooligosaccharide--protein glycosyltransferase subunit 1	-	-	0	1	[7], [8]
P04844	RPN2	Dolichyl-diphosphooligosaccharide--protein glycosyltransferase subunit 2	-	-	0	4	[3], [8]
P62249	RPS16	40S ribosomal protein S16	-	-	0	0	[1], [6], [7]
P15880	RPS2	40S ribosomal protein S2	-	-	0	0	[1], [4]
P62266	RPS23	40S ribosomal protein S23	-	-	1	0	[1], [6], [8]
P61247	RPS3A	40S ribosomal protein S3a	-	-	0	0	[1], [7]
P46782	RPS5	40S ribosomal protein S5	-	-	1	0	[1], [6]
P62753	RPS6	40S ribosomal protein S6	-	-	1	0	[1], [5]
P10301	RRAS	Ras-related protein R-Ras	+	+	2	0	[1], [2], [3], [5], [8]
P62070	RRAS2	Ras-related protein R-Ras2	+	+	2	0	[5], [8]
O95197	RTN3	Reticulon-3 (Fragment)	-	-	0	3	[4], [8]
Q9NQC3	RTN4	Reticulon-4	-	+	0	2	[1], [5]
Q9Y230	RUVBL2	RuvB-like 2	-	-	0	0	[1], [4]
O15126	SCAMP1	Secretory carrier-associated membrane protein 1	-	+	0	4	[1], [2], [3], [8]
O15127	SCAMP2	Secretory carrier-associated membrane protein 2	+	+	0	4	[1], [2], [3], [8]
O14828	SCAMP3	Secretory carrier-associated membrane protein 3	+	+	0	4	[1], [2], [3], [8]
Q14108	SCARB2	Lysosome membrane protein 2	+	+	3	2	[5], [8]
Q14160	SCRIB	Protein scribble homolog	+	+	1	0	[1], [4]
Q92854	SEMA4D	Semaphorin-4D	+	+	2	2	[1], [2], [8]
Q587I9	SFT2D3	Vesicle transport protein SFT2C	+	-	1	4	[1], [2], [4]
P43007	SLC1A4	Neutral amino acid transporter A	-	-	0	9	[1], [8]

UniProt Accession Number	Gene Name	Protein	Enriched in Jurkat T cells	Enriched in Primary T cells	Predicted Palmitoylation Sites[a]	Predicted Transmembrane Helices[b]	Literature References[c]
Q15758	SLC1A5	Neutral amino acid transporter B(0)	+	+	0	9	[1], [3], [4], [5], [7]
Q00325	SLC25A3	Phosphate carrier protein, mitochondrial	-	-	0	2	[1], [8]
P12236	SLC25A6	ADP/ATP translocase 3	-	-	0	2	[1], [7]
Q8TB61	SLC35B2	Adenosine 3'-phospho 5'-phosphosulfate transporter 1	-	+	0	8	[1], [3], [4]
Q96QD8	SLC38A2	Sodium-coupled neutral amino acid transporter 2	+	-	0	9	[1], [6]
Q8NBI5	SLC43A3	Solute carrier family 43 member 3	+	-	1	11	[1], [2]
Q8WWI5	SLC44A1	Choline transporter-like protein 1	+	+	9	9	[2], [3], [8]
Q8IWA5	SLC44A2	Choline transporter-like protein 2	-	+	6	11	[2], [8]
Q9Y289	SLC5A6	Sodium-dependent multivitamin transporter	-	-	1	14	[1], [4]
P30825	SLC7A1	High affinity cationic amino acid transporter 1	+	-	2	14	[1], [4], [8]
Q92581	SLC9A6	Sodium/hydrogen exchanger 6	-	-	1	12	[1], [8]
O00161	SNAP23	Synaptosomal-associated protein 23	+	+	6	0	[1], [2], [3], [4], [5], [6], [7]
Q99523	SORT1	Sortilin	+	+	1	1	[1], [2], [8]
Q7Z699	SPRED1	Sprouty-related, EVH1 domain-containing protein 1	-	-	4	0	[3], [8]
P27105	STOM	Erythrocyte band 7 integral membrane protein	-	+	2	1	[1], [2], [3], [4], [5]
O60499	STX10	Syntaxin-10	+	-	1	1	[1], [2]
O75558	STX11	Syntaxin-11	+	+	6	0	[2], [5]
Q86Y82	STX12	Syntaxin-12	+	+	1	1	[1], [2], [3], [5], [8]
O43752	STX6	Syntaxin-6	+	+	1	1	[1], [3], [5], [8]
O15400	STX7	Syntaxin-7	+	+	1	1	[1], [3], [8]
Q9UNK0	STX8	Syntaxin-8	+	+	1	1	[1], [2], [3], [8]
O15260	SURF4	Surfeit locus protein 4	-	+	0	5	[1], [8]
Q8NHG7	SVIP	Small VCP/p97-interacting protein	+	-	1	0	[2], [5]
P17987	TCP1	T-complex protein 1 subunit alpha	-	-	0	0	[7], [8]
P02786	TFRC	Transferrin receptor protein 1	-	+	0	1	[1], [2], [5]
Q3ZCQ8	TIMM50	Mitochondrial import inner membrane translocase subunit TIM50	-	-	0	1	[2], [6]
Q99805	TM9SF2	Transmembrane 9 superfamily member 2	-	+	0	9	[6], [8]
Q9H0R3	TMEM222	Transmembrane protein 222	-	+	1	3	[1], [2]
Q86T03	TMEM55B	Transmembrane protein 55B	-	+	1	2	[1], [2], [3], [8]
O94886	TMEM63A	Transmembrane protein 63A	+	+	4	11	[1], [2]
Q5T3F8	TMEM63B	Transmembrane protein 63B	-	-	6	11	[2], [8]
Q9H3N1	TMX1	Thioredoxin-related transmembrane protein 1	+	+	0	3	[1], [2], [3], [5], [7], [8]
Q96JJ7	TMX3	Protein disulfide-isomerase TMX3	+	+	1	1	[1], [2]
Q9H1E5	TMX4	Thioredoxin-related transmembrane protein 4	+	+	1	1	[1], [2]
P06753	TPM3	Tropomyosin alpha-3 chain	-	-	0	0	[4], [5]
O43617	TRAPPC3	Trafficking protein particle complex subunit 3	+	-	1	0	[2], [3], [5]
Q8NG11	TSPAN14	Tetraspanin-14	+	-	1	4	[2], [6]
Q9C0H2	TTYH3	Protein tweety homolog 3	-	+	3	5	[2], [8]
P49411	TUFM	Elongation factor Tu, mitochondrial	-	-	0	0	[1], [6]
P47985	UQCRFS1	Cytochrome b-c1 complex subunit Rieske, mitochondrial	-	-	0	0	[2], [8]
Q96IX5	USMG5	Up-regulated during skeletal muscle growth protein 5	-	-	0	1	[1], [8]
P63027	VAMP2	Vesicle-associated membrane protein 2	-	+	0	1	[1], [5], [8]
Q15836	VAMP3	Vesicle-associated membrane protein 3	+	+	0	1	[1], [2], [3], [5]
O75379	VAMP4	Vesicle-associated membrane protein 4	+	+	0	1	[1], [2], [3], [8]
O95183	VAMP5	Vesicle-associated membrane protein 5	+	-	0	1	[1], [2], [3]
P51809	VAMP7	Vesicle-associated membrane protein 7	-	-	3	1	[1], [2], [3], [8]
Q9P0L0	VAPA	Vesicle-associated membrane protein-associated protein A	-	-	0	1	[1], [4], [8]
P21796	VDAC1	Voltage-dependent anion-selective channel protein 1	-	-	0	0	[7], [8]
P45880	VDAC2	Voltage-dependent anion-selective channel protein 2	-	-	1	0	[5], [7], [8]
Q9Y277	VDAC3	Voltage-dependent anion-selective channel protein 3	-	-	1	0	[5], [7], [8]
O14980	XPO1	Exportin-1	-	-	0	0	[1], [8]
P07947	YES1	Tyrosine-protein kinase Yes	-	-	1	0	[1], [2], [4], [5]
P61981	YWHAG	14-3-3 protein gamma	-	-	1	0	[1], [7]
P63104	YWHAZ	14-3-3 protein zeta/delta	-	-	0	0	[5], [7]
Q9C0B5	ZDHHC5	Palmitoyltransferase ZDHHC5	+	+	2	4	[1], [4]
Q9H6R6	ZDHHC6	Palmitoyltransferase ZDHHC6	-	-	2	4	[1], [4]

Supplementary Table 5

Palmitoylated proteins enriched in Jurkat T cells. Using quantification by SILAC-labeling, Isotopic ratios were measured (n=6). Ratios greater than 1.75 are considered "enriched" (red).

UniProt Accession Number	Gene Name	Protein	Isotopic Ratio (n = 6)					
			Sample 1	Sample 2	Sample 3	Sample 4	Sample 5	Sample 6
Q9NP58	ABCB6	ATP-binding cassette sub-family B member 6, mitochondrial	-	-	-	2.140915027	1.7747	1.53212091
Q96GS6	ABHD17A	Alpha/beta hydrolase domain-containing protein 17A	-	5.311520688	3.1391	4.156275977	-	-
Q5VST6	ABHD17B	Alpha/beta hydrolase domain-containing protein 17B	2.9262	6.153467479	1.4059	16.63561352	1.6064	3.9698293
P09110	ACAA1	3-ketoacyl-CoA thiolase, peroxisomal	2.3294	2.30813618	1.8003	3.696994344	1.1785	1.71007405
P24752	ACAT1	Acetyl-CoA acetyltransferase, mitochondrial	3.3373	3.56518949	1.1095	6.48466377	2.3659	2.37445091
Q9BWD1	ACAT2	Acetyl-CoA acetyltransferase, cytosolic	2.8561	4.046944557	1.0271	7.72797527	4.165	4.3157395
P78536	ADAM17	Disintegrin and metalloproteinase domain-containing protein 17	-	2.088075004	1.369	5.231493591	-	-
P51828	ADCY7	Adenylate cyclase type 7	-	-	-	7.664597225	2.0956	2.47114933
Q99943	AGPAT1	1-acyl-sn-glycerol-3-phosphate acyltransferase alpha	-	-	1.8859	5.386479935	-	0.65006826
Q02252	ALDH6A1	Methylmalonate-semialdehyde dehydrogenase [acylating], mitochondrial	1.68	2.028767929	2.3747	5.391707554	2.5041	2.82877429
Q9H6X2	ANTXR1	Anthrax toxin receptor 1	-	-	2.1977	14.02131239	-	-
Q63HQ0	AP1AR	AP-1 complex-associated regulatory protein	10.219	6.666222252	2.0496	7.183908046	4.9995	1.18890514
Q9NXU5	ARL15	ADP-ribosylation factor-like protein 15	15.177	7.24847782	3.8864	22.51136824	1.6509	3.42641768
Q8N6S5	ARL6IP6	ADP-ribosylation factor-like protein 6-interacting protein 6	-	-	1.9221	5.773005427	0.6546	10.6803375
Q9Y2G3	ATP11B	Probable phospholipid-transporting ATPase IF	-	-	-	6.595870985	2.8025	0.39206461
Q9Y487	ATP6V0A2	V-type proton ATPase 116 kDa subunit a isoform 2	-	-	-	4.488934776	2.0383	1.33445427
O43861	ATP9B	Probable phospholipid-transporting ATPase IIB	-	-	-	-	2.0655	2.10499726
O75882	ATRN	Attractin	-	-	1.7639	6.906077348	-	-
Q9Y679	AUP1	Ancient ubiquitous protein 1	11.099	6.853539853	1.4259	26.32063801	-	-
Q9Y679-3	AUP1	Ancient ubiquitous protein 1	-	-	-	-	1.9961	2.07934792
O43505	B3GNT1	N-acetyllactosaminide beta-1,3-N-acetylglucosaminyltransferase	3.3128	4.107113521	1.5343	7.211885187	-	-
P15291	B4GALT1	Beta-1,4-galactosyltransferase 1	-	2.543623137	1.2587	10.85410991	-	-
Q9UBV7	B4GALT7	Beta-1,4-galactosyltransferase 7	-	-	3.3855	7.171029043	-	-
Q9HB09	BCL2L12	Bcl-2-like protein 12	12.952	3.090999011	2.083	20.92225291	-	0.55423156
Q9NYM9	BET1L	BET1-like protein	4.2814	9.478672986	-	-	-	1.99740338
Q10589	BST2	Bone marrow stromal antigen 2	9.1743	8.336112037	3.2629	25.57283142	4.0551	5.34302201
Q6P1X6	C8orf82	UPF0598 protein C8orf82	2.62	2.582511234	1.7354	5.060728745	1.0035	2.15081516
P27824	CANX	Calnexin	13.681	5.833625015	2.055	20.87159793	1.7796	1.91545195
Q8ND76	CCNY	Cyclin-Y	4.5727	2.956480605	3.0041	25.21177894	-	-
Q8ND76-3	CCNY	Cyclin-Y	-	-	-	-	3.5083	1.81507968
Q8N7R7	CCNYL1	Cyclin-Y-like protein 1	-	-	-	13.58917215	1.8942	-
P29017	CD1C	T-cell surface glycoprotein CD1c	6.8256	6.973500697	5.4573	16.05832383	3.7889	2.10530748
P28907	CD38	ADP-ribosyl cyclase 1	8.0949	6.078288354	2.667	8.276775368	5.2427	2.84438376
P04234	CD3D	T-cell surface glycoprotein CD3 delta chain	10.557	5.145356316	1.5766	39.24800816	0.95407	4.9845479
P06127	CD5	T-cell surface glycoprotein CD5	4.8468	1.72884755	-	6.386511687	0.88502	1.86992782
P19397	CD53	Leukocyte surface antigen CD53	-	-	2.3475	-	2.2516	1.21256214
P08962	CD63	CD63 antigen	-	1.406766547	4.5156	8.888098836	-	-
P09564	CD7	T-cell antigen CD7	7.3952	3.811847221	-	24.53265296	1.6241	2.22138303
P27701	CD82	CD82 antigen	5.1479	1.846278825	1.7477	6.886103842	2.0204	3.27300101
P48960-2	CD97	CD97 antigen	4.5209	-	-	7.670476337	-	1.52070439
P14209	CD99	CD99 antigen	18.836	8.619203586	1.9123	21.52898878	-	10.1422964
Q9NPF2	CHST11	Carbohydrate sulfotransferase 11	-	2.738525578	1.1247	7.844367744	-	-
Q07065	CKAP4	Cytoskeleton-associated protein 4	3.3904	5.191299382	4.6214	18.68984207	-	-
O14967	CLGN	Calmegin	4.1263	3.855644664	1.3099	10.42850737	-	0.81294204
Q96DZ5	CLIP3	CAP-Gly domain-containing linker protein 3	-	1.784598911	3.9795	-	-	-
Q6PJW8	CNST	Consortin	6.5761	2.677447857	1.5722	14.04987706	0.4262	-
Q9UI42	CPA4	Carboxypeptidase A4	0.34313	3.707136237	-	4.388081969	0.14655	5.35589952
Q9HCP0	CSNK1G1	Casein kinase I isoform gamma-1	-	5.228211429	2.0426	10.77423665	3.7876	2.22227161
P78368	CSNK1G2	Casein kinase I isoform gamma-2	-	2.348465278	1.8179	6.315523557	-	2.06189818
Q9Y6M4-3	CSNK1G3	Casein kinase I isoform gamma-3	-	-	5.1398	8.00448251	-	-
Q9GZU7-2	CTDSP1	Carboxy-terminal domain RNA polymerase II polypeptide A small phosphatase 1	10.88	3.390520106	3.0472	9.285051068	2.2822	0.97446891
P78310-2	CXADR	Coxsackievirus and adenovirus receptor	-	-	2.0638	7.402472426	-	-
Q9Y4D1	DAAM1	Disheveled-associated activator of morphogenesis 1	-	-	1.7934	6.509145349	-	-
Q8NCG7	DAGLB	Sn1-specific diacylglycerol lipase beta	-	-	0.97256	5.613877505	2.4191	3.10925937
Q8IWE4	DCUN1D3	DCN1-like protein 3	-	2.792984024	2.0012	4.55996352	-	-
Q9BSY9	DESI2	Desumoylating isopeptidase 2	3.5925	6.518904824	1.6911	9.186110601	-	-
Q9Y2H0	DLGAP4	Disks large-associated protein 4	3.0485	-	0.73245	9.378223764	-	-
Q9H3Z4	DNAJC5	DnaJ homolog subfamily C member 5	6.3101	4.423408679	2.1511	8.697921197	1.8312	2.82565697
Q14126	DSG2	Desmoglein-2	-	2.782492557	1.3883	6.893699159	-	-
O00559	EBAG9	Receptor-binding cancer antigen expressed on SiSo cells	16.517	13.41849606	3.9356	17.61369641	-	-
P42892-3	ECE1	Endothelin-converting enzyme 1	4.6406	3.617028972	2.2127	7.47551768	-	-
P42892	ECE1	Endothelin-converting enzyme 1	-	-	-	-	2.8864	2.01633229
O75071	EFCAB14	EF-hand calcium-binding domain-containing protein 14	-	3.706037134	1.4858	7.519927809	-	-
Q6PCB8	EMB	Embigin	5.0413	4.467875972	2.4819	7.438815741	-	-
Q96RT1-7	ERBB2IP	Protein LAP2	7.1745	3.71940787	-	-	2.208	3.12773677
Q969X5	ERGIC1	Endoplasmic reticulum-Golgi intermediate compartment protein 1	2.7667	2.37428178	2.0485	3.12022216	1.4572	1.02853146
Q9Y282	ERGIC3	Endoplasmic reticulum-Golgi intermediate compartment protein 3	11.148	4.890932212	2.3172	30.54367746	4.336	2.64005491
P05413	FABP3	Fatty acid-binding protein, heart	-	-	-	6.162188809	0.17359	12.7135883
A8MVW0	FAM171A2	Protein FAM171A2	-	1.782690079	1.8011	4.358817889	-	-
Q8N128	FAM177A1	Protein FAM177A1	5.6223	6.78794461	1.9932	11.30978636	-	-
Q9NUQ9	FAM49B	Protein FAM49B	1.5986	1.909745431	1.3181	1.920122888	2.4214	1.84600617
P25445	FAS	Tumor necrosis factor receptor superfamily member 6	12.667	7.054673721	1.9929	7.803964414	3.1657	0.70204999
P02671	FGA	Fibrinogen alpha chain	-	4.880667675	-	-	0.35685	7.54034082
P02675	FGB	Fibrinogen beta chain	-	2.113986132	-	-	1.5941	2.0949867
O75955	FLOT1	Flotillin-1	-	-	1.5087	8.445232666	2.5506	1.86811134
Q14254	FLOT2	Flotillin-2	-	-	0.83722	12.24619756	2.6952	2.53318472
P06241-3	FYN	Tyrosine-protein kinase Fyn	-	-	-	-	2.6307	2.70848569
Q10472	GALNT1	Polypeptide N-acetylgalactosaminyltransferase 1	-	-	2.4491	5.299417064	-	-
Q8IXK2	GALNT12	Polypeptide N-acetylgalactosaminyltransferase 12	-	-	2.0878	9.559315553	2.2892	-
Q9NY12	GAR1	H/ACA ribonucleoprotein complex subunit 1	1.793	-	0.88743	1.853499407	-	-
Q14344	GNA13	Guanine nucleotide-binding protein subunit alpha-13	7.0299	2.482868209	1.697	10.75696783	3.4692	2.81214848
P30679	GNA15	Guanine nucleotide-binding protein subunit alpha-15	-	2.86377044	2.3164	15.95048968	-	1.04707656
P50148	GNAQ	Guanine nucleotide-binding protein G(q) subunit alpha	8.932	4.96056352	1.5043	19.27599368	6.759	4.80746118
Q7Z5G4	GOLGA7	Golgin subfamily A member 7	13.575	19.56525992	6.5033	53.00259713	1.4748	13.6041465
Q14789	GOLGB1	Golgin subfamily B member 1	2.2545	1.940202945	1.2212	5.561735261	-	0.43472591
P07203	GPX1	Glutathione peroxidase 1	4.5595	3.378834978	1.8988	6.559958016	1.0219	0.82528679
Q8WW33	GTSF1	Gametocyte-specific factor 1	3.8308	0.982414776	0.78501	3.354016435	-	3.03057854
P01889	HLA-B	HLA class I histocompatibility antigen, B-7 alpha chain	6.5728	9.92358837	2.8746	21.67128988	1.6652	1.73364308
P30504	HLA-C	HLA class I histocompatibility antigen, Cw-4 alpha chain	6.7543	3.318510652	3.7114	18.86756854	0.96048	4.47187193
P00738	HP	Haptoglobin	-	10.00750563	-	-	0.091044	5.70287995

UniProt Accession Number	Gene Name	Protein	Isotopic Ratio (n = 6) Sample 1	Sample 2	Sample 3	Sample 4	Sample 5	Sample 6
P01112	HRAS	GTPase HRas	3.3977	1.972581122	2.5136	7.012622721	3.8985	4.07182703
P13598	ICAM2	Intercellular adhesion molecule 2	9.1994	2.26418512	2.09	20.91044058	-	1.50407605
Q01629	IFITM2	Interferon-induced transmembrane protein 1	1.7107	3.275252194	-	17.18065458	-	-
P17181	IFNAR1	Interferon alpha/beta receptor 1	-	-	2.8275	2.915961976	-	-
P11717	IGF2R	Cation-independent mannose-6-phosphate receptor	7.1809	2.983204558	2.0151	9.973072704	0.83999	0.87896634
Q969P0	IGSF8	Immunoglobulin superfamily member 8	3.2253	1.750516402	1.2916	9.138261903	1.7519	2.21214467
P24001-4	IL32	Interleukin-32	20.148	5.178127589	-	-	-	-
Q71H61	ILDR2	Immunoglobulin-like domain-containing receptor 2	-	-	-	-	3.8548	3.95131974
Q9H0X4	ITFG3	Protein ITFG3	-	5.448403618	1.9304	8.074935401	1.9025	2.15410465
P23229-4	ITGA6	Integrin alpha-6	4.465	3.569388921	1.9296	4.272043746	-	-
O43736	ITM2A	Integral membrane protein 2A	11.25	8.739730816	3.7188	18.30362046	0.15738	5.9407117
Q9Y287	ITM2B	Integral membrane protein 2B	4.6806	2.569967361	2.5127	16.06425703	0.19134	1.78740594
Q9NQX7	ITM2C	Integral membrane protein 2C	6.4667	4.090146836	1.1003	10.82063712	-	-
Q9BX67	JAM3	Junctional adhesion molecule C	18.772	3.956165684	3.1739	19.79805979	-	-
Q8IZA0	KIAA0319L	Dyslexia-associated protein KIAA0319-like protein	1.4647	3.665017409	1.8945	9.974067425	-	-
A2VDJ0	KIAA0922	Transmembrane protein 131-like	-	-	-	1.869648132	0.38635	2.00232269
Q8IYS2	KIAA2013	Uncharacterized protein KIAA2013	4.2068	4.499032708	1.9493	9.55474871	-	1.53579162
P43630	KIR3DL2	Killer cell immunoglobulin-like receptor 3DL2	5.1184	3.766620212	1.7172	9.249838128	0.99442	1.80648891
Q6UWL6-3	KIRREL2	Kin of IRRE-like protein 2	-	2.799160252	-	4.645544922	-	-
Q6GTX8	LAIR1	Leukocyte-associated immunoglobulin-like receptor 1	3.8571	3.509018177	1.3988	8.486803021	-	-
Q6IAA8	LAMTOR1	Ragulator complex protein LAMTOR1	10.792	5.797101449	2.4898	35.85643085	4.9782	3.78300673
O43561	LAT	Linker for activation of T-cells family member 1	6.7578	3.591825006	6.2919	39.47108743	-	-
P06239	LCK	Tyrosine-protein kinase Lck	1.9895	2.011424893	1.0827	6.097932801	2.0917	1.97694878
Q9H400	LIME1	Lck-interacting transmembrane adapter 1	2.9647	10.99323916	1.8727	22.89220063	0.79087	3.50704917
Q8IWU2	LMTK2	Serine/threonine-protein kinase LMTK2	-	-	1.8794	5.994485074	-	-
Q9UIQ6-3	LNPEP	Leucyl-cystinyl aminopeptidase	6.7395	3.03039486	-	-	-	-
Q9Y561	LRP12	Low-density lipoprotein receptor-related protein 12	-	3.216571778	0.81674	8.805142203	-	-
Q9BTT6	LRRC1	Leucine-rich repeat-containing protein 1	6.1882	4.087806074	2.0843	17.07475327	2.3535	2.60633862
P20645	M6PR	Cation-dependent mannose-6-phosphate receptor	10.263	5.243288591	1.3161	35.82560097	4.7652	5.87889477
Q9UKM7	MAN1B1	Endoplasmic reticulum mannosyl-oligosaccharide 1,2-alpha-mannosidase	3.4462	2.370960476	1.567	8.577800652	-	1.23800681
Q68D91	MBLAC2	Metallo-beta-lactamase domain-containing protein 2	5.4226	7.85792865	2.0568	14.04908751	2.0863	1.40120784
Q9GZU1	MCOLN1	Mucolipin-1	2.021	-	0.14993	-	2.8245	2.55460467
Q29980	MICB	MHC class I polypeptide-related sequence B	-	-	2.3403	6.629101757	-	-
Q8IVH4	MMAA	Methylmalonic aciduria type A protein, mitochondrial	-	1.975972178	1.5708	2.636783125	0.83439	2.06778189
Q9BRT2	MNF1	Mitochondrial nucleoid factor 1	23.673	12.29377198	2.5562	67.03311436	3.5282	4.84284953
P05164	MPO	Myeloperoxidase	0.13	3.326458652	-	-	-	3.07021584
Q8N565	MREG	Melanoregulin	12.747	0.651508242	3.6642	12.4331717	3.1671	4.01445203
Q9Y3D2	MSRB2	Methionine-R-sulfoxide reductase B2, mitochondrial	-	2.323042256	-	6.653802648	-	0.90925623
Q86UE4	MTDH	Protein LYRIC	9.8282	5.525472428	2.0559	20.55582963	1.6771	2.23813787
O75570	MTRF1	Peptide chain release factor 1, mitochondrial	-	2.358601821	1.2648	4.510396464	-	-
Q96S97	MYADM	Myeloid-associated differentiation marker	-	-	-	7.466029565	1.0477	2.32018561
Q92542	NCSTN	Nicastrin	6.3118	4.637358561	2.1721	3.314550878	1.5015	2.10921516
O14561	NDUFAB1	Acyl carrier protein	-	5.340739158	-	-	3.7246	2.56121299
O15118	NPC1	Niemann-Pick C1 protein	6.5396	2.051239975	1.9427	12.32559286	2.3195	3.44376334
P01111	NRAS	GTPase NRas	4.7295	3.695354939	1.1609	15.47819896	-	-
Q9NWU1	OXSM	3-oxoacyl-[acyl-carrier-protein] synthase, mitochondrial	-	2.535882741	2.0647	8.952551477	1.8434	3.2528788
Q9UKS6	PACSIN3	Protein kinase C and casein kinase substrate in neurons protein 3	3.0397	1.212944544	2.1974	5.25265259	-	1.5157716
Q9NWQ8	PAG1	Phosphoprotein associated with glycosphingolipid-enriched microdomains 1	6.488	3.308738378	2.0375	12.3542202	2.823	1.5639173
O00330	PDHX	Pyruvate dehydrogenase protein X component, mitochondrial	8.6809	7.661661048	2.6252	13.16898441	3.1647	3.64657404
P16284	PECAM1	Platelet endothelial cell adhesion molecule	-	-	1.9553	11.10457175	-	-
Q9BTU6	PI4K2A	Phosphatidylinositol 4-kinase type 2-alpha	9.1128	4.709428275	2.0435	12.96932754	2.7377	1.73731758
Q8TCG2	PI4K2B	Phosphatidylinositol 4-kinase type 2-beta	4.062	3.67606514	2.3816	14.17273732	-	2.17798493
Q99569	PKP4	Plakophilin-4	-	1.755617978	4.4008	6.564264146	-	-
O15162	PLSCR1	Phospholipid scramblase 1	6.5093	4.660266567	2.268	10.3943621	3.772	2.00128082
Q9NRY6	PLSCR3	Phospholipid scramblase 3	4.1738	3.160256613	6.0434	7.100759781	2.0556	1.46730837
O00592	PODXL	Podocalyxin	44.543	0.471120324	9.2956	4.376559149	-	-
A8CG34	POM121C	Nuclear envelope pore membrane protein POM 121C	2.5956	1.491357583	2.1121	4.463090244	-	-
P49768	PSEN1	Presenilin-1	-	-	-	2.172590597	1.8599	1.78536359
Q13308	PTK7	Inactive tyrosine-protein kinase 7	5.89	6.342360627	1.6623	7.04920344	0.94324	1.78986934
P15151-3	PVR	Poliovirus receptor	15.068	11.19758132	4.0131	13.80891227	8.1555	1.67073211
P11234	RALB	Ras-related protein Ral-B	-	-	1.6271	1.824018678	2.8843	5.56173526
P61225	RAP2B	Ras-related protein Rap-2b	4.8701	5.580668564	2.9996	12.53321301	4.9271	5.10152025
Q9Y3L5	RAP2C	Ras-related protein Rap-2c	3.558	3.879126421	2.7232	10.92466352	5.191	4.90797546
Q00765	REEP5	Receptor expression-enhancing protein 5	4.9344	2.743710045	1.4621	7.125044532	1.3941	1.86379394
Q8IUW5	RELL1	RELT-like protein 1	-	-	2.0012	17.58674663	-	-
Q14699	RFTN1	Raftlin	1.9438	1.984875251	1.5233	6.611133148	-	-
P22670	RFX1	MHC class II regulatory factor RFX1	5.0228	3.059507419	1.9136	8.379420144	-	-
P49795	RGS19	Regulator of G-protein signaling 19	4.9352	1.850857873	1.1821	5.231493591	-	-
Q6NTF9	RHBDD2	Rhomboid domain-containing protein 2	-	-	-	5.434191936	1.6665	2.07991015
O75695	RP2	Protein XRP2	1.0866	-	1.6658	3.844675125	1.84	2.72851296
P10301	RRAS	Ras-related protein R-Ras	3.0007	1.539788125	2.9135	9.703085581	1.9669	3.45972876
P62070	RRAS2	Ras-related protein R-Ras2	3.0564	4.318721658	2.5778	11.09853277	2.9182	1.51155584
Q96DX4	RSPRY1	RING finger and SPRY domain-containing protein 1	-	-	-	3.114003675	2.4263	2.27061148
Q99590	SCAF11	Protein SCAF11	1.9227	1.051049473	0.69679	2.091787642	4.2763	-
O15127	SCAMP2	Secretory carrier-associated membrane protein 2	9.557	3.802570538	1.9551	12.28063712	2.0845	2.34620618
O14828	SCAMP3	Secretory carrier-associated membrane protein 3	5.2933	3.25203252	1.7562	14.78305861	2.4336	4.20751462
Q8WTV0	SCARB1	Scavenger receptor class B member 1	-	3.865182437	1.6894	3.465603881	-	-
Q14108	SCARB2	Lysosome membrane protein 2	4.7722	3.688811834	2.0014	5.287368477	1.0937	3.16335569
Q14160	SCRIB	Protein scribble homolog	5.9106	3.768039489	2.1716	10.2393971	-	-
Q6IQ49-3	SDE2	Protein SDE2 homolog	2.0809	-	0.93227	2.000240029	-	-
Q92854	SEMA4D	Semaphorin-4D	22.693	3.705625139	2.1099	6.962818549	1.0888	1.90389155
Q9NRX5	SERINC1	Serine incorporator 1	-	-	-	16.94053871	0.41537	2.50337956
Q587I9	SFT2D3	Vesicle transport protein SFT2C	2.36	-	-	5.316038488	-	-
Q6IA17	SIGIRR	Single Ig IL-1-related receptor	2.2138	2.086985558	2.159	5.980861244	-	-
P41440	SLC19A1	Folate transporter 1	-	-	-	4.458513532	1.1047	2.33448501
Q15758	SLC1A5	Neutral amino acid transporter B(0)	7.741	4.032420662	1.8877	20.28562155	2.4348	3.29804426
Q8TB61-3	SLC35B2	Adenosine 3-phospho 5-phosphosulfate transporter 1	-	-	-	6.547930854	1.1332	3.27944118
Q96QD8	SLC38A2	Sodium-coupled neutral amino acid transporter 2	-	-	-	8.126777733	0.84689	2.01686096
Q9ULF5	SLC39A10	Zinc transporter ZIP10	-	-	2.4551	6.655131106	-	-
Q8NBI5	SLC43A3	Solute carrier family 43 member 3	-	-	-	9.500285009	2.0615	1.99896054
Q8WWI5	SLC44A1	Choline transporter-like protein 1	-	-	0.89648	13.53088424	3.3028	2.66297401
P30825	SLC7A1	High affinity cationic amino acid transporter 1	4.176	3.385813442	1.5932	10.54362954	1.0858	2.21680337
Q92581-3	SLC9A6	Sodium/hydrogen exchanger 6	-	-	-	8.010894817	3.2584	3.04747973

UniProt Accession Number	Gene Name	Protein	Isotopic Ratio (n = 6)					
			Sample 1	Sample 2	Sample 3	Sample 4	Sample 5	Sample 6
O00161	SNAP23	Synaptosomal-associated protein 23	10.414	4.488128899	2.3253	18.29156759	4.3844	5.91715976
P60880-2	SNAP25	Synaptosomal-associated protein 25	3.4665	3.350757271	-	-	-	-
Q9Y5W9	SNX11	Sorting nexin-11	2.9044	-	1.5794	4.959579428	-	-
Q99523	SORT1	Sortilin	11.29	2.281594378	2.3598	10.14754531	3.404	1.46918387
Q7Z698	SPRED2	Sprouty-related, EVH1 domain-containing protein 2	-	-	-	-	4.7168	3.56328392
O43609	SPRY1	Protein sprouty homolog 1	2.6019	-	-	-	1.7752	1.26646403
Q5W111	SPRYD7	SPRY domain-containing protein 7	2.0315	4.585052728	2.9239	8.113590264	2.0963	3.80517504
Q9NZ72	STMN3	Stathmin	14.459	6.808742425	2.4879	17.65692593	1.0288	3.78988858
O60499	STX10	Syntaxin-10	13.085	4.153513873	1.6323	28.50058426	-	1.19587185
O75558	STX11	Syntaxin-11	-	-	2.4008	10.49406036	-	-
Q86Y82	STX12	Syntaxin-12	10.046	3.481772919	2.7637	8.4324142	1.0347	1.45785346
P32856-3	STX2	Syntaxin-2	-	-	2.9654	8.840951286	-	-
Q12846	STX4	Syntaxin-4	-	-	3.1131	29.65071458	-	1.32553916
O43752	STX6	Syntaxin-6	13.814	4.794093677	3.0489	37.82434375	-	0.58173357
O15400	STX7	Syntaxin-7	5.02	3.891656289	3.7568	21.07437146	0.93711	1.39120757
Q9UNK0	STX8	Syntaxin-8	4.5759	5.280109826	3.5346	9.954210631	2.3725	1.52870137
Q8NHG7	SVIP	Small VCP/p97-interacting protein	5.69	8.150623523	-	-	2.0745	5.55864369
Q96BS2	TESC	Calcineurin B homologous protein 3	12.435	11.91738866	3.9911	17.52879104	1.6251	1.09140518
P02787	TF	Serotransferrin	-	5.679559266	-	-	0.17545	6.64054718
Q9H5Q4	TFB2M	Dimethyladenosine transferase 2, mitochondrial	2.8323	1.959094115	1.9921	5.524861878	0.68928	0.9569378
Q13445	TMED1	Transmembrane emp24 domain-containing protein 1	6.9403	5.141916907	2.9343	8.742787201	-	1.39118821
Q9H6X4	TMEM134	Transmembrane protein 134	-	-	2.4176	6.346385733	-	-
Q8IY95	TMEM192	Transmembrane protein 192	4.2138	4.174145344	2.9166	13.06130979	-	-
Q9H813	TMEM206	Transmembrane protein 206	7.0185	5.690224195	2.7283	17.13473039	-	-
O94886	TMEM63A	Transmembrane protein 63A	-	-	-	7.602250266	3.9462	6.30040323
Q96BF3	TMIGD2	Transmembrane and immunoglobulin domain-containing protein 2	-	1.172374175	2.2973	4.160079874	-	-
Q9H3N1	TMX1	Thioredoxin-related transmembrane protein 1	13.552	5.778008898	1.1153	15.13317191	3.7697	4.62320851
Q96JJ7	TMX3	Protein disulfide-isomerase TMX3	5.914	6.040106306	2.5085	11.48052902	1.2742	1.44098448
Q9H1E5	TMX4	Thioredoxin-related transmembrane protein 4	5.7338	5.336748853	1.7486	16.40985248	2.0131	2.83430645
P28908	TNFRSF8	Tumor necrosis factor receptor superfamily member 8	6.0808	3.260089978	3.4813	19.53811887	0.92651	-
Q9Y228	TRAF3IP3	TRAF3-interacting JNK-activating modulator	8.6536	3.55353399	2.4631	10.58761249	1.667	1.6496478
O43617	TRAPPC3	Trafficking protein particle complex subunit 3	11.761	4.040077569	0.92272	35.03485969	3.2267	2.56147541
Q8NG11	TSPAN14	Tetraspanin-14	-	6.847439058	-	12.93912143	-	3.61532899
O95858	TSPAN15	Tetraspanin-15	-	-	-	-	5.3407	2.531197
Q86UF1	TSPAN33	Tetraspanin-33	-	-	1.5255	6.512112529	-	1.87938131
P41732	TSPAN7	Tetraspanin-7	1.8956	5.377211378	2.8123	25.89197867	1.5264	3.78687469
Q8NFU3	TSTD1	Thiosulfate sulfurtransferase/rhodanese-like domain-containing protein 1	8.605	2.420838578	-	-	-	-
Q9Y3C8	UFC1	Ubiquitin-fold modifier-conjugating enzyme 1	3.0142	1.143902997	0.67553	11.00400546	2.5191	2.198672
Q15836	VAMP3	Vesicle-associated membrane protein 3	11.192	6.223549913	1.1528	22.72210861	2.2941	3.09319806
O75379	VAMP4	Vesicle-associated membrane protein 4	14.147	9.336196434	2.8978	97.70395701	4.2415	2.75269764
O95183	VAMP5	Vesicle-associated membrane protein 5	35.482	7.779679477	1.5924	-	-	-
Q8IUH4	ZDHHC13	Palmitoyltransferase ZDHHC13	-	-	-	5.106469897	2.1945	3.28558286
Q8IUH5	ZDHHC17	Palmitoyltransferase ZDHHC17	-	-	-	5.536791983	3.5278	3.17248818
Q9C0B5	ZDHHC5	Palmitoyltransferase ZDHHC5	-	-	2.1445	-	2.3957	2.80559998
